# Targeted DNA transposition *in vitro* using a dCas9-transposase fusion protein

**DOI:** 10.1093/nar/gkz552

**Published:** 2019-06-25

**Authors:** Shivam Bhatt, Ronald Chalmers

**Affiliations:** School of Life Sciences, University of Nottingham, Queen′s Medical Centre, Nottingham NG7 2UH, UK

## Abstract

Homology-directed genome engineering is limited by transgene size. Although DNA transposons are more efficient with large transgenes, random integrations are potentially mutagenic. Here we present an *in vitro* mechanistic study that demonstrates efficient Cas9 targeting of the *mariner* transposon *Hsmar1*. Integrations were unidirectional and tightly constrained to one side of the sgRNA binding site. Further analysis of the nucleoprotein intermediates demonstrated that the transposase and Cas9 moieties can bind their respective substrates independently or in concert. Kinetic analysis of the reaction in the presence of the Cas9 target–DNA revealed a delay between first and second strand cleavage at the transposon end. This step involves a significant conformational change that may be hindered by the properties of the interdomainal linker. Otherwise, the transposase moiety behaved normally and was proficient for integration *in vitro* and in *Escherichia coli*. Specific integration into the *lacZ* gene in *E. coli* was obscured by a high background of random integrations. Nevertheless, Cas9 is an attractive candidate for transposon-targeting because it has a high affinity and long dwell-time at its target site. This will facilitate a future optogenetic strategy for the temporal control of integration, which will increase the ratio of targeted to untargeted events.

## INTRODUCTION

Biotechnology and medicine are increasingly reliant on our ability to engineer the mammalian genome. Lentiviruses are useful because gene-delivery across the cell membrane is efficient and integration of the viral genome provides long-term transgene expression. However, they are mutagenic because they integrate at random sites using a mechanism similar to the cut-and-paste transposons. An alternative strategy for *ex vivo* applications is to establish genomic safe-havens for site-specific recombinases such as the phage C31 integrase or the Cre recombinase (1,2). This approach lacks flexibility because it is limited to transgene integration at a predetermined site.

Scar-less engineering of a target site must rely on the cell's homologous recombination machinery. Although desired modifications can be made very precisely, the events are rare and difficult to recover. One way to boost the rate of recombination by orders of magnitude is to make a DNA double strand break at the target site (3). This can be achieved using zinc finger nucleases and transcription activator-like effector (TALE) nucleases, which can themselves be engineered to target a user-defined site (4,5). Two significant problems are the extended lead-times and off-target cleavages. TALE and zinc finger nucleases have now been largely superseded by the Cas9 nuclease. It can be programed to target a 20 bp recognition sequence, simply by providing a matching guide RNA (gRNA). Off-target cleavage is relatively low and the long recognition sequence provides ample specificity. However, homologous recombination remains a limiting factor because the efficiency falls off as the transgene size increases (6).

Transposon vectors are widely used for gene delivery applications (7–11). Although their efficiency falls off as the cargo size is increased, they are less sensitive to this parameter than host-mediated homologous recombination (12–16). However, like the Lentiviruses, transposon vectors are mutagenic because the integration sites are essentially random.

To combat random integration various groups have attempted to use site-specific DNA binding proteins to target specific loci. Examples include *Mos1, Sleeping Beauty, piggyBac* and *ISY100* transposases, which were variously fused to zinc finger proteins, TALEs and Gal4 (17–22). Cas9 is an obvious and attractive candidate for targeting because extensive base pairing with the target provides a dwell time of several hours (23). In contrast, most DNA binding proteins remain bound to their specific sites only for a matter of seconds or minutes.

Previously, there was an unsuccessful attempt to use a catalytically inactive Cas9 (dCas9) to target *piggyBac* insertions to a hypoxanthine phosphoribosyl transferase (*HPRT*) gene in human cells (19). Surprisingly, instead of targeting the *HPRT* locus, the dCas9-piggyBac chimera protected it from insertions. For targeting experiments, the choice of the transposase moiety is limited by several factors. Although *Tn5* is very active *in vitro*, and is used extensively in bacteria, it transposes poorly in mammalian cells (24). *Sleeping Beauty* is efficient *in vivo* but lacks an *in vitro* system (9). While *piggyBac* is probably the most efficient for gene delivery *in vivo*, it has a poor *in vitro* system (10,25), which precludes the development of advanced applications such as direct delivery of transpososomes.

Our model system is based on *Hsmar1*, a reconstituted *mariner*-family transposon (26,27). In HeLa cells, *Hsmar1* transposition is less than half as active as *Sleeping Beauty* and *piggyBac* (16). Nevertheless, it is an excellent model system for *in vitro* mechanistic studies as the reaction is 100% efficient *in vitro* (28). Furthermore, if *mariner* transpososomes are assembled *in vitro*, they can be delivered to cells directly [Ref. (29) and our unpublished experiment). *Hsmar1* transposase also has much lower non-specific nuclease activity than other *mariner* transposases such as *Mos1, Mboumar1* and *Himar1* (30–35). Here we show that the Cas9 and *Hsmar1* transposase moieties of a fusion protein are both active and report a targeting efficiency of more than 50%.

## MATERIALS AND METHODS

DNA oligonucleotides and most dry chemicals were from Sigma Aldrich. Enzymes were from New England Biolabs and DNA purification kits were from Qiagen. The nucleotide sequences of all plasmids used in this work are given in Supplemental Table S1. The β-galactosidase assays were as described by Miller (36). LB agar indicator plates contained 40 μg/ml X-gal, when present,.

For *in vivo* assays in *Escherichia coli*, dCas9 was expressed from derivatives of plasmid pdCas9 (37). When present, CRISPR spacers were cloned into the BsaI site as oligoduplexes (Supplemental Table S2). The *Hsmar1* transposase gene was added to the 5′- or 3′-end of the dCas9 gene by polymerase chain reaction (PCR) using pdCas9 and pRC880 as templates: transposase-dCas9, pRC2302; dCas9-transposase, pRC2303. An oligoduplex encoding spacer-7 was cloned into the CRISPR locus of these plasmids to produce pRC2304 and pRC2305, respectively. The native wild-type transposase was expressed from pRC2306, which is identical to pdCas9 except that the dCas9 gene is replaced by the transposase gene.

The transcription-strength series of expression vectors for Figure 2C and D was created using the constitutive promoters described by Tellier and Chalmers (38). In the plasmid with the strongest promoter, P4 (pRC2309), the native *Streptococcus pyogenes* Cas9 promoter in pdCas9 was replaced by Tellier-P2. In plasmid P3 (pRC2308) the *S. pyogenes* promoter was replaced by Tellier-P1. In plasmid P2 (pRC2307) the *S. pyogenes* promoter was deleted by inverse PCR and residual transcription was from a cryptic promoter(s). The plasmid with the lowest level of transcription, P1 (pRC2311), was created by replacing the multi-copy origin-of-replication in plasmid P3 (above) with a single-copy origin-of-replication from a bacterial artificial chromosome vector.

*Hsmar1* transposase was expressed from pRC880 and purified as a maltose binding protein fusion by affinity chromatography as described (33,39). The expression vector for the dCas9-transposase fusion protein was pRC2303 described above.

The dCas9-transposase fusion-protein was expressed from the constitutive promoter in pRC2303. The plasmid was transformed into *E. coli* NiCo21 (New England Biolabs). A single colony was picked into a starter culture of LB-Lennox supplemented with 30 μg/ml chloramphenicol and grown at 37°C for 16 h with shaking at 250 rpm in a baffled Erlenmeyer flask. The culture was diluted 1:100 into fresh media and growth was continued until the OD600 reached 0.5. The temperature was then reduced to 18°C and the incubation was continued for 16 h. Cells (500 ml) were harvested by centrifugation at 4°C and resuspended in 40 ml binding buffer (20 mM Tris, pH 8.0, 5 mM imidazole, 0.5 M sodium chloride, 2 mM dithiothreitol (DTT)). All subsequent steps were carried out at 4°C. Cells were lysed by five passes through a French pressure cell at 16 000 psi and the extract was clarified by centrifugation at 40 000 × *g* for 30 min. The clarified extract was applied to a 1 ml HisTrap HP column (GE Life Sciences) on an AKTA FPLC (Amersham Pharmacia). The column was equilibrated with binding buffer and loosely bound proteins were eluted with 40 column volumes of wash buffer (20 mM Tris at pH 8.0, 60 mM imidazole, 0.5 M sodium chloride, 2 mM DTT). The column was then developed with a 20 column-volume linear-gradient up to 1 M imidazole. Peak fractions were pooled an analyzed by sodium dodecyl sulphate (SDS) polyacrylamide gel electrophoresis (Supplementary Figure S1). When required for *in vitro* reactions, the sgRNA and Cas9-transposase were assembled into a ribonucleoprotein complex by incubating for 20 min at room temperature in dCT binding buffer (20 mM HEPES pH 7.5, 250 mM potassium chloride, 2 mM MgCl2, 0.01% Triton X-100, 0.1 mg/ml bovine serum albumin (BSA), 10% glycerol) (40). This was done every time it was required immediately before it was used.

The electrophoretic mobility shift assay (EMSA) binding buffers for the native transposase and the dCas9-transposase were, respectively, [20 mM HEPES at pH 7.5, 100 mM NaCl, 2 mM DTT, 10% glycerol, 5% dimethyl sulfoxide (DMSO), 5 mM CaCl2, 250 μg/ml BSA] and [20 mM HEPES pH 7.5, 250 mM potassium chloride, 2 mM MgCl2, 0.01% Triton X-100, 0.1 mg/ml BSA, 10% glycerol]. Reactions (20 μl) contained 10 nM of a Cy-5 labeled oligoduplex encoding either the transposon end or the target of the sgRNA (Supplementary Table S3). After addition of the dCas9-sgRNA-transposase complex, the reactions were incubated for 20 min at 37°C. Binding reactions with the native transposase were incubated for 60 min at room temperature to be consistent with our previous work. Binding reactions were loaded onto a trisaminomethane/borate/ethylenediaminetetraacetic acid (TBE)-buffered 7% polyacrylamide gel and electrophoresed at 120 V. Gels were recorded on a Fujifilm FLA-3000 Fluorescence Laser Imaging Scanner.

*In vitro* transposition reactions were performed as described (33). The reaction buffer contained 25 mM Tris–HCl at pH 8.0, 5% glycerol, 100 mM sodium chloride, 2 mM DTT, 2.5 mM MgCl2 and 6.5 nM of supercoiled pRC650 as substrate. Transposition reactions (50 μl) were initiated by the addition of transposase and incubated for 4 h at 37°C. Reactions were made 0.4% in SDS and 19 mM in ethylenediaminetetraacetic acid (EDTA) and heat inactivated at 75°C for 30 min. Samples (35 μl) were electrophoresed on a TBE-buffered 1.1% agarose gel at 60 V for 16–24 h. Gels were stained with ethidium bromide and photographed. *In vivo* transposition reactions in *E. coli* were performed as described (41). The reporter strain [*E. coli* RC5096, *F- fhuA2 Δ(lacZ)r1 glnV44 e14-(McrA-) trp-31 his-1 rpsL104 xyl-7 mtl-2 metB1 Δ(mcrC-mrr)114::IS10 argE::Hsmar1-lacZ'-kanR*] was derived from ER1793 (New England Biolabs).

Targeted transposition experiments were based on a well known ‘plasmid-hop’ assay e.g. (31). The transposon donor plasmid was pRC704, which encodes an R6K origin of replication and a mini-*Hsmar1* transposon with a kanamycin resistance gene. The small target plasmid was pRC2312, which is essentially a dimer of pBluescript II SK+ with one of the copies of *lacZ*α removed. The large target was pRC2313, which is identical to the small target except that a 4.5 kb fragment of non-specific DNA from the *E. coli* chromosomal *argE-H* locus was inserted. Reactions (15 μl) were performed in our standard transposition buffer (25 mM Tris–HCl at pH 8.0, 5% glycerol, 100 mM sodium chloride, 2 mM DTT, 2.5 mM MgCl2) with 9 nM of target plasmid and 13.5 nM pRC1105 to provide a background of non-specific DNA. Reactions were incubated for 20 min at 37°C, initiated by the addition of the transposon donor plasmid (9 nM) and incubated for a further 24 h. Reactions were brought to 50 μl with NEB Cutsmart buffer, supplemented with five units of NheI and five units of lambda exonuclease to degrade the non-specific plasmid. Digestion was stopped by adding 15 μl of stop solution (0.5 mg/ml proteinase K, 0.1% SDS, 50 mM EDTA) and incubating at 60°C for 2 h. DNA was purified using a spin column from the Qiagen PCR Clean-up kit, which was eluted with 30 μl of EB (10 mM Tris–HCl, pH 8.5). A total of 3–10 μl of the reaction was transformed into chemically competent *E. coli* NEB5α and plated on LB agar plates supplemented with 50 μg/ml kanamycin, 100 μg/ml ampicillin, 40 μg/ml X-gal and 0.1% lactose. Targeting efficiency (%) was calculated by dividing the number of white colonies by the total number of colonies and multiplying by 100. Target plasmids from randomly selected white colonies were purified and the location of the transposon insertion in *lacZ*α was determined by Sanger sequencing using the M13 reverse primer.

## RESULTS

### Selection of gRNAs and construction of transposase-dCas9 fusions

**Figure 1.**
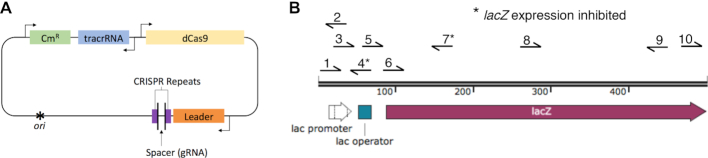
Plasmid pdCas9 and the chromosomal *lacZ* target. (**A**) Plasmid pdCas9 (37) encodes a catalytically inactive Cas9 variant (dCas9) in which the nuclease activity is abolished by the D10A and H840A mutations. Expression is driven by the strong native *Cas9* promoter from *Streptococcus pyogenes*. The spacer is the user-defined sequence that base pairs with the target site. (**B**) The target site for dCas9 binding was the *Escherichia coli lacZ* gene. Ten candidate spacers were selected to target the promoter and early open reading frame of *lacZ*. Of these 10 candidates, only spacers 4 and 7 inhibited transcription completely, as judged by a white colony on X-gal indicator plates.

Since gRNAs do not all bind their targets equally well (42) we screened ten candidates for their ability to inhibit transcription of the target *lacZ* gene. Oligoduplex spacer sequences, encoding candidate gRNAs, were ligated into the CRISPR locus of plasmid pdCas9 (Figure 1A). Subsequent transcription and processing *in vivo* produces a mature gRNA, which forms a ribonucleoprotein complex with dCas9. The relative locations and orientations of the selected gRNA target-sequences are shown in Figure 1B. *Escherichia coli* BL21 cells were transformed with each dCas9-gRNA expression plasmids separately and spread on X-gal indictor plates to test the ability of the gRNAs to inhibit transcription of *lacZ*. Only spacers 4 and 7 produced completely white colonies (not shown). Because spacer-7 is within the coding sequence of the gene, it was selected for all further experiments. To create a fusion protein capable of targeted transposition, the *Hsmar1* transposase gene was added to the 5′- or the 3′- end of the *dCas9* gene in pdCas9 (Figure 1A). Two versions of the fusion protein expression vectors were created, one with and one without spacer-7.

### The fusion-proteins are active in *E. coli*

**Figure 2.**
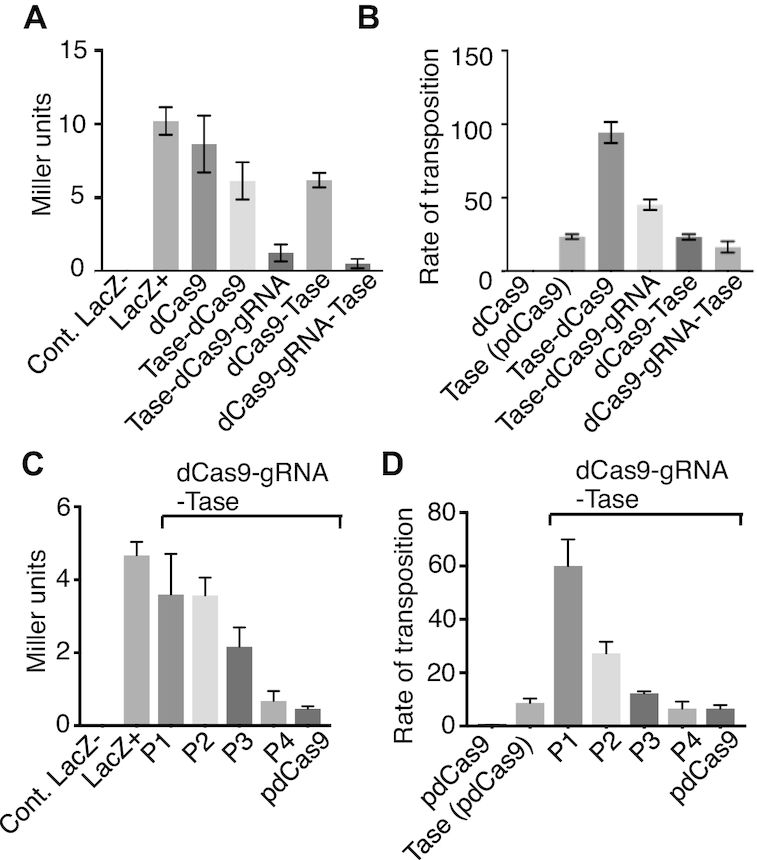
Fusion-protein activity *in vivo*. The *Hsmar1* transposase gene was added to the 5′- or the 3′- end of the dCas9 gene in pdCas9, which is illustrated in Figure 1A. (**A**) The plasmids were transformed into *Escherichia coli* BL21 and *lacZ* activity was measured using the Miller assay (36). The *lacZ*- control was provided by *E. coli* NEB5α, while *LacZ*+ is the activity with the untransformed BL21 host. Proteins were expressed from plasmids: dCas9, pdCas9 = pRC2301; transposase-dCas9, pRC2302; transposase-dCas9-gRNA, pRC2304; dCas9-transposase, pRC2303; dCas9-gRNA-transposase, pRC2305. (**B**) The relative rates of transposition supported by the N- and C-terminal fusions was assayed in *E. coli* as described previously (41). The wild-type transposase was expressed from pRC2306, which is identical to pdCas9 except that the dCas9 gene is replaced by the transposase gene. Other protein expression vectors were as in part A. (**C**) The relationship between *lacZ* repression and the fusion-protein expression level was explored. The highest level of expression was with the native Cas9 promoter form pdCas9. Promoters P4 to P1 were progressively weaker (38). The expression vectors were: P1, pRC2311; P2, pRC2307; P3, pRC2308; P4, pRC2309. (**D**) The relationship between the expression level and the rate of transposition was explored. Owing to auto-inhibition (or OPI) the highest rate of transposition was with the lowest expression level, as expected (16). Protein expression vectors were as in previous parts.

To test whether the fusion proteins were still capable of gRNA-mediated transcriptional repression, the four plasmids were transformed into *E. coli* BL21 and the lacZ activity was measured using the Miller assay (Figure 2A). There was little silencing with dCas9 or either of the fusion proteins in the absence of the gRNA. In the presence of the gRNA the N- and C- terminal dCas9 fusion proteins both repressed *lacZ* expression effectively. We then used a bacterial transposition assay to test whether the transposase domain retained activity (Figure 2B). We also tested a plasmid in which the dCas9 gene in pdCas9 was substituted with the transposase gene. This provided a positive control in which transposase expression was driven by the same promoter as the fusion proteins. The rate of transposition was highest for the transposase-dCas9 plasmid, which has the transposase fused to the N-terminus of Cas9. This result should be interpreted with caution as *Hsmar1* transposition is subject to over-production inhibition (OPI) whereby an increase in the transposase concentration beyond a low level decreases the rate of transposition (16,38). Since the promoter on pdCas9 is relatively strong, the system will be dominated by OPI. In the presence of the gRNA the rate of transposition for the N- and C-terminal fusion proteins decreased significantly (Figure 2B). If this result is interpreted in terms of the OPI model, it indicates that the gRNA increased the transpositional-potential of the system, which decreased the rate of transposition on account of OPI. Since the dCas9-transposase protein, which has the transposase on the C-terminus of dCas9, had the best transpositional-potential and the best transcriptional repression in the Miller assay, it was selected for all further experiments (Figure 2A and B).

To explore the relationships between the expression level of the fusion protein and its activities, the native Cas9 promoter in pdCas9 was replaced with promoters P4 to P1, which are of progressively lower strength (38). As expected, transcriptional repression by the fusion protein was highest with the native Cas9 promoter and decreased progressively with promoter strength (Figure 2C). In contrast, the rate of transposition had an inverse relationship to the strength of the promoter, consistent with the expected effects of OPI mentioned above (16).

We also tested whether the non-replicating dCas9-transpososome would target the *lacZ* gene after electroporation into *E. coli* cells. We recovered kanamycin resistant clones with chromosomal integrations (not shown). However, targeted integration into the *lacZ* gene was obscured by a high background of random integrations, which are a recurrent problem in other similar transposon-targeting systems.

### dCas9-transposase binds transposon ends and the gRNA target

**Figure 3.**
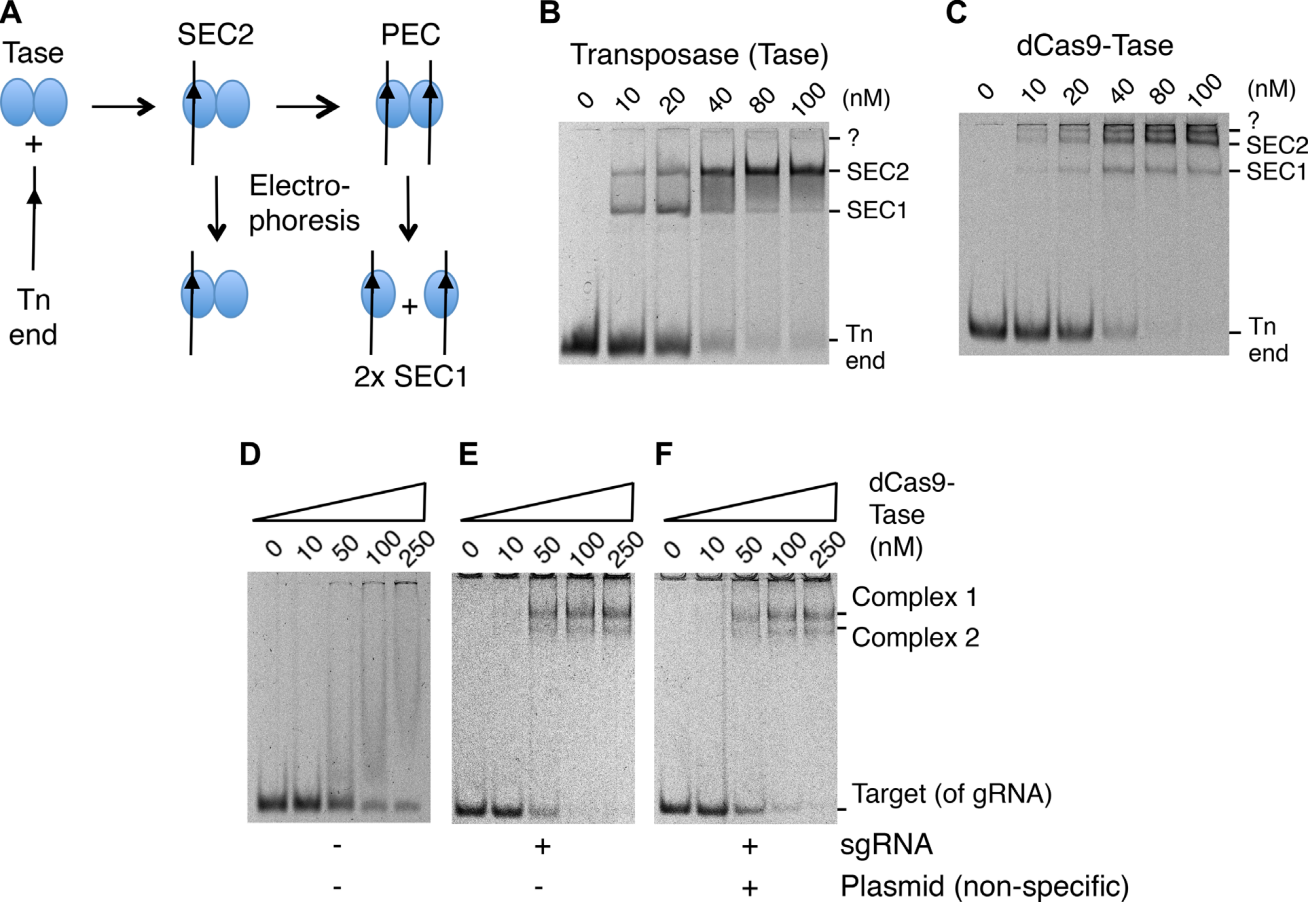
EMSA analysis of the dCas9-transposase fusion protein. (**A**) Illustration of protein–DNA complexes formed during the transposition reaction and decomposition of the PEC during electrophoresis. (**B**) EMSA of purified *Hsmar1* transposase on a TBE-buffered 7% polyacrylamide gel. Reactions contained 10 nM of a 70 bp Cy5-labeled oligoduplex encoding a transposon-end. Bands were visualized on a Fujifilm FLA-3000 Fluorescence Laser Imaging Scanner. (**C**) EMSA as in part B but with the indicated amounts of the dCas9-transposase fusion protein without sgRNA. The band marked ?, just below the wells, might represent the PEC, which is detected with the wild-type transposase if the PEC is artificially stabilized (16). (**D**–**F**) the EMSAs were as in part B but with the purified dCas9-transposase fusion protein with its sgRNA. Reactions contained 10 nM of a 70 bp Cy5-labeled oligoduplex complementary to sgRNA-7. When present, the sgRNA was preassembled in a 1:1.5 molar excess to the protein prior to the EMSA. The non-specific competitor plasmid was 1 μg of pRC2301 per binding reaction.

The assembly pathway for the *Hsmar1* transpososome and its behavior in an EMSA is illustrated in Figure 3A and B. Binding of the first transposon end to the transposase dimer yields single-end complex 2 (SEC2). Recruitment of a second end yields the paired-ends complex (PEC). This would normally be followed by the first cleavage step of the reaction, except that the binding buffer lacks the catalytic magnesium ion. During electrophoresis, the PEC decays into single-end complex 1 (SEC1) (16). When the purified dCas9-transposase is titrated into the reaction the disappearance of the free transposon ends is accompanied by the appearance of two clear shifted bands plus a third band close to the bottom of the well (Figure 3C). This is broadly similar to the behavior of the wild-type transposase except that the complexes are further up the gel owing to the much larger size of the fusion protein. The band that just enters the gel above SEC2 might represent the PEC, which is detected in some experiments with the wild-type transposase (16).

To explore the binding ability of the dCas9 moiety of the fusion protein, an EMSAs was performed with an oligoduplex complimentary to the spacer-7 sequence (Figure 3D–F). Since this experiment was performed *in vitro* we used a single-guide RNA (sgRNA) in which the tracrRNA and the crRNA were joined in a single strand. In the absence of the sgRNA, titration of the fusion protein produced a smear with no clear bands (Figure 3D). Presumably, this indicates weak interactions between the protein and the DNA, which dissociate during electrophoresis. When the fusion protein was provided with the sgRNA, two clear complexes were formed (Figure 3E). The stoichiometry of the complexes is unknown but they may correspond to a dimer of the fusion protein bound by either one or two target oligoduplexes. When we added unlabeled competitor DNA to the reaction it had no effect, which indicates that the oligoduplex binding to the fusion protein is specific (Figure 3F).

### *In vitro* transposition with the dCas9-transposase fusion-protein

**Figure 4.**
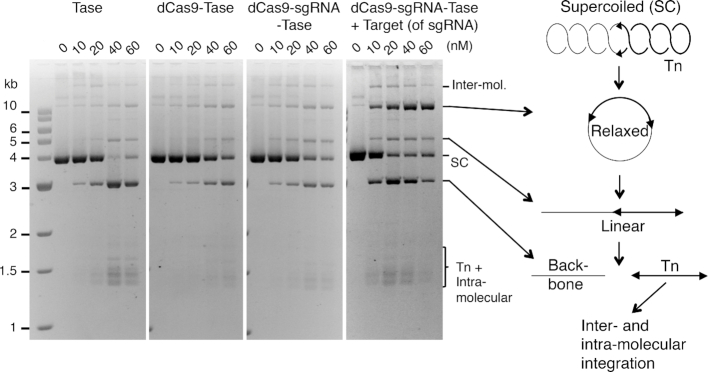
*In vitro* transposition assays with the dCas9-transposase. Transposition reactions were 50 μl with 6.5 nM of supercoiled plasmid substrate (pRC650). The reactions were initiated by adding transposase and incubated for 4 h at 37°C. Reactions were deproteinated with proteinase K and SDS and 20 μl was electrophoresed on a TBE-buffered 1.1% agarose gel at 60 V overnight. Photographs of ethidium bromide stained gels are shown. When present, the sgRNA was preassembled in a 1:1.5 molar excess to the protein prior to the reaction. Where indicated, reactions contained 10 nM of a 70 bp oligoduplex complementary to the sgRNA.

Since we had demonstrated DNA binding by the respective dCas9 and transposase moieties of the fusion protein, we next wanted to examine the intermediates and products of the *in vitro* transposition reaction. When the wild-type transposase is incubated with a supercoiled substrate, excision of the transposon leaves behind the plasmid backbone, which is an end product of the reaction and a convenient measure of the efficiency (Figure 4). After excision, target sites for integration are acquired by a random collision-and-tracking mechanism (33). Titration of the reaction with the wild-type transposase or dCas9-transposase yielded a very similar range of products (Figure 4). Minor differences were probably owing to the differences in the quality of the purified proteins. However, adding an oligoduplex encoding the target of the sgRNA reduced the amount of protein required for the maximum production of backbone. It also changed the amount and relative proportions of the reaction products. Most noticeable is the accumulation of the relaxed substrate. This is an intermediate of the reaction generated by the first nick at one of the transposon ends (33,43). The transition between the first nick and the second, which completes cleavage at the transposon end, involves a significant conformational change (28,44). Since nicking is absolutely dependent on PEC formation (39), the accumulation of the relaxed intermediate shows that the dCas9 moiety of the fusion protein can interact with its oligoduplex target at the same time as the transposase moiety is engage in a transposition reaction. Clearly, this is a prerequisite for successful targeting.

### Targeted transposition reactions *invitro*

**Figure 5.**
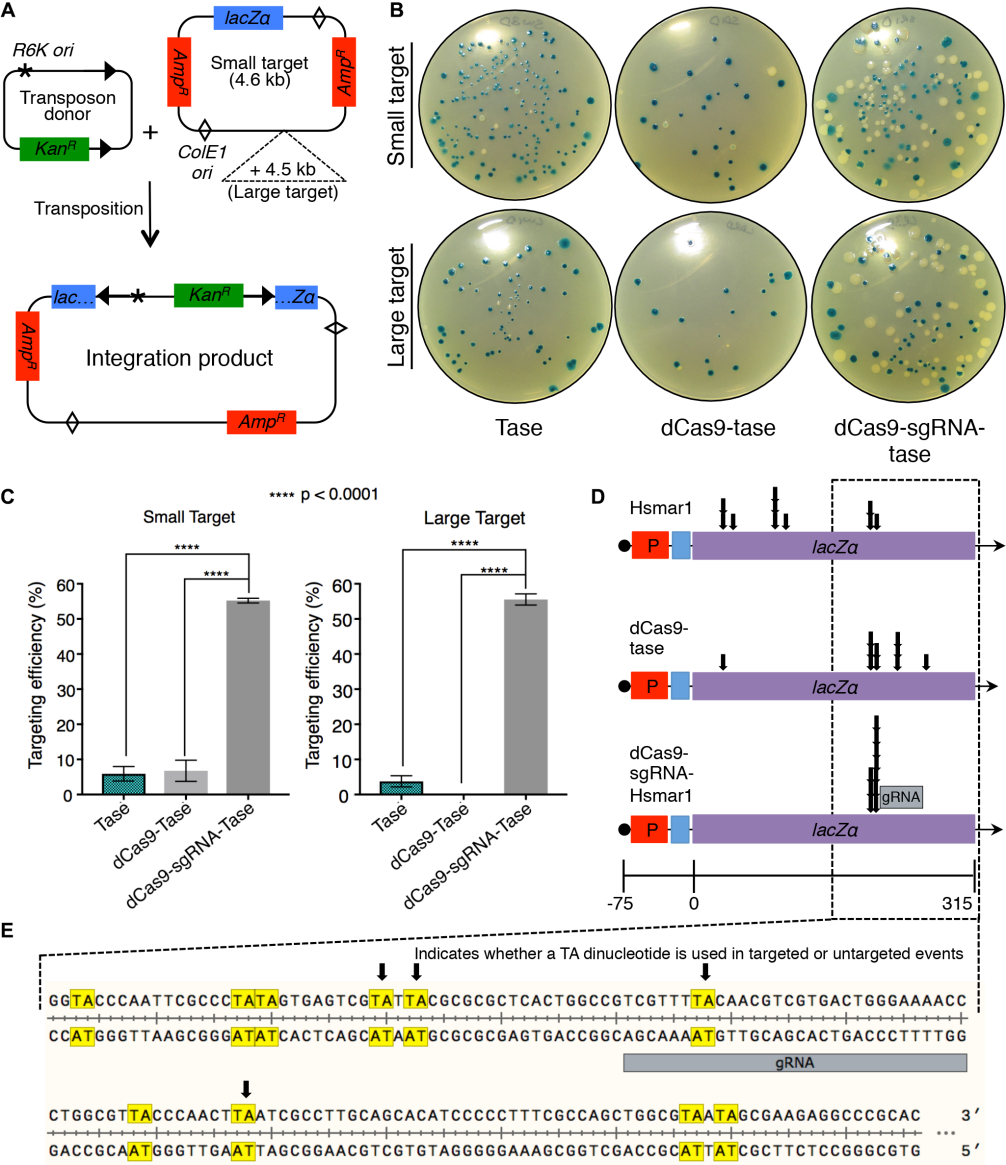
Transposon targeting reactions. (**A**) Illustration of the transposon donor and target plasmids. The donor plasmid (pRC704) encodes an *Hsmar1* transposon with an R6K origin of replication and a kanamycin resistance gene. The target plasmid (pRC2312) is essentially a dimer of pBluescript with one copy of the *lacZ*α gene removed. Since the plasmid has two origins and to ampicillin markers it can tolerate transposon insertions anywhere. The large target has an extra 4.5 kb of non-specific DNA. Reactions also contained a background of non-specific DNA. (**B**) Reactions were performed with the indicated transposase proteins, transformed into *Escherichia coli* and plated on LB plus ampicillin, kanamycin and X-gal. Transposon integrations into the *lacZ*α gene yield white colonies. Integrations elsewhere yield *lacZ*+ colonies. (**C**) Targeting efficiency. Error-bars are standard error of the mean where *n* = 6 biological replicates. Ordinary one-way ANOVA analysis: Small target; transposase versus dCas9-transposase, *P* = 0.95; transposase versus dCas9-sgRNA-transposase, *P* = <0.0001; dCas9-transposase versus dCas9-sgRNA-transposase, *P* = <0.0001. Large target: transposase versus dCas9-transposase, *P* = 0.14; transposase versus dCas9-sgRNA-transposase, *P* = <0.0001; dCas9-transposase versus dCas9-sgRNA-transposase, *P* = <0.0001. (**D**) Ten white colonies were picked from the three sets of plates and the location of the transposon insertion determined by Sanger sequencing. (**E**) An expanded view of the indicated area of the *lacZ*α gene.

To test whether the dCas9-transposase protein could target transposon insertions to a specific site *in vitro* we used a modification of the mini-transposon hop assay (31). In this assay, transposase catalyses the movement of a transposon encoding a kanamycin resistance gene onto a target plasmid, which in this case encodes the *lacZ*α fragment and an ampicillin resistance gene (Figure 5A). The target was derived from a dimer of pBluescript in which one of the two *lacZ*α fragments had been removed. Since the plasmid has two ampicillin genes and two origins, it has no essential regions and can tolerate insertions anywhere. This allows unbiased recovery of integration events. We refer to the modified pBluescript dimer as the small target. We also created a larger target by adding 4.5 kb of DNA from the *E. coli* arginine biosynthesis operon. In addition to the transposon donor and target plasmids, transposition reactions also contained a decoy plasmid which provides a background level of non-specific DNA. The reactions were assembled and then initiated by the addition of the transposon donor plasmid.

Transposition reactions were performed with the wild-type transposase and the dCas9-transposase fusion-protein either without or with sgRNA-7, which is complementary to the *lacZ*α region of the target plasmids. The integration products were transformed into *E. coli* NEB5α and plated on LB agar plates supplemented with ampicillin, kanamycin and X-gal (Figure 5B and C). Double transformants containing the donor and target plasmids are not recovered because the R6K origin of replication requires the Pir protein. Transposition reactions with the wild-type transposase or the dCas9-transposase fusion yielded very few white colonies (Figure 5B). This reflects the fact that the *lacZ*α fragment represents only about 1/650th of the DNA sequences available for integration. In contrast, more than half of the colonies are white when the dCas9-transposase protein is provided with the sgRNA. The targeting efficiency is plotted in Figure 5C where it is defined as the percentage of all colonies that were white.

In the case of the blue colonies, the transformants must have contained plasmids with transposon insertions outside of the *lacZα* region. We therefore focused on the white colonies and used DNA sequencing to determine the transposon integration sites (Figure 5D). In pBluescript the *lacZα* open reading frame (ORF) is engineered to include a polylinker and it is therefore longer than the corresponding region from the wild-type *lacZ* gene. The ORF also codes for additional amino acids at the 3′-end that are not necessary for alpha complementation (45). All in all, the target region in pBluescript in which integration events will yield a white phenotype is 315 bp long (Figure 5D). Within this region there are 18 TA dinucleotides, which are required for the integration of *mariner* transposons. Only eight of these sites are represented among the 20 un-targeted integrations mapped for the wild-type transposase and the dCas9-transposase fusion without the sgRNA (Figure 5D). This is not unusual as mild integration bias is well documented for the *mariner* transposons, where preferred sites are known as hot spots (46–48).

We next examined the *lacZ*α integration sites targeted by the dCas9-sgRNA-transposase. We found that all had occurred at two adjacent TA dinucleotides located 18–22 bp to one side of the sgRNA binding site (Figure 5D and E). On the other side of the binding site there are TA dinucleotides between 8 and 18 bp distant that were not used. The location of the integration sites immediately to one side of the binding site probably reflects constraints imposed by the relative orientations of the dCas9 and the transposase domains and the length of the linker region. It will be interesting to test the site-preference of the transposase-dCas9 fusion, in which the positions of the domains are swapped with respect to the N- and C-termini of the protein.

## DISCUSSION

Many attempts have been made to target transposon insertions to specific genomic locations. For example, one comprehensive study of *Sleeping Beauty* targeting explored a variety of approaches using direct protein-DNA binding domains and protein-protein sandwich interactions (18). This provided for a 10% targeting-efficiency to an artificially engineered chromosomal target site. In another study, *Gal4-Mos1* transposase and *Gal4-piggyBac* transposase fusions could be biased toward a UAS-containing target plasmid with 12.7- and 11.6-fold efficiency, respectively, compared to the native transposase (20). A ZFP-*piggyBac* transposase fusion provided a targeting frequency of 74% in an *in vivo* plasmid-to-plasmid assay. However, the native *piggyBac* transposase also integrated into the same window with a frequency of 50% (49). In another *in vivo* plasmid-to-plasmid assay, a *Gal4-piggyBac* transposase fusion was able to bias integrations around a UAS-containing target plasmid with ∼4-fold enrichment. After engineering the HEK-293 cell genome to contain 1 or 2 Gal4-UAS target sequences, *Gal4-piggyBac* transposase integrated close to the target site 32% of the time compared to 8% for native transposase (50). It was also demonstrated that a TALE-piggyBac fusion could direct insertions into the first intron of the human *CCR5* gene, which was detected at a frequency of ∼0.010–0.014% of total stably transfected cells (17). When the zinc-finger protein zif268 was fused to the C-terminus of the ISY100 transposase and it was shown that 50% of all integrations occurred within 20 bp of the target site. However, the target site on the target plasmid was engineered to contain nine tandemly repeated TANN transposase integration sites adjacent to the ZFP binding site (22). In a more recent study, a TALE-piggyBac transposase and ZFP-PB transposase fusion could target and integrate transposon DNA into the human *HPRT* gene at a frequency of 0.97 and 0.42%, respectively (19).

The long dwell time of dCas9 at its target site makes it a powerful tool for gene activation and repression (51). It is also an attractive candidate for transposon targeting. However, one study reported that although *piggyBac* integrations at a specific target site were enriched by zinc finger and TALE protein-fusions, dCas9 appeared to protect the locus (19). In the present work we have demonstrated that a *mariner* transposon can be effectively targeted by dCas9 and that protection from integration is probably therefore peculiar to *piggyBac* or perhaps a property of the interdomainal linker. In our case, we found that the targeted *mariner* insertions were at two adjacent TA dinucleotides about 20 bp 5′ to the sgRNA binding site (Figure 5E). Other TA dinucleotides located between 31 and 34 bp 5′, and between 8 and 18 bp 3′, of the sgRNA binding sites were not used. This indicates that the integration site is tightly constrained.

The properties of the interdomainal linker and the intrinsic target-site preference of the transposase may both contribute to the constrained selection of integration sites. Most, if not all, DNA transposons have preferred target hot spots. For example, there is one extremely hot spot for *Mos1* integration that attracts almost all events in the *Tn9* chloramphenicol acetyl transferase gene (47). *Hsmar1* transposase is perhaps less biased but some sites are certainly less preferred than others (48). The tight constraint on the integration site immediately to one side of the target has not been observed in other similar systems (see below). While these systems used relatively short flexible linkers, our linker comprised of 187 amino acids encoding the *E. coli* thioredoxin protein, which we had previously been used with great success in creating a single-chain transposase-dimer (28). A linker of this length would be very long if it was extended. However, since ours encoded elements of secondary and tertiary structure it will be quite compact, and presumably relatively resistant to proteolysis. Indeed, the structure of the linker may account for the tight constraint of the integration site immediately to one side of the dCas9 binding site (Figure 5). However, since the two targeted TA dinucleotides are 4 bp apart they will be on almost opposite faces of the DNA helix. This indicates that the angular distribution is not tightly constrained. It will be interesting to explore the effect of different interdomainal linkers and the effect of adding additional TA dinucleotides slightly closer or further away from the dCas9 binding site.

In targeting experiments with other transposase-fusion proteins the selection of integration sites was not tightly constrained as observed here. When *Mos1* or *piggyBac* transposases were fused to Gal4 using a 22 amino acid linker integration was enriched for sites about 900 bp away from the Gal4 upstream activating sequence (*UAS*). In the case of *Mos1*, 98% of the integrations were at a TA dinucleotide 954 bp from the binding site (20). Such frequent use of a site, almost 10 DNA-persistence-lengths away from the Gal4-*UAS*, can not be explained by the constraints of the binding moiety. Instead, a combination of factors may be at work. For example, the short dwell time of DNA binding proteins, which range from seconds to minutes, will increase the local concentration of the transpososome, which may then select a nearby hot spot. This scenario may also explain the partial enrichment of AAV *Rep-Sleeping Beauty* integration at a site 700 bp from the Rep recognition sequence (52). In another report, fusing the *piggyBac* transposase to zinc finger and TALE proteins with linkers as short as 15 amino acids enriched for integrations between 24 and 5000 bp from the binding site in the hypoxanthine phosphoribosyl transferase (*HPRT*) gene (17,19). Within this region, integrations were biased toward the *HPRT* transcriptional start site. This may be owing to the chromatin configuration or topological changes in the DNA. For example, *mariner* transposition is sensitive to supercoiling in the transposon donor and the target (39,48).

In addition to the targeting experiments, we also compared the reaction intermediates with the transposase and the dCas9-transposase proteins (Figures 2 and 3). In the EMSA, the nucleoprotein complexes were very similar (Figure 3). Likewise, in reactions with a plasmid substrate, the kinetics of the reaction intermediates were similar (Figure 4). However, there was a slight delay between first and second strand cleavage at the transposon end when the target of the sgRNA was included in the reaction. Overall, it is clear that the *Hsmar1* transposase behaves normally when fused to dCas9 and that dCas9 is therefore a promising system for targeted integration.

The single biggest problem in targeted-transposition protocols is that successful targeting in a particular cell is usually accompanied by random integrations. It should be possible to minimize this problem by delivering the transpososome to the cells after *in vitro* assembly (29). However, to overcome random integration events, it will be necessary to control the timing of integration to allow an opportunity for the targeting moiety to find its binding site. We are currently exploring strategies for the temporal control of transposon integration. For example, the use of optogenetics to insert a photo-labile amino acid analog in place of an essential amino acid in the *Hsmar1* transposase (53). Once these methods have been established for *Hsmar1*, the principles can be translated into *Sleeping Beauty* and *piggyBac* systems, which are more efficient methods of gene delivery in animal cells.

## NOTE ADDED IN PROOF

While this article was in press an Epub (ahead of print) by Strecker and colleagues reported that a CRISPR-associated transposase from *Scytonemahofmanni* (ShCAST) catalyzes site-specific RNA-directed integration (54). Similar to our findings, integrations were unidirectional and located a fixed distance to one side of the targeted DNA site. The ShCAST is a homolog of the Tn7 transposase from *E. coli* and their reactions are very similar except for the targeting mechanism. The TnsD subunit of Tn7 uses a direct sequence-specific protein-DNA interaction to target integrations into a unique attachment site on the chromosome of *E. coli* and many other bacteria [(55) and references therein]. The ShCAST differs from Tn7 in that the function of TnsD is performed by a Cas12K subunit using an RNA-directed interaction.

## Supplementary Material

gkz552_Supplemental_File
