# Supplementary material for: Targeted DNA transposition in vitro using a dCas9-transposase fusion protein
Source: Nucleic Acids Res. 2019 Jun 25;47(15):8126–35. doi: 10.1093/nar/gkz552 (PMC6735945; doi:10.1093/nar/gkz552)
Supplement: gkz552_Supplemental_File [file gkz552_supplemental_file.pdf]

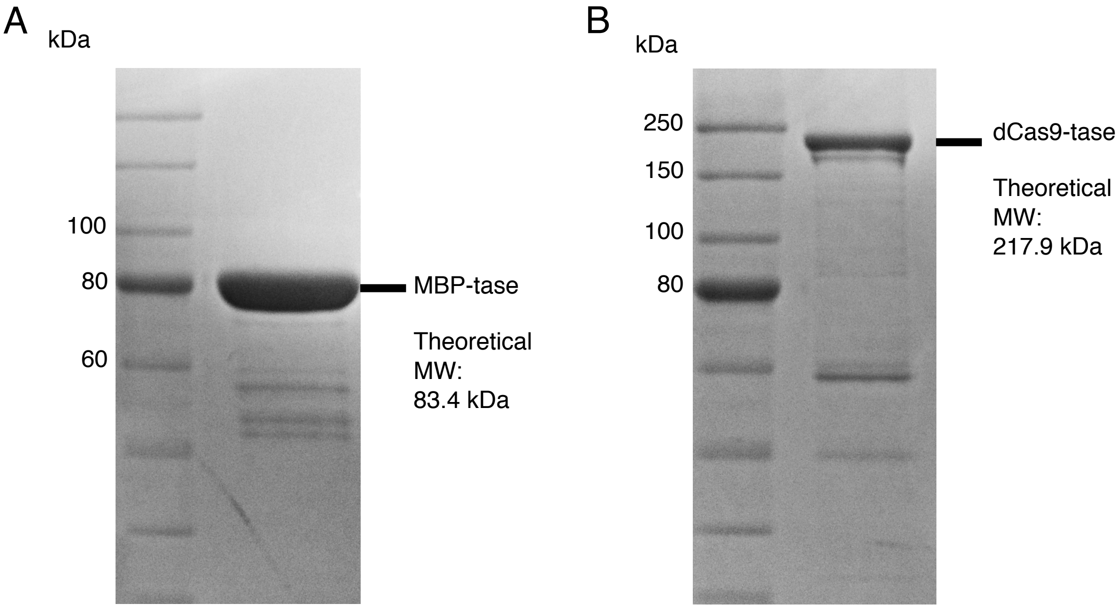

Supplementary Figure S1. Purification of MBP-Hsmar1 transposase and dCas9-transposase. Purified protein was analysed by SDS-PAGE to gauge protein purity. **A)** MBP-Hsmar1 transposase accounts for ~95% total protein. **B)** dCas9-transposase accounts for ~85% total protein.

Supplemental Table 1 Plasmid Sequences

pRC704

AATTCTATTAGGTTGGTGCAAAAGTAATTGCGGTTTTGGATCCGGCTGTAATCCG
GGCAGCGCAACGGAACATTCATCAGTGTAAAAATGGAATCAATAAAGCCCTGCG
CAGCGCGCAGGGTCAGCCTGAATACGCGTTTAATGACCAGCACAGTCGTGATG
GCAAGGTCAGAATAGCGCTGAGGTCTGCCTCGTGAAGAAGGTGTTGCTGACTC
ATACCAGGCCTGAATCGCCCCATCATCCAGCCAGAAAGTGAGGGAGCCACGGT
TGATGAGAGCTTTGTTGTAGGTGGACCAGTTGGTGATTTTGAACTTTTGCTTTGC
CACGGAACGGTCTGCGTTGTCGGGAAGATGCGTGATCTGATCCTTCAACTCAGC
AAAAGTTCGATTTATTCAACAAAGCCACGTTGTGTCTCAAAATCTCTGATGTTAC
ATTGCACAAGATAAAAATATATCATCATGAACAATAAAACTGTCTGCTTACATAAA
CAGTAATACAAGGGGTGTTATGAGCCATATTCAACGGGAAACGTCTTGCTCGAG
GCCGCGATTAAATTCCAACATGGATGCTGATTTATATGGGTATAAATGGGCTCG
CGATAATGTCGGGCAATCAGGTGCGACAATCTATCGATTGTATGGGAAGCCCGA
TGCGCCAGAGTTGTTTCTGAAACATGGCAAAGGTAGCGTTGCCAATGATGTTAC
AGATGAGATGGTCAGACTAAACTGGCTGACGGAATTTATGCCTCTTCCGACCAT
CAAGCATTTTATCCGTACTCCTGATGATGCATGGTTACTCACCACTGCGATCCCC
GGGAAAACAGCATTCCAGGTATTAGAAGAATATCCTGATTCAGGTGAAAATATTG
TTGATGCGCTGGCAGTGTTCCTGCGCCGGTTGCATTCGATTCCTGTTTGTAATT
GTCCTTTTAACAGCGATCGCGTATTTCGTCTCGCTCAGGCGCAATCACGAATGA
ATAACGGTTTGGTTGATGCGAGTGATTTTGATGACGAGCGTAATGGCTGGCCTG
TTGAACAAGTCTGGAAAGAAATGCATAAGCTTTTGCCATTCTCACCGGATTCAGT
CGTCACTCATGGTGATTTCTCACTTGATAACCTTATTTTTGACGAGGGGAAATTA
ATAGGTTGTATTGATGTTGGACGAGTCGGAATCGCAGACCGATACCAGGATCTT
GCCATCCTATGGAACTGCCTCGGTGAGTTTTCTCCTTCATTACAGAAACGGCTTT
TTCAAAAATATGGTATTGATAATCCTGATATGAATAAATTGCAGTTTCATTTGATG
CTCGATGAGTTTTTCTAATCAGAATTGGTTAATTGGTTGTAACACTGGCAGAGCA
TTACGCTGACTTGACGGGACGGCGGCTTTGTTGAATAAATCGAACTTTTGCTGA
GTTGAAGGATCAGATCACGCATCTTCCCGACAACGCAGACCGTTCCGTGGCAAA
GCAAAAGTTCAAAATCACCAACTGGTCCACCTACAACAAAGCTCTCATCAACCG
TGGCTCCCTCACTTTCTGGCTGGATGATGGGGCGATTCAGGCCTGGTATGAGTC
AGCAACACCTTCTTCACGAGGCAGACCTCAGCGCTATTCTGACCTTGCCATCAC
GACTGTGCTGGTCATTAAACGCGTATTCAGGCTGACCCTGCGCGCTGCGCAGG
GCTTTATTGATTCCATTTTTACACTGATGAATGTTCCGTTGCGCTGCCCGGATTA
CAGCCGGATCCGGCCACGATGCGTCCGGCGTAGAGGATCTGAAGATCAGCAGT
TCAACCTGTTGATAGTACGTACTAAGCTCTCATGTTTCACGTACTAAGCTCTCAT
GTTTAACGTACTAAGCTCTCATGTTTAACGAACTAAACCCTCATGGCTAACGTAC
TAAGCTCTCATGGCTAACGTACTAAGCTCTCATGTTTCACGTACTAAGCTCTCAT
GTTTGAACAATAAAATTAATATAAATCAGCAACTTAAATAGCCTCTAAGGTTTTAA
GTTTTATAAGAAAAAAAAGAATATATAAGGCTTTTAAAGCTTTTAAGGTTTAACGG
TTGTGGACAACAAGCCAGGGATGTAACGCACTGAGAAGCCCTTAGAGCCTCTCA
AAGCAATTTTGAGTGACACAGGAACACTTAACGGCTGACATGGGAATTCCACAT

GTGGAATTCCACATGTGGAATTGTGAGCGGATAACAATTTGTGGAATTCCCGGG
AGAGCTCGATATCGCATGCGGTACCTCTAGAAGAAGCTTGGGATCCAAAACCGC
AATTACTTTTGCACCAACCTAATAGAATTCTGGCGTAATAGCGAAGAGGCCCGC
ACCGATCGCCCTTCCCAACAGTTGCGCAGCCTGAATGGCGAATGGGACGCGCC
CTGTAGCGGCGCATTAAGCGCGGCGGGTGTGGTGGTTACGCGCAGCGTGACC
GCTACACTTGCCAGCGCCCTAGCGCCCGCTCCTTTCGCTTTCTTCCCTTCCTTT
CTCGCCACGTTCGCCGGCTTTCCCCGTCAAGCTCTAAATCGGGGGCTCCCTTTA
GGGTTCCGATTTAGTGCTTTACGGCACCTCGACCCCAAAAAACTTGATTAGGGT
GATGGTTCACGTAGTGGGCCATCGCCCTGATAGACGGTTTTTCGCCCTTTGACG
TTGGAGTCCACGTTCTTTAATAGTGGACTCTTGTTCCAAACTGGAACAACACTCA
ACCCTATCTCGGTCTATTCTTTTGATTTATAAGGGATTTTGCCGATTTCGGCCTAT
TGGTTAAAAAATGAGCTGATTTAACAAAAATTTAACGCGAATTTTAACAAAATATT
AACGCTTACAATTTAGGTGGCACTTTTCGGGGAAATGTGCGCGGAACCCCTATT
TGTTTATTTTTCTAAATACATTCAAATATGTATCCGCTCATGAGACAATAACCCTG
ATAAATGCTTCAATAATATTGAAAAAGGAAGAGTATGAGTATTCAACATTTCCGT
GTCGCCCTTATTCCCTTTTTTGCGGCATTTTGCCTTCCTGTTTTTGCTCACCCAG
AAACGCTGGTGAAAGTAAAAGATGCTGAAGATCAGTTGGGTGCACGAGTGGGTT
ACATCGAACTGGATCTCAACAGCGGTAAGATCCTTGAGAGTTTTCGCCCCGAAG
AACGAGCGGAAGAGCGCCCAATACGCAAACCGCCTCTCCCCGCGCGTTGGCCG
ATTCATTAATGCAG

pRC1104

TTCAGTTTAGCCTTAACCGGCGCATGACTTCAAGACTAACTCCTCTAAATCAATT
ACCAGTGGCTGCTGCCAGTGGTGCTTTTGCATGTCTTTCCGGGTTGGACTCAAG
ACGATAGTTACCGGATAAGGCGCAGCGGTCGGACTGAACGGGGGGTTCGTGCA
TACAGTCCAGCTTGGAGCGAACTGCCTACCCGGAACTGAGTGTCAGGCGTGGA
ATGAGACAAACGCGGCCATAACAGCGGAATGACACCGGTAAACCGAAAGGCAG
GAACAGGAGAGCGCACGAGGGAGCCGCCAGGGGGAAACGCCTGGTATCTTTAT
AGTCCTGTCGGGTTTCGCCACCACTGATTTGAGCGTCAGATTTCGTGATGCTTG
TCAGGGGGGCGGAGCCTATGGAAAAACGGCTTTGCCGCGGCCCTCTCACTTCC
CTGTTAAGTATCTTCCTGGCATCTTCCAGGAAATCTCCGCCCCGTTCGTAAGCC
ATTTCCGCTCGCCGCAGTCGAACGACCGAGCGTAGCGAGTCAGTGAGCGAGGA
AGCGGAATATATCCTGTATCACATATTCTGCTGACGCACCGGTGCAGCCTTTTTT
CTCCTGCCACATGAAGCACTTCACTGACACCCTCATCAGTGCCAACATAGTAAG
CCAGTATACACTCCGCTAGCGCTGATGTCCGGCGGTGCTTTTGCCGTTACGCAC
CACCCCGTCAGTAGCTGAACAGGAGGGACAGCTGATAGAAACAGAAGCCACTG
GAGCACCTCAAAAACACCATCATACACTAAATCAGTAAGTTGGCAGCATCACCC
GACGCACTTTGCGCCGAATAAATACCTGTGACGGAAGATCACTTCGCAGAATAA
ATAAATCCTGGTGTCCCTGTTGATACCGGGAAGCCCTGGGCCAACTTTTGGCGA
AAATGAGACGTTGATCGGCACGTAAGAGGTTCCAACTTTCACCATAATGAAATAA
GATCACTACCGGGCGTATTTTTTGAGTTATCGAGATTTTCAGGAGCTAAGGAAG
CTAAAATGGAGAAAAAAATCACTGGATATACCACCGTTGATATATCCCAATGGCA
TCGTAAAGAACATTTTGAGGCATTTCAGTCAGTTGCTCAATGTACCTATAACCAG

ACCGTTCAGCTGGATATTACGGCCTTTTTAAAGACCGTAAAGAAAAATAAGCACA
AGTTTTATCCGGCCTTTATTCACATTCTTGCCCGCCTGATGAATGCTCATCCGGA
ATTCCGTATGGCAATGAAAGACGGTGAGCTGGTGATATGGGATAGTGTTCACCC
TTGTTACACCGTTTTCCATGAGCAAACTGAAACGTTTTCATCGCTCTGGAGTGAA
TACCACGACGATTTCCGGCAGTTTCTACACATATATTCGCAAGATGTGGCGTGTT
ACGGTGAAAACCTGGCCTATTTCCCTAAAGGGTTTATTGAGAATATGTTTTTCGT
CTCAGCgAATCCCTGGGTGAGTTTCACCAGTTTTGATTTAAACGTGGCCAATATG
GACAACTTCTTCGCCCCCGTTTTCACCATGGGCAAATATTATACGCAAGGCGAC
AAGGTGCTGATGCCGCTGGCGATTCAGGTTCATCATGCCGTCTGTGATGGCTTC
CATGTCGGCAGAATGCTTAATGAATTACAACAGTACTGCGATGAGTGGCAGGGC
GGGGCGTAATTTTTTTAAGGCAGTTATTGGTGCCCTTAAACGCCTGGTGCTACG
CCTGAATAAGTGATAATAAGCGGATGAATGGCAGAAATTCGAAAGCAAATTCGA
CCCGGTCGTCGGTTCAGGGCAGGGTCGTTAAATAGCCGCTTATGTCTATTGCTG
GTTTACCGGTTTATTGACTACCGGAAGCAGTGTGACCGTGTGCTTCTCAAATGC
CTGAGGCCAGTTTGCTCAGGCTCTCCCCGTGGAGGTAATAATTGACGATATGAT
CATTTATTCTGCCTCCCAGAGCCTGATAAAAACGGTTAGCGCTTCGTTAATACAG
ATGTAGGTGTTCCACAGGGTAGCCAGCAGCATCCTGCGATGCAGATCCGGAAC
ATAATGGTGCAGGGCGCTTGTTTCGGCGTGGGTATGGTGGCAGGCCCCGTGGC
CGGGGGACTGTTGGGCGCTGCCGGCACCTGTCCTACGAGTTGCATGATAAAGA
AGACAGTCATAAGTGCGGCGACGATAGTCATGCCCCGCGCCCACCGGAAGGAG
CTACCGGACAGCGGTGCGGACTGTTGTAACTCAGAATAAGAAATGAGGCCGCT
CATGGCGTTGACTCTCAGTCATAGTATCGTGGTATCACCGGTTGGTTCCACTCT
CTGTTGCGGGCAACTTCAGCAGCACGTAGGGGACTTCCGCGTTTCCAGACTTTA
CGAAACACGGAAACCGAAGACCATTCATGTTGTTGCTCAGGTCGCAGACGTTTT
GCAGCAGCAGTCGCTTCACGTTCGCTCGCGTATCGGTGATTCATTCTGCTAACC
AGTAAGGCAACCCCGCCAGCCTAGCCGGGTCCTCAACGACAGGAGCACGATCA
TGCGCACCCGTGGCCAGGACCCAACGCTGCCCGAGATGCGCCGCGTGCGGCT
GCTGGAGATGGCGGACGCGATGGATATGTTCTGCCAAGGGTTGGTTTGCGCAT
TCACAGTTCTCCGCAAGAATTGATTGGCTCCAATTCTTGGAGTGGTGAATCCGTT
AGCGAGGTGCCGCCGGCTTCCATTCAGGTCGAGGTGGCCCGGCTCCATGCACC
GCGACGCAACGCGGGGAGGCAGACAAGGTATAGGGCGGCGCCTACAATCCAT
GCCAACCCGTTCCATGTGCTCGCCGAGGCGGCATAAATCGCCGTGACGATCAG
CGGTCCAGTGATCGAAGTTAGGCTGGTAAGAGCCGCGAGCGATCCTTGAAGCT
GTCCCTGATGGTCGTCATCTACCTGCCTGGACAGCATGGCCTGCAACGCGGGC
ATCCCGATGCCGCCGGAAGCGAGAAGAATCATAATGGGGAAGGCCATCCAGCC
TCGCGTCGCGAACGCCAGCAAGACGTAGCCCAGCGCGTCGGCCGCCATGCCG
GCGATAATGGCCTGCTTCTCGCCGAAACGTTTGGTGGCGGGACCAGTGACGAA
GGCTTGAGCGAGGGCGTGCAAGATTCCGAATACCGCAAGCGACAGGCCGATCA
TCGTCGCGCTCCAGCGAAAGCGGTCCTCGCCGAAAATGACCCAGAGCGCTGCC
GGCACCTGTCCTACGAGTTGCATGATAAAGAAGACAGTCATAAGTGCGGCGAC
GATAGTCATGCCCCGCGCCCACCGGAAGGAGCTGACTGGGTTGAAGGCTCTCA
AGGGCATCGGTCGACGCTCTCCCTTATGCGACTCCTGCATTAGGAAGCAGCCC
AGTAGTAGGTTGAGGCCGTTGAGCACCGCCGCCGCAAGGAATGGTGCATGCAA
GGAGATGGCGCCCAACAGTCCCCCGGCCACGGGGCCTGCCACCATACCCACG

CCGAAACAAGCGCTCATGAGCCCGAAGTGGCGAGCCCGATCTTCCCCATCGGT
GATGTCGGCGATATAGGCGCCAGCAACCGCACCTGTGGCGCCGGTGATGCCG
GCCACGATGCGTCCGGCGTAGAGGATCCACAGGACGGGTGTGGTCGCCATGAT
CGCGTAGTCGATAGTGGCTCCAAGTAGCGAAGCGAGCAGGACTGGGCGGCGG
CCAAAGCGGTCGGACAGTGCTCCGAGAACGGGTGCGCATAGAAATTGCATCAA
CGCATATAGCGCTAGCAGCACGCCATAGTGACTGGCGATGCTGTCGGAATGGA
CGATATCCCGCAAGAGGCCCGGCAGTACCGGCATAACCAAGCCTATGCCTACA
GCATCCAGGGTGACGGTGCCGAGGATGACGATGAGCGCATTGTTAGATTTCATA
CACGGTGCCTGACTGCGTTAGCAATTTAACTGTGATAAACTACCGCATTAAAGCT
TATCGATGATAAGCTGTCAAACATGAGAATTACAACTTATATCGTATGGGGCTGA
CTTCAGGTGCTACATTTGAAGAGATAAATTGCACTGAAATCTAGAAATATTTTATC
TGATTAATAAGATGATCTTCTTGAGATCGTTTTGGTCTGCGCGTAATCTCTTGCT
CTGAAAACGAAAAAACCGCCTTGCAGGGCGGTTTTTCGAAGGTTCTCTGAGCTA
CCAACTCTTTGAACCGAGGTAACTGGCTTGGAGGAGCGCAGTCACCAAAACTTG
TCCTTTCAGTTTAGCCTTAACCGGCGCATGACTTCAAGACTAACTCCTCTAAATC
AATTACCAGTGGCTGCTGCCAGTGGTGCTTTTGCATGTCTTTCCGGGTTGGACT
CAAGACGATAGTTACCGGATAAGGCGCAGCGGTCGGACTGAACGGGGGGTTCG
TGCATACAGTCCAGCTTGGAGCGAACTGCCTACCCGGAACTGAGTGTCAGGCG
TGGAATGAGACAAACGCGGCCATAACAGCGGAATGACACCGGTAAACCGAAAG
GCAGGAACAGGAGAGCGCACGAGGGAGCCGCCAGGGGGAAACGCCTGGTATC
TTTATAGTCCTGTCGGGTTTCGCCACCACTGATTTGAGCGTCAGATTTCGTGATG
CTTGTCAGGGGGGCGGAGCCTATGGAAAAACGGCTTTGCCGCGGCCCTCTCAC
TTCCCTGTTAAGTATCTTCCTGGCATCTTCCAGGAAATCTCCGCCCCGTTCGTAA
GCCATTTCCGCTCGCCGCAGTCGAACGACCGAGCGTAGCGAGTCAGTGAGCGA
GGAAGCGGAATATATCCTGTATCACATATTCTGCTGACGCACCGGTGCAGCCTT
TTTTCTCCTGCCACATGAAGCACTTCACTGACACCCTCATCAGTGCCAACATAGT
AAGCCAGTATACACTCCGCTAGCGCTGATGTCCGGCGGTGCTTTTGCCGTTACG
CACCACCCCGTCAGTAGCTGAACAGGAGGGACAGCTGATAGAAACAGAAGCCA
CTGGAGCACCTCAAAAACACCATCATACACTAAATCAGTAAGTTGGCAGCATCA
CCCGACGCACTTTGCGCCGAATAAATACCTGTGACGGAAGATCACTTCGCAGAA
TAAATAAATCCTGGTGTCCCTGTTGATACCGGGAAGCCCTGGGCCAACTTTTGG
CGAAAATGAGACGTTGATCGGCACGTAAGAGGTTCCAACTTTCACCATAATGAA
ATAAGATCACTACCGGGCGTATTTTTTGAGTTATCGAGATTTTCAGGAGCTAAGG
AAGCTAAAATGGAGAAAAAAATCACTGGATATACCACCGTTGATATATCCCAATG
GCATCGTAAAGAACATTTTGAGGCATTTCAGTCAGTTGCTCAATGTACCTATAAC
CAGACCGTTCAGCTGGATATTACGGCCTTTTTAAAGACCGTAAAGAAAAATAAG
CACAAGTTTTATCCGGCCTTTATTCACATTCTTGCCCGCCTGATGAATGCTCATC
CGGAATTCCGTATGGCAATGAAAGACGGTGAGCTGGTGATATGGGATAGTGTTC
ACCCTTGTTACACCGTTTTCCATGAGCAAACTGAAACGTTTTCATCGCTCTGGAG
TGAATACCACGACGATTTCCGGCAGTTTCTACACATATATTCGCAAGATGTGGC
GTGTTACGGTGAAAACCTGGCCTATTTCCCTAAAGGGTTTATTGAGAATATGTTT
TTCGTCTCAGCgAATCCCTGGGTGAGTTTCACCAGTTTTGATTTAAACGTGGCCA
ATATGGACAACTTCTTCGCCCCCGTTTTCACCATGGGCAAATATTATACGCAAGG
CGACAAGGTGCTGATGCCGCTGGCGATTCAGGTTCATCATGCCGTCTGTGATG

GCTTCCATGTCGGCAGAATGCTTAATGAATTACAACAGTACTGCGATGAGTGGC
AGGGCGGGGCGTAATTTTTTTAAGGCAGTTATTGGTGCCCTTAAACGCCTGGTG
CTACGCCTGAATAAGTGATAATAAGCGGATGAATGGCAGAAATTCGAAAGCAAA
TTCGACCCGGTCGTCGGTTCAGGGCAGGGTCGTTAAATAGCCGCTTATGTCTAT
TGCTGGTTTACCGGTTTATTGACTACCGGAAGCAGTGTGACCGTGTGCTTCTCA
AATGCCTGAGGCCAGTTTGCTCAGGCTCTCCCCGTGGAGGTAATAATTGACGAT
ATGATCATTTATTCTGCCTCCCAGAGCCTGATAAAAACGGTTAGCGCTTCGTTAA
TACAGATGTAGGTGTTCCACAGGGTAGCCAGCAGCATCCTGCGATGCAGATCC
GGAACATAATGGTGCAGGGCGCTTGTTTCGGCGTGGGTATGGTGGCAGGCCCC
GTGGCCGGGGGACTGTTGGGCGCTGCCGGCACCTGTCCTACGAGTTGCATGAT
AAAGAAGACAGTCATAAGTGCGGCGACGATAGTCATGCCCCGCGCCCACCGGA
AGGAGCTACCGGACAGCGGTGCGGACTGTTGTAACTCAGAATAAGAAATGAGG
CCGCTCATGGCGTTGACTCTCAGTCATAGTATCGTGGTATCACCGGTTGGTTCC
ACTCTCTGTTGCGGGCAACTTCAGCAGCACGTAGGGGACTTCCGCGTTTCCAGA
CTTTACGAAACACGGAAACCGAAGACCATTCATGTTGTTGCTCAGGTCGCAGAC
GTTTTGCAGCAGCAGTCGCTTCACGTTCGCTCGCGTATCGGTGATTCATTCTGC
TAACCAGTAAGGCAACCCCGCCAGCCTAGCCGGGTCCTCAACGACAGGAGCAC
GATCATGCGCACCCGTGGCCAGGACCCAACGCTGCCCGAGATGCGCCGCGTG
CGGCTGCTGGAGATGGCGGACGCGATGGATATGTTCTGCCAAGGGTTGGTTTG
CGCATTCACAGTTCTCCGCAAGAATTGATTGGCTCCAATTCTTGGAGTGGTGAA
TCCGTTAGCGAGGTGCCGCCGGCTTCCATTCAGGTCGAGGTGGCCCGGCTCCA
TGCACCGCGACGCAACGCGGGGAGGCAGACAAGGTATAGGGCGGCGCCTACA
ATCCATGCCAACCCGTTCCATGTGCTCGCCGAGGCGGCATAAATCGCCGTGAC
GATCAGCGGTCCAGTGATCGAAGTTAGGCTGGTAAGAGCCGCGAGCGATCCTT
GAAGCTGTCCCTGATGGTCGTCATCTACCTGCCTGGACAGCATGGCCTGCAAC
GCGGGCATCCCGATGCCGCCGGAAGCGAGAAGAATCATAATGGGGAAGGCCAT
CCAGCCTCGCGTCGCGAACGCCAGCAAGACGTAGCCCAGCGCGTCGGCCGCC
ATGCCGGCGATAATGGCCTGCTTCTCGCCGAAACGTTTGGTGGCGGGACCAGT
GACGAAGGCTTGAGCGAGGGCGTGCAAGATTCCGAATACCGCAAGCGACAGGC
CGATCATCGTCGCGCTCCAGCGAAAGCGGTCCTCGCCGAAAATGACCCAGAGC
GCTGCCGGCACCTGTCCTACGAGTTGCATGATAAAGAAGACAGTCATAAGTGCG
GCGACGATAGTCATGCCCCGCGCCCACCGGAAGGAGCTGACTGGGTTGAAGG
CTCTCAAGGGCATCGGTCGACGCTCTCCCTTATGCGACTCCTGCATTAGGAAGC
AGCCCAGTAGTAGGTTGAGGCCGTTGAGCACCGCCGCCGCAAGGAATGGTGCA
TGCAAGGAGATGGCGCCCAACAGTCCCCCGGCCACGGGGCCTGCCACCATAC
CCACGCCGAAACAAGCGCTCATGAGCCCGAAGTGGCGAGCCCGATCTTCCCCA
TCGGTGATGTCGGCGATATAGGCGCCAGCAACCGCACCTGTGGCGCCGGTGAT
GCCGGCCACGATGCGTCCGGCGTAGAGGATCCACAGGACGGGTGTGGTCGCC
ATGATCGCGTAGTCGATAGTGGCTCCAAGTAGCGAAGCGAGCAGGACTGGGCG
GCGGCCAAAGCGGTCGGACAGTGCTCCGAGAACGGGTGCGCATAGAAATTGCA
TCAACGCATATAGCGCTAGCAGCACGCCATAGTGACTGGCGATGCTGTCGGAAT
GGACGATATCCCGCAAGAGGCCCGGCAGTACCGGCATAACCAAGCCTATGCCT
ACAGCATCCAGGGTGACGGTGCCGAGGATGACGATGAGCGCATTGTTAGATTT
CATACACGGTGCCTGACTGCGTTAGCAATTTAACTGTGATAAACTACCGCATTAA

AGCTTATCGATGATAAGCTGTCAAACATGAGAATTACAACTTATATCGTATGGGG
CTGACTTCAGGTGCTACATTTGAAGAGATAAATTGCACTGAAATCTAGAAATATT
TTATCTGATTAATAAGATGATCTTCTTGAGATCGTTTTGGTCTGCGCGTAATCTCT
TGCTCTGAAAACGAAAAAACCGCCTTGCAGGGCGGTTTTTCGAAGGTTCTCTGA
GCTACCAACTCTTTGAACCGAGGTAACTGGCTTGGAGGAGCGCAGTCACCAAAA
CTTGTCCT

pRC2301

GAATTCCGGATGAGCATTCATCAGGCGGGCAAGAATGTGAATAAAGGCCGGAT
AAAACTTGTGCTTATTTTTCTTTACGGTCTTTAAAAAGGCCGTAATATCCAGCTGA
ACGGTCTGGTTATAGGTACATTGAGCAACTGACTGAAATGCCTCAAAATGTTCTT
TACGATGCCATTGGGATATATCAACGGTGGTATATCCAGTGATTTTTTTCTCCAT
TTTAGCTTCCTTAGCTCCTGAAAATCTCGATAACTCAAAAAATACGCCCGGTAGT
GATCTTATTTCATTATGGTGAAAGTTGGAACCTCTTACGTGCCGATCAACGTCTC
ATTTTCGCCAAAAGTTGGCCCAGGGCTTCCCGGTATCAACAGGGACACCAGGAT
TTATTTATTCTGCGAAGTGATCTTCCGTCACAGGTATTTATTCGGCGCAAAGTGC
GTCGGGTGATGCTGCCAACTTACTGATTTAGTGTATGATGGTGTTTTTGAGGTG
CTCCAGTGGCTTCTGTTTCTATCAGCTGTCCCTCCTGTTCAGCTACTGACGGGG
TGGTGCGTAACGGCAAAAGCACCGCCGGACATCAGCGCTAGCGGAGTGTATAC
TGGCTTACTATGTTGGCACTGATGAGGGTGTCAGTGAAGTGCTTCATGTGGCAG
GAGAAAAAAGGCTGCACCGGTGCGTCAGCAGAATATGTGATACAGGATATATTC
CGCTTCCTCGCTCACTGACTCGCTACGCTCGGTCGTTCGACTGCGGCGAGCGG
AAATGGCTTACGAACGGGGCGGAGATTTCCTGGAAGATGCCAGGAAGATACTTA
ACAGGGAAGTGAGAGGGCCGCGGCAAAGCCGTTTTTCCATAGGCTCCGCCCCC
CTGACAAGCATCACGAAATCTGACGCTCAAATCAGTGGTGGCGAAACCCGACA
GGACTATAAAGATACCAGGCGTTTCCCCCTGGCGGCTCCCTCGTGCGCTCTCCT
GTTCCTGCCTTTCGGTTTACCGGTGTCATTCCGCTGTTATGGCCGCGTTTGTCT
CATTCCACGCCTGACACTCAGTTCCGGGTAGGCAGTTCGCTCCAAGCTGGACT
GTATGCACGAACCCCCCGTTCAGTCCGACCGCTGCGCCTTATCCGGTAACTATC
GTCTTGAGTCCAACCCGGAAAGACATGCAAAAGCACCACTGGCAGCAGCCACT
GGTAATTGATTTAGAGGAGTTAGTCTTGAAGTCATGCGCCGGTTAAGGCTAAAC
TGAAAGGACAAGTTTTGGTGACTGCGCTCCTCCAAGCCAGTTACCTCGGTTCAA
AGAGTTGGTAGCTCAGAGAACCTTCGAAAAACCGCCCTGCAAGGCGGTTTTTTC
GTTTTCAGAGCAAGAGATTACGCGCAGACCAAAACGATCTCAAGAAGATCATCT
TATTAATCAGATAAAATATTTCTAGATTTCAGTGCAATTTATCTCTTCAAATGTAG
CACCTGAAGTCAGCCCCATACGATATAAGTTGTAATTCTCATGTTTGACAGCTTA
TCATCGATAAGCTTTAATGCGGTAGTTTATCACAGTTAAATTGCTAACGCAGTCA
GGCACCGTGTATGAAATCTAACAATGCGCTCATCGTCATCCTCGGCACCGTCAC
CCTGGATGCTGTAGGCATAGGCTTGGTTATGCCGGTACTGCCGGGCCTCTTGC
GGGATTACGAAATCATCCTGTGGAGCTTAGTAGGTTTAGCAAGATGGCAGCGCC
TAAATGTAGAATGATAAAAGGATTAAGAGATTAATTTCCCTAAAAATGATAAAACA
AGCGTTTTGAAAGCGCTTGTTTTTTTGGTTTGCAGTCAGAGTAGAATAGAAGTAT
CAAAAAAAGCACCGACTCGGTGCCACTTTTTCAAGTTGATAACGGACTAGCCTT

ATTTTAACTTGCTATGCTGTTTTGAATGGTTCCAACAAGATTATTTTATAACTTTTA
TAACAAATAATCAAGGAGAAATTCAAAGAAATTTATCAGCCATAAAACAATACTTA
ATACTATAGAATGATAACAAAATAAACTACTTTTTAAAAGAATTTTGTGTTATAATC
TATTTATTATTAAGTATTGGGTAATATTTTTTGAAGAGATATTTTGAAAAAGAAAAA
TTAAAGCATATTAAACTAATTTCGGAGGTCATTAAAACTATTATTGAAATCATCAA
ACTCATTATGGATTTAATTTAAACTTTTTATTTTAGGAGGCAAAAATGGATAAGAA
ATACTCAATAGGCTTAGCTATCGGCACAAATAGCGTCGGATGGGCGGTGATCAC
TGATGAATATAAGGTTCCGTCTAAAAAGTTCAAGGTTCTGGGAAATACAGACCG
CCACAGTATCAAAAAAAATCTTATAGGGGCTCTTTTATTTGACAGTGGAGAGACA
GCGGAAGCGACTCGTCTCAAACGGACAGCTCGTAGAAGGTATACACGTCGGAA
GAATCGTATTTGTTATCTACAGGAGATTTTTTCAAATGAGATGGCGAAAGTAGAT
GATAGTTTCTTTCATCGACTTGAAGAGTCTTTTTTGGTGGAAGAAGACAAGAAGC
ATGAACGTCATCCTATTTTTGGAAATATAGTAGATGAAGTTGCTTATCATGAGAA
ATATCCAACTATCTATCATCTGCGAAAAAAATTGGTAGATTCTACTGATAAAGCG
GATTTGCGCTTAATCTATTTGGCCTTAGCGCATATGATTAAGTTTCGTGGTCATT
TTTTGATTGAGGGAGATTTAAATCCTGATAATAGTGATGTGGACAAACTATTTAT
CCAGTTGGTACAAACCTACAATCAATTATTTGAAGAAAACCCTATTAACGCAAGT
GGAGTAGATGCTAAAGCGATTCTTTCTGCACGATTGAGTAAATCAAGACGATTA
GAAAATCTCATTGCTCAGCTCCCCGGTGAGAAGAAAAATGGCTTATTTGGGAAT
CTCATTGCTTTGTCATTGGGTTTGACCCCTAATTTTAAATCAAATTTTGATTTGGC
AGAAGATGCTAAATTACAGCTTTCAAAAGATACTTACGATGATGATTTAGATAATT
TATTGGCGCAAATTGGAGATCAATATGCTGATTTGTTTTTGGCAGCTAAGAATTT
ATCAGATGCTATTTTACTTTCAGATATCCTAAGAGTAAATACTGAAATAACTAAGG
CTCCCCTATCAGCTTCAATGATTAAACGCTACGATGAACATCATCAAGACTTGAC
TCTTTTAAAAGCTTTAGTTCGACAACAACTTCCAGAAAAGTATAAAGAAATCTTTT
TTGATCAATCAAAAAACGGATATGCAGGTTATATTGATGGGGGAGCTAGCCAAG
AAGAATTTTATAAATTTATCAAACCAATTTTAGAAAAAATGGATGGTACTGAGGAA
TTATTGGTGAAACTAAATCGTGAAGATTTGCTGCGCAAGCAACGGACCTTTGAC
AACGGCTCTATTCCCCATCAAATTCACTTGGGTGAGCTGCATGCTATTTTGAGAA
GACAAGAAGACTTTTATCCATTTTTAAAAGACAATCGTGAGAAGATTGAAAAAAT
CTTGACTTTTCGAATTCCTTATTATGTTGGTCCATTGGCGCGTGGCAATAGTCGT
TTTGCATGGATGACTCGGAAGTCTGAAGAAACAATTACCCCATGGAATTTTGAAG
AAGTTGTCGATAAAGGTGCTTCAGCTCAATCATTTATTGAACGCATGACAAACTT
TGATAAAAATCTTCCAAATGAAAAAGTACTACCAAAACATAGTTTGCTTTATGAGT
ATTTTACGGTTTATAACGAATTGACAAAGGTCAAATATGTTACTGAAGGAATGCG
AAAACCAGCATTTCTTTCAGGTGAACAGAAGAAAGCCATTGTTGATTTACTCTTC
AAAACAAATCGAAAAGTAACCGTTAAGCAATTAAAAGAAGATTATTTCAAAAAAAT
AGAATGTTTTGATAGTGTTGAAATTTCAGGAGTTGAAGATAGATTTAATGCTTCAT
TAGGTACCTACCATGATTTGCTAAAAATTATTAAAGATAAAGATTTTTTGGATAAT
GAAGAAAATGAAGATATCTTAGAGGATATTGTTTTAACATTGACCTTATTTGAAGA
TAGGGAGATGATTGAGGAAAGACTTAAAACATATGCTCACCTCTTTGATGATAAG
GTGATGAAACAGCTTAAACGTCGCCGTTATACTGGTTGGGGACGTTTGTCTCGA
AAATTGATTAATGGTATTAGGGATAAGCAATCTGGCAAAACAATATTAGATTTTTT
GAAATCAGATGGTTTTGCCAATCGCAATTTTATGCAGCTGATCCATGATGATAGT

TTGACATTTAAAGAAGACATTCAAAAAGCACAAGTGTCTGGACAAGGCGATAGTT
TACATGAACATATTGCAAATTTAGCTGGTAGCCCTGCTATTAAAAAAGGTATTTTA
CAGACTGTAAAAGTTGTTGATGAATTGGTCAAAGTAATGGGGCGGCATAAGCCA
GAAAATATCGTTATTGAAATGGCACGTGAAAATCAGACAACTCAAAAGGGCCAG
AAAAATTCGCGAGAGCGTATGAAACGAATCGAAGAAGGTATCAAAGAATTAGGA
AGTCAGATTCTTAAAGAGCATCCTGTTGAAAATACTCAATTGCAAAATGAAAAGC
TCTATCTCTATTATCTCCAAAATGGAAGAGACATGTATGTGGACCAAGAATTAGA
TATTAATCGTTTAAGTGATTATGATGTCGATGCCATTGTTCCACAAAGTTTCCTTA
AAGACGATTCAATAGACAATAAGGTCTTAACGCGTTCTGATAAAAATCGTGGTAA
ATCGGATAACGTTCCAAGTGAAGAAGTAGTCAAAAAGATGAAAAACTATTGGAG
ACAACTTCTAAACGCCAAGTTAATCACTCAACGTAAGTTTGATAATTTAACGAAA
GCTGAACGTGGAGGTTTGAGTGAACTTGATAAAGCTGGTTTTATCAAACGCCAA
TTGGTTGAAACTCGCCAAATCACTAAGCATGTGGCACAAATTTTGGATAGTCGCA
TGAATACTAAATACGATGAAAATGATAAACTTATTCGAGAGGTTAAAGTGATTAC
CTTAAAATCTAAATTAGTTTCTGACTTCCGAAAAGATTTCCAATTCTATAAAGTAC
GTGAGATTAACAATTACCATCATGCCCATGATGCGTATCTAAATGCCGTCGTTGG
AACTGCTTTGATTAAGAAATATCCAAAACTTGAATCGGAGTTTGTCTATGGTGAT
TATAAAGTTTATGATGTTCGTAAAATGATTGCTAAGTCTGAGCAAGAAATAGGCA
AAGCAACCGCAAAATATTTCTTTTACTCTAATATCATGAACTTCTTCAAAACAGAA
ATTACACTTGCAAATGGAGAGATTCGCAAACGCCCTCTAATCGAAACTAATGGG
GAAACTGGAGAAATTGTCTGGGATAAAGGGCGAGATTTTGCCACAGTGCGCAAA
GTATTGTCCATGCCCCAAGTCAATATTGTCAAGAAAACAGAAGTACAGACAGGC
GGATTCTCCAAGGAGTCAATTTTACCAAAAAGAAATTCGGACAAGCTTATTGCTC
GTAAAAAAGACTGGGATCCAAAAAAATATGGTGGTTTTGATAGTCCAACGGTAG
CTTATTCAGTCCTAGTGGTTGCTAAGGTGGAAAAAGGGAAATCGAAGAAGTTAA
AATCCGTTAAAGAGTTACTAGGGATCACAATTATGGAAAGAAGTTCCTTTGAAAA
AAATCCGATTGACTTTTTAGAAGCTAAAGGATATAAGGAAGTTAAAAAAGACTTA
ATCATTAAACTACCTAAATATAGTCTTTTTGAGTTAGAAAACGGTCGTAAACGGA
TGCTGGCTAGTGCCGGAGAATTACAAAAAGGAAATGAGCTGGCTCTGCCAAGC
AAATATGTGAATTTTTTATATTTAGCTAGTCATTATGAAAAGTTGAAGGGTAGTCC
AGAAGATAACGAACAAAAACAATTGTTTGTGGAGCAGCATAAGCATTATTTAGAT
GAGATTATTGAGCAAATCAGTGAATTTTCTAAGCGTGTTATTTTAGCAGATGCCA
ATTTAGATAAAGTTCTTAGTGCATATAACAAACATAGAGACAAACCAATACGTGA
ACAAGCAGAAAATATTATTCATTTATTTACGTTGACGAATCTTGGAGCTCCCGCT
GCTTTTAAATATTTTGATACAACAATTGATCGTAAACGATATACGTCTACAAAAGA
AGTTTTAGATGCCACTCTTATCCATCAATCCATCACTGGTCTTTATGAAACACGC
ATTGATTTGAGTCAGCTAGGAGGTGACTGAAGTATATTTTAGATGAAGATTATTT
CTTAATAACTAAAAATATGGTATAATACTCTTAATAAATGCAGTAATACAGGGGCT
TTTCAAGACTGAAGTCTAGCTGAGACAAATAGTGCGATTACGAAATTTTTTAGAC
AAAAATAGTCTACGAGGTTTTAGAGCTATGCTGTTTTGAATGGTCCCAAAACTGA
GACCAGTCTCGGAAGCTCAAAGGTCTCGTTTTAGAGCTATGCTGTTTTGAATGG
TCCCAAAACTTCAGCACACTGAGACTTGTTGAGTTCCATGTTTTAGAGCTATGCT
GTTTTGAATGGACTCCATTCAACATTGCCGATGATAACTTGAGAAAGAGGGTTAA
TACCAGCAGTCGGATACCTTCCTATTCTTTCTGTTAAAGCGTTTTCATGTTATAAT

AGGCAAAAGAAGAGTAGTGTGATCGTCCATTCCGACAGCATCGCCAGTCACTAT
GGCGTGCTGCTAGCGCTATATGCGTTGATGCAATTTCTATGCGCACCCGTTCTC
GGAGCACTGTCCGACCGCTTTGGCCGCCGCCCAGTCCTGCTCGCTTCGCTACT
TGGAGCCACTATCGACTACGCGATCATGGCGACCACACCCGTCCTGTGGATCC
TCTACGCCGGACGCATCGTGGCCGGCATCACCGGCGCCACAGGTGCGGTTGCT
GGCGCCTATATCGCCGACATCACCGATGGGGAAGATCGGGCTCGCCACTTCGG
GCTCATGAGCGCTTGTTTCGGCGTGGGTATGGTGGCAGGCCCCGTGGCCGGG
GGACTGTTGGGCGCCATCTCCTTGCATGCACCATTCCTTGCGGCGGCGGTGCT
CAACGGCCTCAACCTACTACTGGGCTGCTTCCTAATGCAGGAGTCGCATAAGGG
AGAGCGTCGACCGATGCCCTTGAGAGCCTTCAACCCAGTCAGCTCCTTCCGGT
GGGCGCGGGGCATGACTATCGTCGCCGCACTTATGACTGTCTTCTTTATCATGC
AACTCGTAGGACAGGTGCCGGCAGCGCTCTGGGTCATTTTCGGCGAGGACCGC
TTTCGCTGGAGCGCGACGATGATCGGCCTGTCGCTTGCGGTATTCGGAATCTTG
CACGCCCTCGCTCAAGCCTTCGTCACTGGTCCCGCCACCAAACGTTTCGGCGA
GAAGCAGGCCATTATCGCCGGCATGGCGGCCGACGCGCTGGGCTACGTCTTGC
TGGCGTTCGCGACGCGAGGCTGGATGGCCTTCCCCATTATGATTCTTCTCGCTT
CCGGCGGCATCGGGATGCCCGCGTTGCAGGCCATGCTGTCCAGGCAGGTAGA
TGACGACCATCAGGGACAGCTTCAAGGATCGCTCGCGGCTCTTACCAGCCTAA
CTTCGATCATTGGACCGCTGATCGTCACGGCGATTTATGCCGCCTCGGCGAGC
ACATGGAACGGGTTGGCATGGATTGTAGGCGCCGCCCTATACCTTGTCTGCCTC
CCCGCGTTGCGTCGCGGTGCATGGAGCCGGGCCACCTCGACCTGAATGGAAG
CCGGCGGCACCTCGCTAACGGATTCACCACTCCAAGAATTGGAGCCAATCAATT
CTTGCGGAGAACTGTGAATGCGCAAACCAACCCTTGGCAGAACATATCCATCGC
GTCCGCCATCTCCAGCAGCCGCACGCGGCGCATCTCGGGCAGCGTTGGGTCCT
GGCCACGGGTGCGCATGATCGTGCTCCTGTCGTTGAGGACCCGGCTAGGCTGG
CGGGGTTGCCTTACTGGTTAGCAGAATGAATCACCGATACGCGAGCGAACGTG
AAGCGACTGCTGCTGCAAAACGTCTGCGACCTGAGCAACAACATGAATGGTCTT
CGGTTTCCGTGTTTCGTAAAGTCTGGAAACGCGGAAGTCCCCTACGTGCTGCTG
AAGTTGCCCGCAACAGAGAGTGGAACCAACCGGTGATACCACGATACTATGACT
GAGAGTCAACGCCATGAGCGGCCTCATTTCTTATTCTGAGTTACAACAGTCCGC
ACCGCTGTCCGGTAGCTCCTTCCGGTGGGCGCGGGGCATGACTATCGTCGCCG
CACTTATGACTGTCTTCTTTATCATGCAACTCGTAGGACAGGTGCCGGCAGCGC
CCAACAGTCCCCCGGCCACGGGGCCTGCCACCATACCCACGCCGAAACAAGC
GCCCTGCACCATTATGTTCCGGATCTGCATCGCAGGATGCTGCTGGCTACCCTG
TGGAACACCTACATCTGTATTAACGAAGCGCTAACCGTTTTTATCAGGCTCTGG
GAGGCAGAATAAATGATCATATCGTCAATTATTACCTCCACGGGGAGAGCCTGA
GCAAACTGGCCTCAGGCATTTGAGAAGCACACGGTCACACTGCTTCCGGTAGTC
AATAAACCGGTAAACCAGCAATAGACATAAGCGGCTATTTAACGACCCTGCCCT
GAACCGACGACCGGGTCGAATTTGCTTTCGAATTTCTGCCATTCATCCGCTTATT
ATCACTTATTCAGGCGTAGCACCAGGCGTTTAAGGGCACCAATAACTGCCTTAA
AAAAATTACGCCCCGCCCTGCCACTCATCGCAGTACTGTTGTAATTCATTAAGCA
TTCTGCCGACATGGAAGCCATCACAGACGGCATGATGAACCTGAATCGCCAGC
GGCATCAGCACCTTGTCGCCTTGCGTATAATATTTGCCCATGGTGAAAACGGGG
GCGAAGAAGTTGTCCATATTGGCCACGTTTAAATCAAAACTGGTGAAACTCACC

CAGGGATTGGCTGAGACGAAAAACATATTCTCAATAAACCCTTTAGGGAAATAG
GCCAGGTTTTCACCGTAACACGCCACATCTTGCGAATATATGTGTAGAAACTGC
CGGAAATCGTCGTGGTATTCACTCCAGAGCGATGAAAACGTTTCAGTTTGCTCA
TGGAAAACGGTGTAACAAGGGTGAACACTATCCCATATCACCAGCTCACCGTCT
TTCATTGCCATACG

pRC2302

GAATTCCGGATGAGCATTCATCAGGCGGGCAAGAATGTGAATAAAGGCCGGAT
AAAACTTGTGCTTATTTTTCTTTACGGTCTTTAAAAAGGCCGTAATATCCAGCTGA
ACGGTCTGGTTATAGGTACATTGAGCAACTGACTGAAATGCCTCAAAATGTTCTT
TACGATGCCATTGGGATATATCAACGGTGGTATATCCAGTGATTTTTTTCTCCAT
TTTAGCTTCCTTAGCTCCTGAAAATCTCGATAACTCAAAAAATACGCCCGGTAGT
GATCTTATTTCATTATGGTGAAAGTTGGAACCTCTTACGTGCCGATCAACGTCTC
ATTTTCGCCAAAAGTTGGCCCAGGGCTTCCCGGTATCAACAGGGACACCAGGAT
TTATTTATTCTGCGAAGTGATCTTCCGTCACAGGTATTTATTCGGCGCAAAGTGC
GTCGGGTGATGCTGCCAACTTACTGATTTAGTGTATGATGGTGTTTTTGAGGTG
CTCCAGTGGCTTCTGTTTCTATCAGCTGTCCCTCCTGTTCAGCTACTGACGGGG
TGGTGCGTAACGGCAAAAGCACCGCCGGACATCAGCGCTAGCGGAGTGTATAC
TGGCTTACTATGTTGGCACTGATGAGGGTGTCAGTGAAGTGCTTCATGTGGCAG
GAGAAAAAAGGCTGCACCGGTGCGTCAGCAGAATATGTGATACAGGATATATTC
CGCTTCCTCGCTCACTGACTCGCTACGCTCGGTCGTTCGACTGCGGCGAGCGG
AAATGGCTTACGAACGGGGCGGAGATTTCCTGGAAGATGCCAGGAAGATACTTA
ACAGGGAAGTGAGAGGGCCGCGGCAAAGCCGTTTTTCCATAGGCTCCGCCCCC
CTGACAAGCATCACGAAATCTGACGCTCAAATCAGTGGTGGCGAAACCCGACA
GGACTATAAAGATACCAGGCGTTTCCCCCTGGCGGCTCCCTCGTGCGCTCTCCT
GTTCCTGCCTTTCGGTTTACCGGTGTCATTCCGCTGTTATGGCCGCGTTTGTCT
CATTCCACGCCTGACACTCAGTTCCGGGTAGGCAGTTCGCTCCAAGCTGGACT
GTATGCACGAACCCCCCGTTCAGTCCGACCGCTGCGCCTTATCCGGTAACTATC
GTCTTGAGTCCAACCCGGAAAGACATGCAAAAGCACCACTGGCAGCAGCCACT
GGTAATTGATTTAGAGGAGTTAGTCTTGAAGTCATGCGCCGGTTAAGGCTAAAC
TGAAAGGACAAGTTTTGGTGACTGCGCTCCTCCAAGCCAGTTACCTCGGTTCAA
AGAGTTGGTAGCTCAGAGAACCTTCGAAAAACCGCCCTGCAAGGCGGTTTTTTC
GTTTTCAGAGCAAGAGATTACGCGCAGACCAAAACGATCTCAAGAAGATCATCT
TATTAATCAGATAAAATATTTCTAGATTTCAGTGCAATTTATCTCTTCAAATGTAG
CACCTGAAGTCAGCCCCATACGATATAAGTTGTAATTCTCATGTTTGACAGCTTA
TCATCGATAAGCTTTAATGCGGTAGTTTATCACAGTTAAATTGCTAACGCAGTCA
GGCACCGTGTATGAAATCTAACAATGCGCTCATCGTCATCCTCGGCACCGTCAC
CCTGGATGCTGTAGGCATAGGCTTGGTTATGCCGGTACTGCCGGGCCTCTTGC
GGGATTACGAAATCATCCTGTGGAGCTTAGTAGGTTTAGCAAGATGGCAGCGCC
TAAATGTAGAATGATAAAAGGATTAAGAGATTAATTTCCCTAAAAATGATAAAACA
AGCGTTTTGAAAGCGCTTGTTTTTTTGGTTTGCAGTCAGAGTAGAATAGAAGTAT
CAAAAAAAGCACCGACTCGGTGCCACTTTTTCAAGTTGATAACGGACTAGCCTT
ATTTTAACTTGCTATGCTGTTTTGAATGGTTCCAACAAGATTATTTTATAACTTTTA

TAACAAATAATCAAGGAGAAATTCAAAGAAATTTATCAGCCATAAAACAATACTTA
ATACTATAGAATGATAACAAAATAAACTACTTTTTAAAAGAATTTTGTGTTATAATC
TATTTATTATTAAGTATTGGGTAATATTTTTTGAAGAGATATTTTGAAAAAGAAAAA
TTAAAGCATATTAAACTAATTTCGGAGGTCATTAAAACTATTATTGAAATCATCAA
ACTCATGCATCATATGGAAATGATGCTCGATAAGAAACAGATTCGTGCGATCTTT
CTCTTTGAGTTTAAAATGGGTCGCAAAGCGGCGGAGACGACGCGTAATATTAAC
AACGCGTTCGGTCCTGGCACCGCGAACGAGCGTACCGTGCAATGGTGGTTCAA
AAAGTTTCGCAAAGGCGACGAATCTCTGGAGGACGAAGAGCGTTCTGGCCGCC
CGTCCGAGGTTGACAACGACCAGCTGCGTGCAATCATCGAAGCTGATCCGCTG
ACTACCACCCGCGAAGTTGCTGAAGAACTGAATGTGGATCACTCTACTGTGGTT
CGCCACCTGAAACAGATCGGTAAAGTAAAAAAACTGGACAAATGGGTTCCTCAT
GAACTGTCTGAAAACCAGAAAAACCGTCGTTTCGAAGTTAGCTCCTCTCTGATTC
TGCGTAACAACAACGAACCGTTCCTGGATCGTATCGTAACCTGTGATGAGAAAT
GGATTCTGTATGATAACCGTCGCCGCTCTGCTCAGTGGCTGGATCGCGAAGAA
GCTCCAAAACACTTCCCGAAACCGAATCTGCACCAGAAGAAAGTCATGGTAACC
GTATGGTGGTCTGCCGCAGGTGTTATCCACTATTCCTTCCTGAACCCGGGCGAA
ACTATCACCAGCGAAAAATACTGCCAGCAGATTGACGAAATGCACCGTAAACTG
CAGCGTCTGCAGCCAGCACTGGTGAATCGTAAAGGTCCGATCCTGCTGCATGAT
AACGCCCGTCCGCACGTTGCCCAACCGACCCTGCAGAAACTGAACGAACTGGG
CTATGAAGTTCTGCCACACCCGCCGTACTCCCCGGATCTGTCCCCGACTGACTA
CCATTTCTTCAAGCATCTGGACAACTTCCTGCAGGGTAAACGTTTTCACAACCAA
CAGGACGCAGAAAACGCTTTCCAGGAGTTCGTCGAAAGCCGTTCCACTGACTTC
TACGCGACCGGTATCAACAAGCTGATCAGCCGTTGGCAGAAATGCGTGGACTG
TAACGGCAGCTACTTCGATtctagaGGAGGTGGCTCAGAAGGTGGCGGATCTGAA
GGTGGCTCTGGAactagtATGAGCGATAAAATTATTCACCTGACTGACGACAGTTT
TGACACGGATGTACTCAAAGCGGACGGGGCGATCCTCGTCGATTTCTGGGCAG
AGTGGTGCGGTCCGTGCAAAATGATCGCCCCGATTCTGGATGAAATCGCTGAC
GAATATCAGGGCAAACTGACCGTTGCAAAACTGAACATCGATCAAAACCCTGGC
ACTGCGCCGAAATATGGCATCCGTGGTATCCCGACTCTGCTGCTGTTCAAAAAC
GGTGAAGTGGCGGCAACCAAAGTGGGTGCACTGTCTAAAGGTCAGTTGAAAGA
GTTCCTCGACGCTAACCTGGCCGGTTCTGGTTCTGGCCATATGCACCATCATCA
TCATCATTCTTCTGGTCTGGTGCCACGCGGTTCTGGTATGAAAGAAACCGCTGC
TGCTAAATTCGAACGCCAGCACATGGACAGCCCAGATCTGGGTACCGACGACG
ACGACAAGGCCATGGCTGATATCCTCGAGATGGATAAGAAATACTCAATAGGCT
TAGCTATCGGCACAAATAGCGTCGGATGGGCGGTGATCACTGATGAATATAAGG
TTCCGTCTAAAAAGTTCAAGGTTCTGGGAAATACAGACCGCCACAGTATCAAAA
AAAATCTTATAGGGGCTCTTTTATTTGACAGTGGAGAGACAGCGGAAGCGACTC
GTCTCAAACGGACAGCTCGTAGAAGGTATACACGTCGGAAGAATCGTATTTGTT
ATCTACAGGAGATTTTTTCAAATGAGATGGCGAAAGTAGATGATAGTTTCTTTCA
TCGACTTGAAGAGTCTTTTTTGGTGGAAGAAGACAAGAAGCATGAACGTCATCC
TATTTTTGGAAATATAGTAGATGAAGTTGCTTATCATGAGAAATATCCAACTATCT
ATCATCTGCGAAAAAAATTGGTAGATTCTACTGATAAAGCGGATTTGCGCTTAAT
CTATTTGGCCTTAGCGCATATGATTAAGTTTCGTGGTCATTTTTTGATTGAGGGA
GATTTAAATCCTGATAATAGTGATGTGGACAAACTATTTATCCAGTTGGTACAAA

CCTACAATCAATTATTTGAAGAAAACCCTATTAACGCAAGTGGAGTAGATGCTAA
AGCGATTCTTTCTGCACGATTGAGTAAATCAAGACGATTAGAAAATCTCATTGCT
CAGCTCCCCGGTGAGAAGAAAAATGGCTTATTTGGGAATCTCATTGCTTTGTCAT
TGGGTTTGACCCCTAATTTTAAATCAAATTTTGATTTGGCAGAAGATGCTAAATTA
CAGCTTTCAAAAGATACTTACGATGATGATTTAGATAATTTATTGGCGCAAATTG
GAGATCAATATGCTGATTTGTTTTTGGCAGCTAAGAATTTATCAGATGCTATTTTA
CTTTCAGATATCCTAAGAGTAAATACTGAAATAACTAAGGCTCCCCTATCAGCTT
CAATGATTAAACGCTACGATGAACATCATCAAGACTTGACTCTTTTAAAAGCTTT
AGTTCGACAACAACTTCCAGAAAAGTATAAAGAAATCTTTTTTGATCAATCAAAAA
ACGGATATGCAGGTTATATTGATGGGGGAGCTAGCCAAGAAGAATTTTATAAATT
TATCAAACCAATTTTAGAAAAAATGGATGGTACTGAGGAATTATTGGTGAAACTA
AATCGTGAAGATTTGCTGCGCAAGCAACGGACCTTTGACAACGGCTCTATTCCC
CATCAAATTCACTTGGGTGAGCTGCATGCTATTTTGAGAAGACAAGAAGACTTTT
ATCCATTTTTAAAAGACAATCGTGAGAAGATTGAAAAAATCTTGACTTTTCGAATT
CCTTATTATGTTGGTCCATTGGCGCGTGGCAATAGTCGTTTTGCATGGATGACT
CGGAAGTCTGAAGAAACAATTACCCCATGGAATTTTGAAGAAGTTGTCGATAAA
GGTGCTTCAGCTCAATCATTTATTGAACGCATGACAAACTTTGATAAAAATCTTC
CAAATGAAAAAGTACTACCAAAACATAGTTTGCTTTATGAGTATTTTACGGTTTAT
AACGAATTGACAAAGGTCAAATATGTTACTGAAGGAATGCGAAAACCAGCATTTC
TTTCAGGTGAACAGAAGAAAGCCATTGTTGATTTACTCTTCAAAACAAATCGAAA
AGTAACCGTTAAGCAATTAAAAGAAGATTATTTCAAAAAAATAGAATGTTTTGATA
GTGTTGAAATTTCAGGAGTTGAAGATAGATTTAATGCTTCATTAGGTACCTACCA
TGATTTGCTAAAAATTATTAAAGATAAAGATTTTTTGGATAATGAAGAAAATGAAG
ATATCTTAGAGGATATTGTTTTAACATTGACCTTATTTGAAGATAGGGAGATGATT
GAGGAAAGACTTAAAACATATGCTCACCTCTTTGATGATAAGGTGATGAAACAG
CTTAAACGTCGCCGTTATACTGGTTGGGGACGTTTGTCTCGAAAATTGATTAATG
GTATTAGGGATAAGCAATCTGGCAAAACAATATTAGATTTTTTGAAATCAGATGG
TTTTGCCAATCGCAATTTTATGCAGCTGATCCATGATGATAGTTTGACATTTAAA
GAAGACATTCAAAAAGCACAAGTGTCTGGACAAGGCGATAGTTTACATGAACAT
ATTGCAAATTTAGCTGGTAGCCCTGCTATTAAAAAAGGTATTTTACAGACTGTAA
AAGTTGTTGATGAATTGGTCAAAGTAATGGGGCGGCATAAGCCAGAAAATATCG
TTATTGAAATGGCACGTGAAAATCAGACAACTCAAAAGGGCCAGAAAAATTCGC
GAGAGCGTATGAAACGAATCGAAGAAGGTATCAAAGAATTAGGAAGTCAGATTC
TTAAAGAGCATCCTGTTGAAAATACTCAATTGCAAAATGAAAAGCTCTATCTCTAT
TATCTCCAAAATGGAAGAGACATGTATGTGGACCAAGAATTAGATATTAATCGTT
TAAGTGATTATGATGTCGATGCCATTGTTCCACAAAGTTTCCTTAAAGACGATTC
AATAGACAATAAGGTCTTAACGCGTTCTGATAAAAATCGTGGTAAATCGGATAAC
GTTCCAAGTGAAGAAGTAGTCAAAAAGATGAAAAACTATTGGAGACAACTTCTAA
ACGCCAAGTTAATCACTCAACGTAAGTTTGATAATTTAACGAAAGCTGAACGTGG
AGGTTTGAGTGAACTTGATAAAGCTGGTTTTATCAAACGCCAATTGGTTGAAACT
CGCCAAATCACTAAGCATGTGGCACAAATTTTGGATAGTCGCATGAATACTAAAT
ACGATGAAAATGATAAACTTATTCGAGAGGTTAAAGTGATTACCTTAAAATCTAA
ATTAGTTTCTGACTTCCGAAAAGATTTCCAATTCTATAAAGTACGTGAGATTAACA
ATTACCATCATGCCCATGATGCGTATCTAAATGCCGTCGTTGGAACTGCTTTGAT

TAAGAAATATCCAAAACTTGAATCGGAGTTTGTCTATGGTGATTATAAAGTTTATG
ATGTTCGTAAAATGATTGCTAAGTCTGAGCAAGAAATAGGCAAAGCAACCGCAA
AATATTTCTTTTACTCTAATATCATGAACTTCTTCAAAACAGAAATTACACTTGCA
AATGGAGAGATTCGCAAACGCCCTCTAATCGAAACTAATGGGGAAACTGGAGAA
ATTGTCTGGGATAAAGGGCGAGATTTTGCCACAGTGCGCAAAGTATTGTCCATG
CCCCAAGTCAATATTGTCAAGAAAACAGAAGTACAGACAGGCGGATTCTCCAAG
GAGTCAATTTTACCAAAAAGAAATTCGGACAAGCTTATTGCTCGTAAAAAAGACT
GGGATCCAAAAAAATATGGTGGTTTTGATAGTCCAACGGTAGCTTATTCAGTCCT
AGTGGTTGCTAAGGTGGAAAAAGGGAAATCGAAGAAGTTAAAATCCGTTAAAGA
GTTACTAGGGATCACAATTATGGAAAGAAGTTCCTTTGAAAAAAATCCGATTGAC
TTTTTAGAAGCTAAAGGATATAAGGAAGTTAAAAAAGACTTAATCATTAAACTACC
TAAATATAGTCTTTTTGAGTTAGAAAACGGTCGTAAACGGATGCTGGCTAGTGCC
GGAGAATTACAAAAAGGAAATGAGCTGGCTCTGCCAAGCAAATATGTGAATTTTT
TATATTTAGCTAGTCATTATGAAAAGTTGAAGGGTAGTCCAGAAGATAACGAACA
AAAACAATTGTTTGTGGAGCAGCATAAGCATTATTTAGATGAGATTATTGAGCAA
ATCAGTGAATTTTCTAAGCGTGTTATTTTAGCAGATGCCAATTTAGATAAAGTTCT
TAGTGCATATAACAAACATAGAGACAAACCAATACGTGAACAAGCAGAAAATATT
ATTCATTTATTTACGTTGACGAATCTTGGAGCTCCCGCTGCTTTTAAATATTTTGA
TACAACAATTGATCGTAAACGATATACGTCTACAAAAGAAGTTTTAGATGCCACT
CTTATCCATCAATCCATCACTGGTCTTTATGAAACACGCATTGATTTGAGTCAGC
TAGGAGGTGACTGAAGTATATTTTAGATGAAGATTATTTCTTAATAACTAAAAATA
TGGTATAATACTCTTAATAAATGCAGTAATACAGGGGCTTTTCAAGACTGAAGTC
TAGCTGAGACAAATAGTGCGATTACGAAATTTTTTAGACAAAAATAGTCTACGAG
GTTTTAGAGCTATGCTGTTTTGAATGGTCCCAAAACTGAGACCAGTCTCGGAAG
CTCAAAGGTCTCGTTTTAGAGCTATGCTGTTTTGAATGGTCCCAAAACTTCAGCA
CACTGAGACTTGTTGAGTTCCATGTTTTAGAGCTATGCTGTTTTGAATGGACTCC
ATTCAACATTGCCGATGATAACTTGAGAAAGAGGGTTAATACCAGCAGTCGGAT
ACCTTCCTATTCTTTCTGTTAAAGCGTTTTCATGTTATAATAGGCAAAAGAAGAGT
AGTGTGATCGTCCATTCCGACAGCATCGCCAGTCACTATGGCGTGCTGCTAGCG
CTATATGCGTTGATGCAATTTCTATGCGCACCCGTTCTCGGAGCACTGTCCGAC
CGCTTTGGCCGCCGCCCAGTCCTGCTCGCTTCGCTACTTGGAGCCACTATCGA
CTACGCGATCATGGCGACCACACCCGTCCTGTGGATCCTCTACGCCGGACGCA
TCGTGGCCGGCATCACCGGCGCCACAGGTGCGGTTGCTGGCGCCTATATCGCC
GACATCACCGATGGGGAAGATCGGGCTCGCCACTTCGGGCTCATGAGCGCTTG
TTTCGGCGTGGGTATGGTGGCAGGCCCCGTGGCCGGGGGACTGTTGGGCGCC
ATCTCCTTGCATGCACCATTCCTTGCGGCGGCGGTGCTCAACGGCCTCAACCTA
CTACTGGGCTGCTTCCTAATGCAGGAGTCGCATAAGGGAGAGCGTCGACCGAT
GCCCTTGAGAGCCTTCAACCCAGTCAGCTCCTTCCGGTGGGCGCGGGGCATGA
CTATCGTCGCCGCACTTATGACTGTCTTCTTTATCATGCAACTCGTAGGACAGGT
GCCGGCAGCGCTCTGGGTCATTTTCGGCGAGGACCGCTTTCGCTGGAGCGCGA
CGATGATCGGCCTGTCGCTTGCGGTATTCGGAATCTTGCACGCCCTCGCTCAAG
CCTTCGTCACTGGTCCCGCCACCAAACGTTTCGGCGAGAAGCAGGCCATTATC
GCCGGCATGGCGGCCGACGCGCTGGGCTACGTCTTGCTGGCGTTCGCGACGC
GAGGCTGGATGGCCTTCCCCATTATGATTCTTCTCGCTTCCGGCGGCATCGGGA

TGCCCGCGTTGCAGGCCATGCTGTCCAGGCAGGTAGATGACGACCATCAGGGA
CAGCTTCAAGGATCGCTCGCGGCTCTTACCAGCCTAACTTCGATCATTGGACCG
CTGATCGTCACGGCGATTTATGCCGCCTCGGCGAGCACATGGAACGGGTTGGC
ATGGATTGTAGGCGCCGCCCTATACCTTGTCTGCCTCCCCGCGTTGCGTCGCG
GTGCATGGAGCCGGGCCACCTCGACCTGAATGGAAGCCGGCGGCACCTCGCT
AACGGATTCACCACTCCAAGAATTGGAGCCAATCAATTCTTGCGGAGAACTGTG
AATGCGCAAACCAACCCTTGGCAGAACATATCCATCGCGTCCGCCATCTCCAGC
AGCCGCACGCGGCGCATCTCGGGCAGCGTTGGGTCCTGGCCACGGGTGCGCA
TGATCGTGCTCCTGTCGTTGAGGACCCGGCTAGGCTGGCGGGGTTGCCTTACT
GGTTAGCAGAATGAATCACCGATACGCGAGCGAACGTGAAGCGACTGCTGCTG
CAAAACGTCTGCGACCTGAGCAACAACATGAATGGTCTTCGGTTTCCGTGTTTC
GTAAAGTCTGGAAACGCGGAAGTCCCCTACGTGCTGCTGAAGTTGCCCGCAAC
AGAGAGTGGAACCAACCGGTGATACCACGATACTATGACTGAGAGTCAACGCC
ATGAGCGGCCTCATTTCTTATTCTGAGTTACAACAGTCCGCACCGCTGTCCGGT
AGCTCCTTCCGGTGGGCGCGGGGCATGACTATCGTCGCCGCACTTATGACTGT
CTTCTTTATCATGCAACTCGTAGGACAGGTGCCGGCAGCGCCCAACAGTCCCCC
GGCCACGGGGCCTGCCACCATACCCACGCCGAAACAAGCGCCCTGCACCATTA
TGTTCCGGATCTGCATCGCAGGATGCTGCTGGCTACCCTGTGGAACACCTACAT
CTGTATTAACGAAGCGCTAACCGTTTTTATCAGGCTCTGGGAGGCAGAATAAAT
GATCATATCGTCAATTATTACCTCCACGGGGAGAGCCTGAGCAAACTGGCCTCA
GGCATTTGAGAAGCACACGGTCACACTGCTTCCGGTAGTCAATAAACCGGTAAA
CCAGCAATAGACATAAGCGGCTATTTAACGACCCTGCCCTGAACCGACGACCG
GGTCGAATTTGCTTTCGAATTTCTGCCATTCATCCGCTTATTATCACTTATTCAGG
CGTAGCACCAGGCGTTTAAGGGCACCAATAACTGCCTTAAAAAAATTACGCCCC
GCCCTGCCACTCATCGCAGTACTGTTGTAATTCATTAAGCATTCTGCCGACATG
GAAGCCATCACAGACGGCATGATGAACCTGAATCGCCAGCGGCATCAGCACCT
TGTCGCCTTGCGTATAATATTTGCCCATGGTGAAAACGGGGGCGAAGAAGTTGT
CCATATTGGCCACGTTTAAATCAAAACTGGTGAAACTCACCCAGGGATTGGCTG
AGACGAAAAACATATTCTCAATAAACCCTTTAGGGAAATAGGCCAGGTTTTCACC
GTAACACGCCACATCTTGCGAATATATGTGTAGAAACTGCCGGAAATCGTCGTG
GTATTCACTCCAGAGCGATGAAAACGTTTCAGTTTGCTCATGGAAAACGGTGTA
ACAAGGGTGAACACTATCCCATATCACCAGCTCACCGTCTTTCATTGCCATACG

pRC2303

GAATTCCGGATGAGCATTCATCAGGCGGGCAAGAATGTGAATAAAGGCCGGAT
AAAACTTGTGCTTATTTTTCTTTACGGTCTTTAAAAAGGCCGTAATATCCAGCTGA
ACGGTCTGGTTATAGGTACATTGAGCAACTGACTGAAATGCCTCAAAATGTTCTT
TACGATGCCATTGGGATATATCAACGGTGGTATATCCAGTGATTTTTTTCTCCAT
TTTAGCTTCCTTAGCTCCTGAAAATCTCGATAACTCAAAAAATACGCCCGGTAGT
GATCTTATTTCATTATGGTGAAAGTTGGAACCTCTTACGTGCCGATCAACGTCTC
ATTTTCGCCAAAAGTTGGCCCAGGGCTTCCCGGTATCAACAGGGACACCAGGAT
TTATTTATTCTGCGAAGTGATCTTCCGTCACAGGTATTTATTCGGCGCAAAGTGC
GTCGGGTGATGCTGCCAACTTACTGATTTAGTGTATGATGGTGTTTTTGAGGTG

CTCCAGTGGCTTCTGTTTCTATCAGCTGTCCCTCCTGTTCAGCTACTGACGGGG
TGGTGCGTAACGGCAAAAGCACCGCCGGACATCAGCGCTAGCGGAGTGTATAC
TGGCTTACTATGTTGGCACTGATGAGGGTGTCAGTGAAGTGCTTCATGTGGCAG
GAGAAAAAAGGCTGCACCGGTGCGTCAGCAGAATATGTGATACAGGATATATTC
CGCTTCCTCGCTCACTGACTCGCTACGCTCGGTCGTTCGACTGCGGCGAGCGG
AAATGGCTTACGAACGGGGCGGAGATTTCCTGGAAGATGCCAGGAAGATACTTA
ACAGGGAAGTGAGAGGGCCGCGGCAAAGCCGTTTTTCCATAGGCTCCGCCCCC
CTGACAAGCATCACGAAATCTGACGCTCAAATCAGTGGTGGCGAAACCCGACA
GGACTATAAAGATACCAGGCGTTTCCCCCTGGCGGCTCCCTCGTGCGCTCTCCT
GTTCCTGCCTTTCGGTTTACCGGTGTCATTCCGCTGTTATGGCCGCGTTTGTCT
CATTCCACGCCTGACACTCAGTTCCGGGTAGGCAGTTCGCTCCAAGCTGGACT
GTATGCACGAACCCCCCGTTCAGTCCGACCGCTGCGCCTTATCCGGTAACTATC
GTCTTGAGTCCAACCCGGAAAGACATGCAAAAGCACCACTGGCAGCAGCCACT
GGTAATTGATTTAGAGGAGTTAGTCTTGAAGTCATGCGCCGGTTAAGGCTAAAC
TGAAAGGACAAGTTTTGGTGACTGCGCTCCTCCAAGCCAGTTACCTCGGTTCAA
AGAGTTGGTAGCTCAGAGAACCTTCGAAAAACCGCCCTGCAAGGCGGTTTTTTC
GTTTTCAGAGCAAGAGATTACGCGCAGACCAAAACGATCTCAAGAAGATCATCT
TATTAATCAGATAAAATATTTCTAGATTTCAGTGCAATTTATCTCTTCAAATGTAG
CACCTGAAGTCAGCCCCATACGATATAAGTTGTAATTCTCATGTTTGACAGCTTA
TCATCGATAAGCTTTAATGCGGTAGTTTATCACAGTTAAATTGCTAACGCAGTCA
GGCACCGTGTATGAAATCTAACAATGCGCTCATCGTCATCCTCGGCACCGTCAC
CCTGGATGCTGTAGGCATAGGCTTGGTTATGCCGGTACTGCCGGGCCTCTTGC
GGGATTACGAAATCATCCTGTGGAGCTTAGTAGGTTTAGCAAGATGGCAGCGCC
TAAATGTAGAATGATAAAAGGATTAAGAGATTAATTTCCCTAAAAATGATAAAACA
AGCGTTTTGAAAGCGCTTGTTTTTTTGGTTTGCAGTCAGAGTAGAATAGAAGTAT
CAAAAAAAGCACCGACTCGGTGCCACTTTTTCAAGTTGATAACGGACTAGCCTT
ATTTTAACTTGCTATGCTGTTTTGAATGGTTCCAACAAGATTATTTTATAACTTTTA
TAACAAATAATCAAGGAGAAATTCAAAGAAATTTATCAGCCATAAAACAATACTTA
ATACTATAGAATGATAACAAAATAAACTACTTTTTAAAAGAATTTTGTGTTATAATC
TATTTATTATTAAGTATTGGGTAATATTTTTTGAAGAGATATTTTGAAAAAGAAAAA
TTAAAGCATATTAAACTAATTTCGGAGGTCATTAAAACTATTATTGAAATCATCAA
ACTCATTATGGATTTAATTTAAACTTTTTATTTTAGGAGGCAAAAATGGATAAGAA
ATACTCAATAGGCTTAGCTATCGGCACAAATAGCGTCGGATGGGCGGTGATCAC
TGATGAATATAAGGTTCCGTCTAAAAAGTTCAAGGTTCTGGGAAATACAGACCG
CCACAGTATCAAAAAAAATCTTATAGGGGCTCTTTTATTTGACAGTGGAGAGACA
GCGGAAGCGACTCGTCTCAAACGGACAGCTCGTAGAAGGTATACACGTCGGAA
GAATCGTATTTGTTATCTACAGGAGATTTTTTCAAATGAGATGGCGAAAGTAGAT
GATAGTTTCTTTCATCGACTTGAAGAGTCTTTTTTGGTGGAAGAAGACAAGAAGC
ATGAACGTCATCCTATTTTTGGAAATATAGTAGATGAAGTTGCTTATCATGAGAA
ATATCCAACTATCTATCATCTGCGAAAAAAATTGGTAGATTCTACTGATAAAGCG
GATTTGCGCTTAATCTATTTGGCCTTAGCGCATATGATTAAGTTTCGTGGTCATT
TTTTGATTGAGGGAGATTTAAATCCTGATAATAGTGATGTGGACAAACTATTTAT
CCAGTTGGTACAAACCTACAATCAATTATTTGAAGAAAACCCTATTAACGCAAGT
GGAGTAGATGCTAAAGCGATTCTTTCTGCACGATTGAGTAAATCAAGACGATTA

GAAAATCTCATTGCTCAGCTCCCCGGTGAGAAGAAAAATGGCTTATTTGGGAAT
CTCATTGCTTTGTCATTGGGTTTGACCCCTAATTTTAAATCAAATTTTGATTTGGC
AGAAGATGCTAAATTACAGCTTTCAAAAGATACTTACGATGATGATTTAGATAATT
TATTGGCGCAAATTGGAGATCAATATGCTGATTTGTTTTTGGCAGCTAAGAATTT
ATCAGATGCTATTTTACTTTCAGATATCCTAAGAGTAAATACTGAAATAACTAAGG
CTCCCCTATCAGCTTCAATGATTAAACGCTACGATGAACATCATCAAGACTTGAC
TCTTTTAAAAGCTTTAGTTCGACAACAACTTCCAGAAAAGTATAAAGAAATCTTTT
TTGATCAATCAAAAAACGGATATGCAGGTTATATTGATGGGGGAGCTAGCCAAG
AAGAATTTTATAAATTTATCAAACCAATTTTAGAAAAAATGGATGGTACTGAGGAA
TTATTGGTGAAACTAAATCGTGAAGATTTGCTGCGCAAGCAACGGACCTTTGAC
AACGGCTCTATTCCCCATCAAATTCACTTGGGTGAGCTGCATGCTATTTTGAGAA
GACAAGAAGACTTTTATCCATTTTTAAAAGACAATCGTGAGAAGATTGAAAAAAT
CTTGACTTTTCGAATTCCTTATTATGTTGGTCCATTGGCGCGTGGCAATAGTCGT
TTTGCATGGATGACTCGGAAGTCTGAAGAAACAATTACCCCATGGAATTTTGAAG
AAGTTGTCGATAAAGGTGCTTCAGCTCAATCATTTATTGAACGCATGACAAACTT
TGATAAAAATCTTCCAAATGAAAAAGTACTACCAAAACATAGTTTGCTTTATGAGT
ATTTTACGGTTTATAACGAATTGACAAAGGTCAAATATGTTACTGAAGGAATGCG
AAAACCAGCATTTCTTTCAGGTGAACAGAAGAAAGCCATTGTTGATTTACTCTTC
AAAACAAATCGAAAAGTAACCGTTAAGCAATTAAAAGAAGATTATTTCAAAAAAAT
AGAATGTTTTGATAGTGTTGAAATTTCAGGAGTTGAAGATAGATTTAATGCTTCAT
TAGGTACCTACCATGATTTGCTAAAAATTATTAAAGATAAAGATTTTTTGGATAAT
GAAGAAAATGAAGATATCTTAGAGGATATTGTTTTAACATTGACCTTATTTGAAGA
TAGGGAGATGATTGAGGAAAGACTTAAAACATATGCTCACCTCTTTGATGATAAG
GTGATGAAACAGCTTAAACGTCGCCGTTATACTGGTTGGGGACGTTTGTCTCGA
AAATTGATTAATGGTATTAGGGATAAGCAATCTGGCAAAACAATATTAGATTTTTT
GAAATCAGATGGTTTTGCCAATCGCAATTTTATGCAGCTGATCCATGATGATAGT
TTGACATTTAAAGAAGACATTCAAAAAGCACAAGTGTCTGGACAAGGCGATAGTT
TACATGAACATATTGCAAATTTAGCTGGTAGCCCTGCTATTAAAAAAGGTATTTTA
CAGACTGTAAAAGTTGTTGATGAATTGGTCAAAGTAATGGGGCGGCATAAGCCA
GAAAATATCGTTATTGAAATGGCACGTGAAAATCAGACAACTCAAAAGGGCCAG
AAAAATTCGCGAGAGCGTATGAAACGAATCGAAGAAGGTATCAAAGAATTAGGA
AGTCAGATTCTTAAAGAGCATCCTGTTGAAAATACTCAATTGCAAAATGAAAAGC
TCTATCTCTATTATCTCCAAAATGGAAGAGACATGTATGTGGACCAAGAATTAGA
TATTAATCGTTTAAGTGATTATGATGTCGATGCCATTGTTCCACAAAGTTTCCTTA
AAGACGATTCAATAGACAATAAGGTCTTAACGCGTTCTGATAAAAATCGTGGTAA
ATCGGATAACGTTCCAAGTGAAGAAGTAGTCAAAAAGATGAAAAACTATTGGAG
ACAACTTCTAAACGCCAAGTTAATCACTCAACGTAAGTTTGATAATTTAACGAAA
GCTGAACGTGGAGGTTTGAGTGAACTTGATAAAGCTGGTTTTATCAAACGCCAA
TTGGTTGAAACTCGCCAAATCACTAAGCATGTGGCACAAATTTTGGATAGTCGCA
TGAATACTAAATACGATGAAAATGATAAACTTATTCGAGAGGTTAAAGTGATTAC
CTTAAAATCTAAATTAGTTTCTGACTTCCGAAAAGATTTCCAATTCTATAAAGTAC
GTGAGATTAACAATTACCATCATGCCCATGATGCGTATCTAAATGCCGTCGTTGG
AACTGCTTTGATTAAGAAATATCCAAAACTTGAATCGGAGTTTGTCTATGGTGAT
TATAAAGTTTATGATGTTCGTAAAATGATTGCTAAGTCTGAGCAAGAAATAGGCA

AAGCAACCGCAAAATATTTCTTTTACTCTAATATCATGAACTTCTTCAAAACAGAA
ATTACACTTGCAAATGGAGAGATTCGCAAACGCCCTCTAATCGAAACTAATGGG
GAAACTGGAGAAATTGTCTGGGATAAAGGGCGAGATTTTGCCACAGTGCGCAAA
GTATTGTCCATGCCCCAAGTCAATATTGTCAAGAAAACAGAAGTACAGACAGGC
GGATTCTCCAAGGAGTCAATTTTACCAAAAAGAAATTCGGACAAGCTTATTGCTC
GTAAAAAAGACTGGGATCCAAAAAAATATGGTGGTTTTGATAGTCCAACGGTAG
CTTATTCAGTCCTAGTGGTTGCTAAGGTGGAAAAAGGGAAATCGAAGAAGTTAA
AATCCGTTAAAGAGTTACTAGGGATCACAATTATGGAAAGAAGTTCCTTTGAAAA
AAATCCGATTGACTTTTTAGAAGCTAAAGGATATAAGGAAGTTAAAAAAGACTTA
ATCATTAAACTACCTAAATATAGTCTTTTTGAGTTAGAAAACGGTCGTAAACGGA
TGCTGGCTAGTGCCGGAGAATTACAAAAAGGAAATGAGCTGGCTCTGCCAAGC
AAATATGTGAATTTTTTATATTTAGCTAGTCATTATGAAAAGTTGAAGGGTAGTCC
AGAAGATAACGAACAAAAACAATTGTTTGTGGAGCAGCATAAGCATTATTTAGAT
GAGATTATTGAGCAAATCAGTGAATTTTCTAAGCGTGTTATTTTAGCAGATGCCA
ATTTAGATAAAGTTCTTAGTGCATATAACAAACATAGAGACAAACCAATACGTGA
ACAAGCAGAAAATATTATTCATTTATTTACGTTGACGAATCTTGGAGCTCCCGCT
GCTTTTAAATATTTTGATACAACAATTGATCGTAAACGATATACGTCTACAAAAGA
AGTTTTAGATGCCACTCTTATCCATCAATCCATCACTGGTCTTTATGAAACACGC
ATTGATTTGAGTCAGCTAGGAGGTCCTAGGGGAGGTGGCTCAGAAGGTGGCGG
ATCTGAAGGTGGCTCTGGAactagtATGAGCGATAAAATTATTCACCTGACTGACG
ACAGTTTTGACACGGATGTACTCAAAGCGGACGGGGCGATCCTCGTCGATTTCT
GGGCAGAGTGGTGCGGTCCGTGCAAAATGATCGCCCCGATTCTGGATGAAATC
GCTGACGAATATCAGGGCAAACTGACCGTTGCAAAACTGAACATCGATCAAAAC
CCTGGCACTGCGCCGAAATATGGCATCCGTGGTATCCCGACTCTGCTGCTGTTC
AAAAACGGTGAAGTGGCGGCAACCAAAGTGGGTGCACTGTCTAAAGGTCAGTT
GAAAGAGTTCCTCGACGCTAACCTGGCCGGTTCTGGTTCTGGCCATATGCACCA
TCATCATCATCATTCTTCTGGTCTGGTGCCACGCGGTTCTGGTATGAAAGAAAC
CGCTGCTGCTAAATTCGAACGCCAGCACATGGACAGCCCAGATCTGGGTACCG
ACGACGACGACAAGGCCATGGCTGATATCggatccCATATGGAAATGATGCTCGAT
AAGAAACAGATTCGTGCGATCTTTCTCTTTGAGTTTAAAATGGGTCGCAAAGCG
GCGGAGACGACGCGTAATATTAACAACGCGTTCGGTCCTGGCACCGCGAACGA
GCGTACCGTGCAATGGTGGTTCAAAAAGTTTCGCAAAGGCGACGAATCTCTGGA
GGACGAAGAGCGTTCTGGCCGCCCGTCCGAGGTTGACAACGACCAGCTGCGTG
CAATCATCGAAGCTGATCCGCTGACTACCACCCGCGAAGTTGCTGAAGAACTGA
ATGTGGATCACTCTACTGTGGTTCGCCACCTGAAACAGATCGGTAAAGTAAAAA
AACTGGACAAATGGGTTCCTCATGAACTGTCTGAAAACCAGAAAAACCGTCGTT
TCGAAGTTAGCTCCTCTCTGATTCTGCGTAACAACAACGAACCGTTCCTGGATC
GTATCGTAACCTGTGATGAGAAATGGATTCTGTATGATAACCGTCGCCGCTCTG
CTCAGTGGCTGGATCGCGAAGAAGCTCCAAAACACTTCCCGAAACCGAATCTGC
ACCAGAAGAAAGTCATGGTAACCGTATGGTGGTCTGCCGCAGGTGTTATCCACT
ATTCCTTCCTGAACCCGGGCGAAACTATCACCAGCGAAAAATACTGCCAGCAGA
TTGACGAAATGCACCGTAAACTGCAGCGTCTGCAGCCAGCACTGGTGAATCGTA
AAGGTCCGATCCTGCTGCATGATAACGCCCGTCCGCACGTTGCCCAACCGACC
CTGCAGAAACTGAACGAACTGGGCTATGAAGTTCTGCCACACCCGCCGTACTCC

CCGGATCTGTCCCCGACTGACTACCATTTCTTCAAGCATCTGGACAACTTCCTG
CAGGGTAAACGTTTTCACAACCAACAGGACGCAGAAAACGCTTTCCAGGAGTTC
GTCGAAAGCCGTTCCACTGACTTCTACGCGACCGGTATCAACAAGCTGATCAGC
CGTTGGCAGAAATGCGTGGACTGTAACGGCAGCTACTTCGATTAAGACGTCTTA
ATAACTAAAAATATGGTATAATACTCTTAATAAATGCAGTAATACAGGGGCTTTTC
AAGACTGAAGTCTAGCTGAGACAAATAGTGCGATTACGAAATTTTTTAGACAAAA
ATAGTCTACGAGGTTTTAGAGCTATGCTGTTTTGAATGGTCCCAAAACTGAGACC
AGTCTCGGAAGCTCAAAGGTCTCGTTTTAGAGCTATGCTGTTTTGAATGGTCCC
AAAACTTCAGCACACTGAGACTTGTTGAGTTCCATGTTTTAGAGCTATGCTGTTT
TGAATGGACTCCATTCAACATTGCCGATGATAACTTGAGAAAGAGGGTTAATACC
AGCAGTCGGATACCTTCCTATTCTTTCTGTTAAAGCGTTTTCATGTTATAATAGG
CAAAAGAAGAGTAGTGTGATCGTCCATTCCGACAGCATCGCCAGTCACTATGGC
GTGCTGCTAGCGCTATATGCGTTGATGCAATTTCTATGCGCACCCGTTCTCGGA
GCACTGTCCGACCGCTTTGGCCGCCGCCCAGTCCTGCTCGCTTCGCTACTTGG
AGCCACTATCGACTACGCGATCATGGCGACCACACCCGTCCTGTGGATCCTCTA
CGCCGGACGCATCGTGGCCGGCATCACCGGCGCCACAGGTGCGGTTGCTGGC
GCCTATATCGCCGACATCACCGATGGGGAAGATCGGGCTCGCCACTTCGGGCT
CATGAGCGCTTGTTTCGGCGTGGGTATGGTGGCAGGCCCCGTGGCCGGGGGA
CTGTTGGGCGCCATCTCCTTGCATGCACCATTCCTTGCGGCGGCGGTGCTCAA
CGGCCTCAACCTACTACTGGGCTGCTTCCTAATGCAGGAGTCGCATAAGGGAG
AGCGTCGACCGATGCCCTTGAGAGCCTTCAACCCAGTCAGCTCCTTCCGGTGG
GCGCGGGGCATGACTATCGTCGCCGCACTTATGACTGTCTTCTTTATCATGCAA
CTCGTAGGACAGGTGCCGGCAGCGCTCTGGGTCATTTTCGGCGAGGACCGCTT
TCGCTGGAGCGCGACGATGATCGGCCTGTCGCTTGCGGTATTCGGAATCTTGC
ACGCCCTCGCTCAAGCCTTCGTCACTGGTCCCGCCACCAAACGTTTCGGCGAG
AAGCAGGCCATTATCGCCGGCATGGCGGCCGACGCGCTGGGCTACGTCTTGCT
GGCGTTCGCGACGCGAGGCTGGATGGCCTTCCCCATTATGATTCTTCTCGCTTC
CGGCGGCATCGGGATGCCCGCGTTGCAGGCCATGCTGTCCAGGCAGGTAGAT
GACGACCATCAGGGACAGCTTCAAGGATCGCTCGCGGCTCTTACCAGCCTAAC
TTCGATCATTGGACCGCTGATCGTCACGGCGATTTATGCCGCCTCGGCGAGCA
CATGGAACGGGTTGGCATGGATTGTAGGCGCCGCCCTATACCTTGTCTGCCTC
CCCGCGTTGCGTCGCGGTGCATGGAGCCGGGCCACCTCGACCTGAATGGAAG
CCGGCGGCACCTCGCTAACGGATTCACCACTCCAAGAATTGGAGCCAATCAATT
CTTGCGGAGAACTGTGAATGCGCAAACCAACCCTTGGCAGAACATATCCATCGC
GTCCGCCATCTCCAGCAGCCGCACGCGGCGCATCTCGGGCAGCGTTGGGTCCT
GGCCACGGGTGCGCATGATCGTGCTCCTGTCGTTGAGGACCCGGCTAGGCTGG
CGGGGTTGCCTTACTGGTTAGCAGAATGAATCACCGATACGCGAGCGAACGTG
AAGCGACTGCTGCTGCAAAACGTCTGCGACCTGAGCAACAACATGAATGGTCTT
CGGTTTCCGTGTTTCGTAAAGTCTGGAAACGCGGAAGTCCCCTACGTGCTGCTG
AAGTTGCCCGCAACAGAGAGTGGAACCAACCGGTGATACCACGATACTATGACT
GAGAGTCAACGCCATGAGCGGCCTCATTTCTTATTCTGAGTTACAACAGTCCGC
ACCGCTGTCCGGTAGCTCCTTCCGGTGGGCGCGGGGCATGACTATCGTCGCCG
CACTTATGACTGTCTTCTTTATCATGCAACTCGTAGGACAGGTGCCGGCAGCGC
CCAACAGTCCCCCGGCCACGGGGCCTGCCACCATACCCACGCCGAAACAAGC

GCCCTGCACCATTATGTTCCGGATCTGCATCGCAGGATGCTGCTGGCTACCCTG
TGGAACACCTACATCTGTATTAACGAAGCGCTAACCGTTTTTATCAGGCTCTGG
GAGGCAGAATAAATGATCATATCGTCAATTATTACCTCCACGGGGAGAGCCTGA
GCAAACTGGCCTCAGGCATTTGAGAAGCACACGGTCACACTGCTTCCGGTAGTC
AATAAACCGGTAAACCAGCAATAGACATAAGCGGCTATTTAACGACCCTGCCCT
GAACCGACGACCGGGTCGAATTTGCTTTCGAATTTCTGCCATTCATCCGCTTATT
ATCACTTATTCAGGCGTAGCACCAGGCGTTTAAGGGCACCAATAACTGCCTTAA
AAAAATTACGCCCCGCCCTGCCACTCATCGCAGTACTGTTGTAATTCATTAAGCA
TTCTGCCGACATGGAAGCCATCACAGACGGCATGATGAACCTGAATCGCCAGC
GGCATCAGCACCTTGTCGCCTTGCGTATAATATTTGCCCATGGTGAAAACGGGG
GCGAAGAAGTTGTCCATATTGGCCACGTTTAAATCAAAACTGGTGAAACTCACC
CAGGGATTGGCTGAGACGAAAAACATATTCTCAATAAACCCTTTAGGGAAATAG
GCCAGGTTTTCACCGTAACACGCCACATCTTGCGAATATATGTGTAGAAACTGC
CGGAAATCGTCGTGGTATTCACTCCAGAGCGATGAAAACGTTTCAGTTTGCTCA
TGGAAAACGGTGTAACAAGGGTGAACACTATCCCATATCACCAGCTCACCGTCT
TTCATTGCCATACG

pRC2304

GAATTCCGGATGAGCATTCATCAGGCGGGCAAGAATGTGAATAAAGGCCGGAT
AAAACTTGTGCTTATTTTTCTTTACGGTCTTTAAAAAGGCCGTAATATCCAGCTGA
ACGGTCTGGTTATAGGTACATTGAGCAACTGACTGAAATGCCTCAAAATGTTCTT
TACGATGCCATTGGGATATATCAACGGTGGTATATCCAGTGATTTTTTTCTCCAT
TTTAGCTTCCTTAGCTCCTGAAAATCTCGATAACTCAAAAAATACGCCCGGTAGT
GATCTTATTTCATTATGGTGAAAGTTGGAACCTCTTACGTGCCGATCAACGTCTC
ATTTTCGCCAAAAGTTGGCCCAGGGCTTCCCGGTATCAACAGGGACACCAGGAT
TTATTTATTCTGCGAAGTGATCTTCCGTCACAGGTATTTATTCGGCGCAAAGTGC
GTCGGGTGATGCTGCCAACTTACTGATTTAGTGTATGATGGTGTTTTTGAGGTG
CTCCAGTGGCTTCTGTTTCTATCAGCTGTCCCTCCTGTTCAGCTACTGACGGGG
TGGTGCGTAACGGCAAAAGCACCGCCGGACATCAGCGCTAGCGGAGTGTATAC
TGGCTTACTATGTTGGCACTGATGAGGGTGTCAGTGAAGTGCTTCATGTGGCAG
GAGAAAAAAGGCTGCACCGGTGCGTCAGCAGAATATGTGATACAGGATATATTC
CGCTTCCTCGCTCACTGACTCGCTACGCTCGGTCGTTCGACTGCGGCGAGCGG
AAATGGCTTACGAACGGGGCGGAGATTTCCTGGAAGATGCCAGGAAGATACTTA
ACAGGGAAGTGAGAGGGCCGCGGCAAAGCCGTTTTTCCATAGGCTCCGCCCCC
CTGACAAGCATCACGAAATCTGACGCTCAAATCAGTGGTGGCGAAACCCGACA
GGACTATAAAGATACCAGGCGTTTCCCCCTGGCGGCTCCCTCGTGCGCTCTCCT
GTTCCTGCCTTTCGGTTTACCGGTGTCATTCCGCTGTTATGGCCGCGTTTGTCT
CATTCCACGCCTGACACTCAGTTCCGGGTAGGCAGTTCGCTCCAAGCTGGACT
GTATGCACGAACCCCCCGTTCAGTCCGACCGCTGCGCCTTATCCGGTAACTATC
GTCTTGAGTCCAACCCGGAAAGACATGCAAAAGCACCACTGGCAGCAGCCACT
GGTAATTGATTTAGAGGAGTTAGTCTTGAAGTCATGCGCCGGTTAAGGCTAAAC
TGAAAGGACAAGTTTTGGTGACTGCGCTCCTCCAAGCCAGTTACCTCGGTTCAA
AGAGTTGGTAGCTCAGAGAACCTTCGAAAAACCGCCCTGCAAGGCGGTTTTTTC

GTTTTCAGAGCAAGAGATTACGCGCAGACCAAAACGATCTCAAGAAGATCATCT
TATTAATCAGATAAAATATTTCTAGATTTCAGTGCAATTTATCTCTTCAAATGTAG
CACCTGAAGTCAGCCCCATACGATATAAGTTGTAATTCTCATGTTTGACAGCTTA
TCATCGATAAGCTTTAATGCGGTAGTTTATCACAGTTAAATTGCTAACGCAGTCA
GGCACCGTGTATGAAATCTAACAATGCGCTCATCGTCATCCTCGGCACCGTCAC
CCTGGATGCTGTAGGCATAGGCTTGGTTATGCCGGTACTGCCGGGCCTCTTGC
GGGATTACGAAATCATCCTGTGGAGCTTAGTAGGTTTAGCAAGATGGCAGCGCC
TAAATGTAGAATGATAAAAGGATTAAGAGATTAATTTCCCTAAAAATGATAAAACA
AGCGTTTTGAAAGCGCTTGTTTTTTTGGTTTGCAGTCAGAGTAGAATAGAAGTAT
CAAAAAAAGCACCGACTCGGTGCCACTTTTTCAAGTTGATAACGGACTAGCCTT
ATTTTAACTTGCTATGCTGTTTTGAATGGTTCCAACAAGATTATTTTATAACTTTTA
TAACAAATAATCAAGGAGAAATTCAAAGAAATTTATCAGCCATAAAACAATACTTA
ATACTATAGAATGATAACAAAATAAACTACTTTTTAAAAGAATTTTGTGTTATAATC
TATTTATTATTAAGTATTGGGTAATATTTTTTGAAGAGATATTTTGAAAAAGAAAAA
TTAAAGCATATTAAACTAATTTCGGAGGTCATTAAAACTATTATTGAAATCATCAA
ACTCATGCATCATATGGAAATGATGCTCGATAAGAAACAGATTCGTGCGATCTTT
CTCTTTGAGTTTAAAATGGGTCGCAAAGCGGCGGAGACGACGCGTAATATTAAC
AACGCGTTCGGTCCTGGCACCGCGAACGAGCGTACCGTGCAATGGTGGTTCAA
AAAGTTTCGCAAAGGCGACGAATCTCTGGAGGACGAAGAGCGTTCTGGCCGCC
CGTCCGAGGTTGACAACGACCAGCTGCGTGCAATCATCGAAGCTGATCCGCTG
ACTACCACCCGCGAAGTTGCTGAAGAACTGAATGTGGATCACTCTACTGTGGTT
CGCCACCTGAAACAGATCGGTAAAGTAAAAAAACTGGACAAATGGGTTCCTCAT
GAACTGTCTGAAAACCAGAAAAACCGTCGTTTCGAAGTTAGCTCCTCTCTGATTC
TGCGTAACAACAACGAACCGTTCCTGGATCGTATCGTAACCTGTGATGAGAAAT
GGATTCTGTATGATAACCGTCGCCGCTCTGCTCAGTGGCTGGATCGCGAAGAA
GCTCCAAAACACTTCCCGAAACCGAATCTGCACCAGAAGAAAGTCATGGTAACC
GTATGGTGGTCTGCCGCAGGTGTTATCCACTATTCCTTCCTGAACCCGGGCGAA
ACTATCACCAGCGAAAAATACTGCCAGCAGATTGACGAAATGCACCGTAAACTG
CAGCGTCTGCAGCCAGCACTGGTGAATCGTAAAGGTCCGATCCTGCTGCATGAT
AACGCCCGTCCGCACGTTGCCCAACCGACCCTGCAGAAACTGAACGAACTGGG
CTATGAAGTTCTGCCACACCCGCCGTACTCCCCGGATCTGTCCCCGACTGACTA
CCATTTCTTCAAGCATCTGGACAACTTCCTGCAGGGTAAACGTTTTCACAACCAA
CAGGACGCAGAAAACGCTTTCCAGGAGTTCGTCGAAAGCCGTTCCACTGACTTC
TACGCGACCGGTATCAACAAGCTGATCAGCCGTTGGCAGAAATGCGTGGACTG
TAACGGCAGCTACTTCGATtctagaGGAGGTGGCTCAGAAGGTGGCGGATCTGAA
GGTGGCTCTGGAactagtATGAGCGATAAAATTATTCACCTGACTGACGACAGTTT
TGACACGGATGTACTCAAAGCGGACGGGGCGATCCTCGTCGATTTCTGGGCAG
AGTGGTGCGGTCCGTGCAAAATGATCGCCCCGATTCTGGATGAAATCGCTGAC
GAATATCAGGGCAAACTGACCGTTGCAAAACTGAACATCGATCAAAACCCTGGC
ACTGCGCCGAAATATGGCATCCGTGGTATCCCGACTCTGCTGCTGTTCAAAAAC
GGTGAAGTGGCGGCAACCAAAGTGGGTGCACTGTCTAAAGGTCAGTTGAAAGA
GTTCCTCGACGCTAACCTGGCCGGTTCTGGTTCTGGCCATATGCACCATCATCA
TCATCATTCTTCTGGTCTGGTGCCACGCGGTTCTGGTATGAAAGAAACCGCTGC
TGCTAAATTCGAACGCCAGCACATGGACAGCCCAGATCTGGGTACCGACGACG

ACGACAAGGCCATGGCTGATATCCTCGAGATGGATAAGAAATACTCAATAGGCT
TAGCTATCGGCACAAATAGCGTCGGATGGGCGGTGATCACTGATGAATATAAGG
TTCCGTCTAAAAAGTTCAAGGTTCTGGGAAATACAGACCGCCACAGTATCAAAA
AAAATCTTATAGGGGCTCTTTTATTTGACAGTGGAGAGACAGCGGAAGCGACTC
GTCTCAAACGGACAGCTCGTAGAAGGTATACACGTCGGAAGAATCGTATTTGTT
ATCTACAGGAGATTTTTTCAAATGAGATGGCGAAAGTAGATGATAGTTTCTTTCA
TCGACTTGAAGAGTCTTTTTTGGTGGAAGAAGACAAGAAGCATGAACGTCATCC
TATTTTTGGAAATATAGTAGATGAAGTTGCTTATCATGAGAAATATCCAACTATCT
ATCATCTGCGAAAAAAATTGGTAGATTCTACTGATAAAGCGGATTTGCGCTTAAT
CTATTTGGCCTTAGCGCATATGATTAAGTTTCGTGGTCATTTTTTGATTGAGGGA
GATTTAAATCCTGATAATAGTGATGTGGACAAACTATTTATCCAGTTGGTACAAA
CCTACAATCAATTATTTGAAGAAAACCCTATTAACGCAAGTGGAGTAGATGCTAA
AGCGATTCTTTCTGCACGATTGAGTAAATCAAGACGATTAGAAAATCTCATTGCT
CAGCTCCCCGGTGAGAAGAAAAATGGCTTATTTGGGAATCTCATTGCTTTGTCAT
TGGGTTTGACCCCTAATTTTAAATCAAATTTTGATTTGGCAGAAGATGCTAAATTA
CAGCTTTCAAAAGATACTTACGATGATGATTTAGATAATTTATTGGCGCAAATTG
GAGATCAATATGCTGATTTGTTTTTGGCAGCTAAGAATTTATCAGATGCTATTTTA
CTTTCAGATATCCTAAGAGTAAATACTGAAATAACTAAGGCTCCCCTATCAGCTT
CAATGATTAAACGCTACGATGAACATCATCAAGACTTGACTCTTTTAAAAGCTTT
AGTTCGACAACAACTTCCAGAAAAGTATAAAGAAATCTTTTTTGATCAATCAAAAA
ACGGATATGCAGGTTATATTGATGGGGGAGCTAGCCAAGAAGAATTTTATAAATT
TATCAAACCAATTTTAGAAAAAATGGATGGTACTGAGGAATTATTGGTGAAACTA
AATCGTGAAGATTTGCTGCGCAAGCAACGGACCTTTGACAACGGCTCTATTCCC
CATCAAATTCACTTGGGTGAGCTGCATGCTATTTTGAGAAGACAAGAAGACTTTT
ATCCATTTTTAAAAGACAATCGTGAGAAGATTGAAAAAATCTTGACTTTTCGAATT
CCTTATTATGTTGGTCCATTGGCGCGTGGCAATAGTCGTTTTGCATGGATGACT
CGGAAGTCTGAAGAAACAATTACCCCATGGAATTTTGAAGAAGTTGTCGATAAA
GGTGCTTCAGCTCAATCATTTATTGAACGCATGACAAACTTTGATAAAAATCTTC
CAAATGAAAAAGTACTACCAAAACATAGTTTGCTTTATGAGTATTTTACGGTTTAT
AACGAATTGACAAAGGTCAAATATGTTACTGAAGGAATGCGAAAACCAGCATTTC
TTTCAGGTGAACAGAAGAAAGCCATTGTTGATTTACTCTTCAAAACAAATCGAAA
AGTAACCGTTAAGCAATTAAAAGAAGATTATTTCAAAAAAATAGAATGTTTTGATA
GTGTTGAAATTTCAGGAGTTGAAGATAGATTTAATGCTTCATTAGGTACCTACCA
TGATTTGCTAAAAATTATTAAAGATAAAGATTTTTTGGATAATGAAGAAAATGAAG
ATATCTTAGAGGATATTGTTTTAACATTGACCTTATTTGAAGATAGGGAGATGATT
GAGGAAAGACTTAAAACATATGCTCACCTCTTTGATGATAAGGTGATGAAACAG
CTTAAACGTCGCCGTTATACTGGTTGGGGACGTTTGTCTCGAAAATTGATTAATG
GTATTAGGGATAAGCAATCTGGCAAAACAATATTAGATTTTTTGAAATCAGATGG
TTTTGCCAATCGCAATTTTATGCAGCTGATCCATGATGATAGTTTGACATTTAAA
GAAGACATTCAAAAAGCACAAGTGTCTGGACAAGGCGATAGTTTACATGAACAT
ATTGCAAATTTAGCTGGTAGCCCTGCTATTAAAAAAGGTATTTTACAGACTGTAA
AAGTTGTTGATGAATTGGTCAAAGTAATGGGGCGGCATAAGCCAGAAAATATCG
TTATTGAAATGGCACGTGAAAATCAGACAACTCAAAAGGGCCAGAAAAATTCGC
GAGAGCGTATGAAACGAATCGAAGAAGGTATCAAAGAATTAGGAAGTCAGATTC

TTAAAGAGCATCCTGTTGAAAATACTCAATTGCAAAATGAAAAGCTCTATCTCTAT
TATCTCCAAAATGGAAGAGACATGTATGTGGACCAAGAATTAGATATTAATCGTT
TAAGTGATTATGATGTCGATGCCATTGTTCCACAAAGTTTCCTTAAAGACGATTC
AATAGACAATAAGGTCTTAACGCGTTCTGATAAAAATCGTGGTAAATCGGATAAC
GTTCCAAGTGAAGAAGTAGTCAAAAAGATGAAAAACTATTGGAGACAACTTCTAA
ACGCCAAGTTAATCACTCAACGTAAGTTTGATAATTTAACGAAAGCTGAACGTGG
AGGTTTGAGTGAACTTGATAAAGCTGGTTTTATCAAACGCCAATTGGTTGAAACT
CGCCAAATCACTAAGCATGTGGCACAAATTTTGGATAGTCGCATGAATACTAAAT
ACGATGAAAATGATAAACTTATTCGAGAGGTTAAAGTGATTACCTTAAAATCTAA
ATTAGTTTCTGACTTCCGAAAAGATTTCCAATTCTATAAAGTACGTGAGATTAACA
ATTACCATCATGCCCATGATGCGTATCTAAATGCCGTCGTTGGAACTGCTTTGAT
TAAGAAATATCCAAAACTTGAATCGGAGTTTGTCTATGGTGATTATAAAGTTTATG
ATGTTCGTAAAATGATTGCTAAGTCTGAGCAAGAAATAGGCAAAGCAACCGCAA
AATATTTCTTTTACTCTAATATCATGAACTTCTTCAAAACAGAAATTACACTTGCA
AATGGAGAGATTCGCAAACGCCCTCTAATCGAAACTAATGGGGAAACTGGAGAA
ATTGTCTGGGATAAAGGGCGAGATTTTGCCACAGTGCGCAAAGTATTGTCCATG
CCCCAAGTCAATATTGTCAAGAAAACAGAAGTACAGACAGGCGGATTCTCCAAG
GAGTCAATTTTACCAAAAAGAAATTCGGACAAGCTTATTGCTCGTAAAAAAGACT
GGGATCCAAAAAAATATGGTGGTTTTGATAGTCCAACGGTAGCTTATTCAGTCCT
AGTGGTTGCTAAGGTGGAAAAAGGGAAATCGAAGAAGTTAAAATCCGTTAAAGA
GTTACTAGGGATCACAATTATGGAAAGAAGTTCCTTTGAAAAAAATCCGATTGAC
TTTTTAGAAGCTAAAGGATATAAGGAAGTTAAAAAAGACTTAATCATTAAACTACC
TAAATATAGTCTTTTTGAGTTAGAAAACGGTCGTAAACGGATGCTGGCTAGTGCC
GGAGAATTACAAAAAGGAAATGAGCTGGCTCTGCCAAGCAAATATGTGAATTTTT
TATATTTAGCTAGTCATTATGAAAAGTTGAAGGGTAGTCCAGAAGATAACGAACA
AAAACAATTGTTTGTGGAGCAGCATAAGCATTATTTAGATGAGATTATTGAGCAA
ATCAGTGAATTTTCTAAGCGTGTTATTTTAGCAGATGCCAATTTAGATAAAGTTCT
TAGTGCATATAACAAACATAGAGACAAACCAATACGTGAACAAGCAGAAAATATT
ATTCATTTATTTACGTTGACGAATCTTGGAGCTCCCGCTGCTTTTAAATATTTTGA
TACAACAATTGATCGTAAACGATATACGTCTACAAAAGAAGTTTTAGATGCCACT
CTTATCCATCAATCCATCACTGGTCTTTATGAAACACGCATTGATTTGAGTCAGC
TAGGAGGTGACTGAAGTATATTTTAGATGAAGATTATTTCTTAATAACTAAAAATA
TGGTATAATACTCTTAATAAATGCAGTAATACAGGGGCTTTTCAAGACTGAAGTC
TAGCTGAGACAAATAGTGCGATTACGAAATTTTTTAGACAAAAATAGTCTACGAG
GTTTTAGAGCTATGCTGTTTTGAATGGTCCCAAAACGGTTTTCCCAGTCACGACG
TTGTAAAACGAGTTTTAGAGCTATGCTGTTTTGAATGGTCCCAAAACTTCAGCAC
ACTGAGACTTGTTGAGTTCCATGTTTTAGAGCTATGCTGTTTTGAATGGACTCCA
TTCAACATTGCCGATGATAACTTGAGAAAGAGGGTTAATACCAGCAGTCGGATA
CCTTCCTATTCTTTCTGTTAAAGCGTTTTCATGTTATAATAGGCAAAAGAAGAGTA
GTGTGATCGTCCATTCCGACAGCATCGCCAGTCACTATGGCGTGCTGCTAGCG
CTATATGCGTTGATGCAATTTCTATGCGCACCCGTTCTCGGAGCACTGTCCGAC
CGCTTTGGCCGCCGCCCAGTCCTGCTCGCTTCGCTACTTGGAGCCACTATCGA
CTACGCGATCATGGCGACCACACCCGTCCTGTGGATCCTCTACGCCGGACGCA
TCGTGGCCGGCATCACCGGCGCCACAGGTGCGGTTGCTGGCGCCTATATCGCC

GACATCACCGATGGGGAAGATCGGGCTCGCCACTTCGGGCTCATGAGCGCTTG
TTTCGGCGTGGGTATGGTGGCAGGCCCCGTGGCCGGGGGACTGTTGGGCGCC
ATCTCCTTGCATGCACCATTCCTTGCGGCGGCGGTGCTCAACGGCCTCAACCTA
CTACTGGGCTGCTTCCTAATGCAGGAGTCGCATAAGGGAGAGCGTCGACCGAT
GCCCTTGAGAGCCTTCAACCCAGTCAGCTCCTTCCGGTGGGCGCGGGGCATGA
CTATCGTCGCCGCACTTATGACTGTCTTCTTTATCATGCAACTCGTAGGACAGGT
GCCGGCAGCGCTCTGGGTCATTTTCGGCGAGGACCGCTTTCGCTGGAGCGCGA
CGATGATCGGCCTGTCGCTTGCGGTATTCGGAATCTTGCACGCCCTCGCTCAAG
CCTTCGTCACTGGTCCCGCCACCAAACGTTTCGGCGAGAAGCAGGCCATTATC
GCCGGCATGGCGGCCGACGCGCTGGGCTACGTCTTGCTGGCGTTCGCGACGC
GAGGCTGGATGGCCTTCCCCATTATGATTCTTCTCGCTTCCGGCGGCATCGGGA
TGCCCGCGTTGCAGGCCATGCTGTCCAGGCAGGTAGATGACGACCATCAGGGA
CAGCTTCAAGGATCGCTCGCGGCTCTTACCAGCCTAACTTCGATCATTGGACCG
CTGATCGTCACGGCGATTTATGCCGCCTCGGCGAGCACATGGAACGGGTTGGC
ATGGATTGTAGGCGCCGCCCTATACCTTGTCTGCCTCCCCGCGTTGCGTCGCG
GTGCATGGAGCCGGGCCACCTCGACCTGAATGGAAGCCGGCGGCACCTCGCT
AACGGATTCACCACTCCAAGAATTGGAGCCAATCAATTCTTGCGGAGAACTGTG
AATGCGCAAACCAACCCTTGGCAGAACATATCCATCGCGTCCGCCATCTCCAGC
AGCCGCACGCGGCGCATCTCGGGCAGCGTTGGGTCCTGGCCACGGGTGCGCA
TGATCGTGCTCCTGTCGTTGAGGACCCGGCTAGGCTGGCGGGGTTGCCTTACT
GGTTAGCAGAATGAATCACCGATACGCGAGCGAACGTGAAGCGACTGCTGCTG
CAAAACGTCTGCGACCTGAGCAACAACATGAATGGTCTTCGGTTTCCGTGTTTC
GTAAAGTCTGGAAACGCGGAAGTCCCCTACGTGCTGCTGAAGTTGCCCGCAAC
AGAGAGTGGAACCAACCGGTGATACCACGATACTATGACTGAGAGTCAACGCC
ATGAGCGGCCTCATTTCTTATTCTGAGTTACAACAGTCCGCACCGCTGTCCGGT
AGCTCCTTCCGGTGGGCGCGGGGCATGACTATCGTCGCCGCACTTATGACTGT
CTTCTTTATCATGCAACTCGTAGGACAGGTGCCGGCAGCGCCCAACAGTCCCCC
GGCCACGGGGCCTGCCACCATACCCACGCCGAAACAAGCGCCCTGCACCATTA
TGTTCCGGATCTGCATCGCAGGATGCTGCTGGCTACCCTGTGGAACACCTACAT
CTGTATTAACGAAGCGCTAACCGTTTTTATCAGGCTCTGGGAGGCAGAATAAAT
GATCATATCGTCAATTATTACCTCCACGGGGAGAGCCTGAGCAAACTGGCCTCA
GGCATTTGAGAAGCACACGGTCACACTGCTTCCGGTAGTCAATAAACCGGTAAA
CCAGCAATAGACATAAGCGGCTATTTAACGACCCTGCCCTGAACCGACGACCG
GGTCGAATTTGCTTTCGAATTTCTGCCATTCATCCGCTTATTATCACTTATTCAGG
CGTAGCACCAGGCGTTTAAGGGCACCAATAACTGCCTTAAAAAAATTACGCCCC
GCCCTGCCACTCATCGCAGTACTGTTGTAATTCATTAAGCATTCTGCCGACATG
GAAGCCATCACAGACGGCATGATGAACCTGAATCGCCAGCGGCATCAGCACCT
TGTCGCCTTGCGTATAATATTTGCCCATGGTGAAAACGGGGGCGAAGAAGTTGT
CCATATTGGCCACGTTTAAATCAAAACTGGTGAAACTCACCCAGGGATTGGCTG
AGACGAAAAACATATTCTCAATAAACCCTTTAGGGAAATAGGCCAGGTTTTCACC
GTAACACGCCACATCTTGCGAATATATGTGTAGAAACTGCCGGAAATCGTCGTG
GTATTCACTCCAGAGCGATGAAAACGTTTCAGTTTGCTCATGGAAAACGGTGTA
ACAAGGGTGAACACTATCCCATATCACCAGCTCACCGTCTTTCATTGCCATACG

pRC2305

GAATTCCGGATGAGCATTCATCAGGCGGGCAAGAATGTGAATAAAGGCCGGAT
AAAACTTGTGCTTATTTTTCTTTACGGTCTTTAAAAAGGCCGTAATATCCAGCTGA
ACGGTCTGGTTATAGGTACATTGAGCAACTGACTGAAATGCCTCAAAATGTTCTT
TACGATGCCATTGGGATATATCAACGGTGGTATATCCAGTGATTTTTTTCTCCAT
TTTAGCTTCCTTAGCTCCTGAAAATCTCGATAACTCAAAAAATACGCCCGGTAGT
GATCTTATTTCATTATGGTGAAAGTTGGAACCTCTTACGTGCCGATCAACGTCTC
ATTTTCGCCAAAAGTTGGCCCAGGGCTTCCCGGTATCAACAGGGACACCAGGAT
TTATTTATTCTGCGAAGTGATCTTCCGTCACAGGTATTTATTCGGCGCAAAGTGC
GTCGGGTGATGCTGCCAACTTACTGATTTAGTGTATGATGGTGTTTTTGAGGTG
CTCCAGTGGCTTCTGTTTCTATCAGCTGTCCCTCCTGTTCAGCTACTGACGGGG
TGGTGCGTAACGGCAAAAGCACCGCCGGACATCAGCGCTAGCGGAGTGTATAC
TGGCTTACTATGTTGGCACTGATGAGGGTGTCAGTGAAGTGCTTCATGTGGCAG
GAGAAAAAAGGCTGCACCGGTGCGTCAGCAGAATATGTGATACAGGATATATTC
CGCTTCCTCGCTCACTGACTCGCTACGCTCGGTCGTTCGACTGCGGCGAGCGG
AAATGGCTTACGAACGGGGCGGAGATTTCCTGGAAGATGCCAGGAAGATACTTA
ACAGGGAAGTGAGAGGGCCGCGGCAAAGCCGTTTTTCCATAGGCTCCGCCCCC
CTGACAAGCATCACGAAATCTGACGCTCAAATCAGTGGTGGCGAAACCCGACA
GGACTATAAAGATACCAGGCGTTTCCCCCTGGCGGCTCCCTCGTGCGCTCTCCT
GTTCCTGCCTTTCGGTTTACCGGTGTCATTCCGCTGTTATGGCCGCGTTTGTCT
CATTCCACGCCTGACACTCAGTTCCGGGTAGGCAGTTCGCTCCAAGCTGGACT
GTATGCACGAACCCCCCGTTCAGTCCGACCGCTGCGCCTTATCCGGTAACTATC
GTCTTGAGTCCAACCCGGAAAGACATGCAAAAGCACCACTGGCAGCAGCCACT
GGTAATTGATTTAGAGGAGTTAGTCTTGAAGTCATGCGCCGGTTAAGGCTAAAC
TGAAAGGACAAGTTTTGGTGACTGCGCTCCTCCAAGCCAGTTACCTCGGTTCAA
AGAGTTGGTAGCTCAGAGAACCTTCGAAAAACCGCCCTGCAAGGCGGTTTTTTC
GTTTTCAGAGCAAGAGATTACGCGCAGACCAAAACGATCTCAAGAAGATCATCT
TATTAATCAGATAAAATATTTCTAGATTTCAGTGCAATTTATCTCTTCAAATGTAG
CACCTGAAGTCAGCCCCATACGATATAAGTTGTAATTCTCATGTTTGACAGCTTA
TCATCGATAAGCTTTAATGCGGTAGTTTATCACAGTTAAATTGCTAACGCAGTCA
GGCACCGTGTATGAAATCTAACAATGCGCTCATCGTCATCCTCGGCACCGTCAC
CCTGGATGCTGTAGGCATAGGCTTGGTTATGCCGGTACTGCCGGGCCTCTTGC
GGGATTACGAAATCATCCTGTGGAGCTTAGTAGGTTTAGCAAGATGGCAGCGCC
TAAATGTAGAATGATAAAAGGATTAAGAGATTAATTTCCCTAAAAATGATAAAACA
AGCGTTTTGAAAGCGCTTGTTTTTTTGGTTTGCAGTCAGAGTAGAATAGAAGTAT
CAAAAAAAGCACCGACTCGGTGCCACTTTTTCAAGTTGATAACGGACTAGCCTT
ATTTTAACTTGCTATGCTGTTTTGAATGGTTCCAACAAGATTATTTTATAACTTTTA
TAACAAATAATCAAGGAGAAATTCAAAGAAATTTATCAGCCATAAAACAATACTTA
ATACTATAGAATGATAACAAAATAAACTACTTTTTAAAAGAATTTTGTGTTATAATC
TATTTATTATTAAGTATTGGGTAATATTTTTTGAAGAGATATTTTGAAAAAGAAAAA
TTAAAGCATATTAAACTAATTTCGGAGGTCATTAAAACTATTATTGAAATCATCAA
ACTCATTATGGATTTAATTTAAACTTTTTATTTTAGGAGGCAAAAATGGATAAGAA
ATACTCAATAGGCTTAGCTATCGGCACAAATAGCGTCGGATGGGCGGTGATCAC
TGATGAATATAAGGTTCCGTCTAAAAAGTTCAAGGTTCTGGGAAATACAGACCG

CCACAGTATCAAAAAAAATCTTATAGGGGCTCTTTTATTTGACAGTGGAGAGACA
GCGGAAGCGACTCGTCTCAAACGGACAGCTCGTAGAAGGTATACACGTCGGAA
GAATCGTATTTGTTATCTACAGGAGATTTTTTCAAATGAGATGGCGAAAGTAGAT
GATAGTTTCTTTCATCGACTTGAAGAGTCTTTTTTGGTGGAAGAAGACAAGAAGC
ATGAACGTCATCCTATTTTTGGAAATATAGTAGATGAAGTTGCTTATCATGAGAA
ATATCCAACTATCTATCATCTGCGAAAAAAATTGGTAGATTCTACTGATAAAGCG
GATTTGCGCTTAATCTATTTGGCCTTAGCGCATATGATTAAGTTTCGTGGTCATT
TTTTGATTGAGGGAGATTTAAATCCTGATAATAGTGATGTGGACAAACTATTTAT
CCAGTTGGTACAAACCTACAATCAATTATTTGAAGAAAACCCTATTAACGCAAGT
GGAGTAGATGCTAAAGCGATTCTTTCTGCACGATTGAGTAAATCAAGACGATTA
GAAAATCTCATTGCTCAGCTCCCCGGTGAGAAGAAAAATGGCTTATTTGGGAAT
CTCATTGCTTTGTCATTGGGTTTGACCCCTAATTTTAAATCAAATTTTGATTTGGC
AGAAGATGCTAAATTACAGCTTTCAAAAGATACTTACGATGATGATTTAGATAATT
TATTGGCGCAAATTGGAGATCAATATGCTGATTTGTTTTTGGCAGCTAAGAATTT
ATCAGATGCTATTTTACTTTCAGATATCCTAAGAGTAAATACTGAAATAACTAAGG
CTCCCCTATCAGCTTCAATGATTAAACGCTACGATGAACATCATCAAGACTTGAC
TCTTTTAAAAGCTTTAGTTCGACAACAACTTCCAGAAAAGTATAAAGAAATCTTTT
TTGATCAATCAAAAAACGGATATGCAGGTTATATTGATGGGGGAGCTAGCCAAG
AAGAATTTTATAAATTTATCAAACCAATTTTAGAAAAAATGGATGGTACTGAGGAA
TTATTGGTGAAACTAAATCGTGAAGATTTGCTGCGCAAGCAACGGACCTTTGAC
AACGGCTCTATTCCCCATCAAATTCACTTGGGTGAGCTGCATGCTATTTTGAGAA
GACAAGAAGACTTTTATCCATTTTTAAAAGACAATCGTGAGAAGATTGAAAAAAT
CTTGACTTTTCGAATTCCTTATTATGTTGGTCCATTGGCGCGTGGCAATAGTCGT
TTTGCATGGATGACTCGGAAGTCTGAAGAAACAATTACCCCATGGAATTTTGAAG
AAGTTGTCGATAAAGGTGCTTCAGCTCAATCATTTATTGAACGCATGACAAACTT
TGATAAAAATCTTCCAAATGAAAAAGTACTACCAAAACATAGTTTGCTTTATGAGT
ATTTTACGGTTTATAACGAATTGACAAAGGTCAAATATGTTACTGAAGGAATGCG
AAAACCAGCATTTCTTTCAGGTGAACAGAAGAAAGCCATTGTTGATTTACTCTTC
AAAACAAATCGAAAAGTAACCGTTAAGCAATTAAAAGAAGATTATTTCAAAAAAAT
AGAATGTTTTGATAGTGTTGAAATTTCAGGAGTTGAAGATAGATTTAATGCTTCAT
TAGGTACCTACCATGATTTGCTAAAAATTATTAAAGATAAAGATTTTTTGGATAAT
GAAGAAAATGAAGATATCTTAGAGGATATTGTTTTAACATTGACCTTATTTGAAGA
TAGGGAGATGATTGAGGAAAGACTTAAAACATATGCTCACCTCTTTGATGATAAG
GTGATGAAACAGCTTAAACGTCGCCGTTATACTGGTTGGGGACGTTTGTCTCGA
AAATTGATTAATGGTATTAGGGATAAGCAATCTGGCAAAACAATATTAGATTTTTT
GAAATCAGATGGTTTTGCCAATCGCAATTTTATGCAGCTGATCCATGATGATAGT
TTGACATTTAAAGAAGACATTCAAAAAGCACAAGTGTCTGGACAAGGCGATAGTT
TACATGAACATATTGCAAATTTAGCTGGTAGCCCTGCTATTAAAAAAGGTATTTTA
CAGACTGTAAAAGTTGTTGATGAATTGGTCAAAGTAATGGGGCGGCATAAGCCA
GAAAATATCGTTATTGAAATGGCACGTGAAAATCAGACAACTCAAAAGGGCCAG
AAAAATTCGCGAGAGCGTATGAAACGAATCGAAGAAGGTATCAAAGAATTAGGA
AGTCAGATTCTTAAAGAGCATCCTGTTGAAAATACTCAATTGCAAAATGAAAAGC
TCTATCTCTATTATCTCCAAAATGGAAGAGACATGTATGTGGACCAAGAATTAGA
TATTAATCGTTTAAGTGATTATGATGTCGATGCCATTGTTCCACAAAGTTTCCTTA

AAGACGATTCAATAGACAATAAGGTCTTAACGCGTTCTGATAAAAATCGTGGTAA
ATCGGATAACGTTCCAAGTGAAGAAGTAGTCAAAAAGATGAAAAACTATTGGAG
ACAACTTCTAAACGCCAAGTTAATCACTCAACGTAAGTTTGATAATTTAACGAAA
GCTGAACGTGGAGGTTTGAGTGAACTTGATAAAGCTGGTTTTATCAAACGCCAA
TTGGTTGAAACTCGCCAAATCACTAAGCATGTGGCACAAATTTTGGATAGTCGCA
TGAATACTAAATACGATGAAAATGATAAACTTATTCGAGAGGTTAAAGTGATTAC
CTTAAAATCTAAATTAGTTTCTGACTTCCGAAAAGATTTCCAATTCTATAAAGTAC
GTGAGATTAACAATTACCATCATGCCCATGATGCGTATCTAAATGCCGTCGTTGG
AACTGCTTTGATTAAGAAATATCCAAAACTTGAATCGGAGTTTGTCTATGGTGAT
TATAAAGTTTATGATGTTCGTAAAATGATTGCTAAGTCTGAGCAAGAAATAGGCA
AAGCAACCGCAAAATATTTCTTTTACTCTAATATCATGAACTTCTTCAAAACAGAA
ATTACACTTGCAAATGGAGAGATTCGCAAACGCCCTCTAATCGAAACTAATGGG
GAAACTGGAGAAATTGTCTGGGATAAAGGGCGAGATTTTGCCACAGTGCGCAAA
GTATTGTCCATGCCCCAAGTCAATATTGTCAAGAAAACAGAAGTACAGACAGGC
GGATTCTCCAAGGAGTCAATTTTACCAAAAAGAAATTCGGACAAGCTTATTGCTC
GTAAAAAAGACTGGGATCCAAAAAAATATGGTGGTTTTGATAGTCCAACGGTAG
CTTATTCAGTCCTAGTGGTTGCTAAGGTGGAAAAAGGGAAATCGAAGAAGTTAA
AATCCGTTAAAGAGTTACTAGGGATCACAATTATGGAAAGAAGTTCCTTTGAAAA
AAATCCGATTGACTTTTTAGAAGCTAAAGGATATAAGGAAGTTAAAAAAGACTTA
ATCATTAAACTACCTAAATATAGTCTTTTTGAGTTAGAAAACGGTCGTAAACGGA
TGCTGGCTAGTGCCGGAGAATTACAAAAAGGAAATGAGCTGGCTCTGCCAAGC
AAATATGTGAATTTTTTATATTTAGCTAGTCATTATGAAAAGTTGAAGGGTAGTCC
AGAAGATAACGAACAAAAACAATTGTTTGTGGAGCAGCATAAGCATTATTTAGAT
GAGATTATTGAGCAAATCAGTGAATTTTCTAAGCGTGTTATTTTAGCAGATGCCA
ATTTAGATAAAGTTCTTAGTGCATATAACAAACATAGAGACAAACCAATACGTGA
ACAAGCAGAAAATATTATTCATTTATTTACGTTGACGAATCTTGGAGCTCCCGCT
GCTTTTAAATATTTTGATACAACAATTGATCGTAAACGATATACGTCTACAAAAGA
AGTTTTAGATGCCACTCTTATCCATCAATCCATCACTGGTCTTTATGAAACACGC
ATTGATTTGAGTCAGCTAGGAGGTCCTAGGGGAGGTGGCTCAGAAGGTGGCGG
ATCTGAAGGTGGCTCTGGAactagtATGAGCGATAAAATTATTCACCTGACTGACG
ACAGTTTTGACACGGATGTACTCAAAGCGGACGGGGCGATCCTCGTCGATTTCT
GGGCAGAGTGGTGCGGTCCGTGCAAAATGATCGCCCCGATTCTGGATGAAATC
GCTGACGAATATCAGGGCAAACTGACCGTTGCAAAACTGAACATCGATCAAAAC
CCTGGCACTGCGCCGAAATATGGCATCCGTGGTATCCCGACTCTGCTGCTGTTC
AAAAACGGTGAAGTGGCGGCAACCAAAGTGGGTGCACTGTCTAAAGGTCAGTT
GAAAGAGTTCCTCGACGCTAACCTGGCCGGTTCTGGTTCTGGCCATATGCACCA
TCATCATCATCATTCTTCTGGTCTGGTGCCACGCGGTTCTGGTATGAAAGAAAC
CGCTGCTGCTAAATTCGAACGCCAGCACATGGACAGCCCAGATCTGGGTACCG
ACGACGACGACAAGGCCATGGCTGATATCggatccCATATGGAAATGATGCTCGAT
AAGAAACAGATTCGTGCGATCTTTCTCTTTGAGTTTAAAATGGGTCGCAAAGCG
GCGGAGACGACGCGTAATATTAACAACGCGTTCGGTCCTGGCACCGCGAACGA
GCGTACCGTGCAATGGTGGTTCAAAAAGTTTCGCAAAGGCGACGAATCTCTGGA
GGACGAAGAGCGTTCTGGCCGCCCGTCCGAGGTTGACAACGACCAGCTGCGTG
CAATCATCGAAGCTGATCCGCTGACTACCACCCGCGAAGTTGCTGAAGAACTGA

ATGTGGATCACTCTACTGTGGTTCGCCACCTGAAACAGATCGGTAAAGTAAAAA
AACTGGACAAATGGGTTCCTCATGAACTGTCTGAAAACCAGAAAAACCGTCGTT
TCGAAGTTAGCTCCTCTCTGATTCTGCGTAACAACAACGAACCGTTCCTGGATC
GTATCGTAACCTGTGATGAGAAATGGATTCTGTATGATAACCGTCGCCGCTCTG
CTCAGTGGCTGGATCGCGAAGAAGCTCCAAAACACTTCCCGAAACCGAATCTGC
ACCAGAAGAAAGTCATGGTAACCGTATGGTGGTCTGCCGCAGGTGTTATCCACT
ATTCCTTCCTGAACCCGGGCGAAACTATCACCAGCGAAAAATACTGCCAGCAGA
TTGACGAAATGCACCGTAAACTGCAGCGTCTGCAGCCAGCACTGGTGAATCGTA
AAGGTCCGATCCTGCTGCATGATAACGCCCGTCCGCACGTTGCCCAACCGACC
CTGCAGAAACTGAACGAACTGGGCTATGAAGTTCTGCCACACCCGCCGTACTCC
CCGGATCTGTCCCCGACTGACTACCATTTCTTCAAGCATCTGGACAACTTCCTG
CAGGGTAAACGTTTTCACAACCAACAGGACGCAGAAAACGCTTTCCAGGAGTTC
GTCGAAAGCCGTTCCACTGACTTCTACGCGACCGGTATCAACAAGCTGATCAGC
CGTTGGCAGAAATGCGTGGACTGTAACGGCAGCTACTTCGATTAAGACGTCTTA
ATAACTAAAAATATGGTATAATACTCTTAATAAATGCAGTAATACAGGGGCTTTTC
AAGACTGAAGTCTAGCTGAGACAAATAGTGCGATTACGAAATTTTTTAGACAAAA
ATAGTCTACGAGGTTTTAGAGCTATGCTGTTTTGAATGGTCCCAAAACGGTTTTC
CCAGTCACGACGTTGTAAAACGAGTTTTAGAGCTATGCTGTTTTGAATGGTCCCA
AAACTTCAGCACACTGAGACTTGTTGAGTTCCATGTTTTAGAGCTATGCTGTTTT
GAATGGACTCCATTCAACATTGCCGATGATAACTTGAGAAAGAGGGTTAATACC
AGCAGTCGGATACCTTCCTATTCTTTCTGTTAAAGCGTTTTCATGTTATAATAGG
CAAAAGAAGAGTAGTGTGATCGTCCATTCCGACAGCATCGCCAGTCACTATGGC
GTGCTGCTAGCGCTATATGCGTTGATGCAATTTCTATGCGCACCCGTTCTCGGA
GCACTGTCCGACCGCTTTGGCCGCCGCCCAGTCCTGCTCGCTTCGCTACTTGG
AGCCACTATCGACTACGCGATCATGGCGACCACACCCGTCCTGTGGATCCTCTA
CGCCGGACGCATCGTGGCCGGCATCACCGGCGCCACAGGTGCGGTTGCTGGC
GCCTATATCGCCGACATCACCGATGGGGAAGATCGGGCTCGCCACTTCGGGCT
CATGAGCGCTTGTTTCGGCGTGGGTATGGTGGCAGGCCCCGTGGCCGGGGGA
CTGTTGGGCGCCATCTCCTTGCATGCACCATTCCTTGCGGCGGCGGTGCTCAA
CGGCCTCAACCTACTACTGGGCTGCTTCCTAATGCAGGAGTCGCATAAGGGAG
AGCGTCGACCGATGCCCTTGAGAGCCTTCAACCCAGTCAGCTCCTTCCGGTGG
GCGCGGGGCATGACTATCGTCGCCGCACTTATGACTGTCTTCTTTATCATGCAA
CTCGTAGGACAGGTGCCGGCAGCGCTCTGGGTCATTTTCGGCGAGGACCGCTT
TCGCTGGAGCGCGACGATGATCGGCCTGTCGCTTGCGGTATTCGGAATCTTGC
ACGCCCTCGCTCAAGCCTTCGTCACTGGTCCCGCCACCAAACGTTTCGGCGAG
AAGCAGGCCATTATCGCCGGCATGGCGGCCGACGCGCTGGGCTACGTCTTGCT
GGCGTTCGCGACGCGAGGCTGGATGGCCTTCCCCATTATGATTCTTCTCGCTTC
CGGCGGCATCGGGATGCCCGCGTTGCAGGCCATGCTGTCCAGGCAGGTAGAT
GACGACCATCAGGGACAGCTTCAAGGATCGCTCGCGGCTCTTACCAGCCTAAC
TTCGATCATTGGACCGCTGATCGTCACGGCGATTTATGCCGCCTCGGCGAGCA
CATGGAACGGGTTGGCATGGATTGTAGGCGCCGCCCTATACCTTGTCTGCCTC
CCCGCGTTGCGTCGCGGTGCATGGAGCCGGGCCACCTCGACCTGAATGGAAG
CCGGCGGCACCTCGCTAACGGATTCACCACTCCAAGAATTGGAGCCAATCAATT
CTTGCGGAGAACTGTGAATGCGCAAACCAACCCTTGGCAGAACATATCCATCGC

GTCCGCCATCTCCAGCAGCCGCACGCGGCGCATCTCGGGCAGCGTTGGGTCCT
GGCCACGGGTGCGCATGATCGTGCTCCTGTCGTTGAGGACCCGGCTAGGCTGG
CGGGGTTGCCTTACTGGTTAGCAGAATGAATCACCGATACGCGAGCGAACGTG
AAGCGACTGCTGCTGCAAAACGTCTGCGACCTGAGCAACAACATGAATGGTCTT
CGGTTTCCGTGTTTCGTAAAGTCTGGAAACGCGGAAGTCCCCTACGTGCTGCTG
AAGTTGCCCGCAACAGAGAGTGGAACCAACCGGTGATACCACGATACTATGACT
GAGAGTCAACGCCATGAGCGGCCTCATTTCTTATTCTGAGTTACAACAGTCCGC
ACCGCTGTCCGGTAGCTCCTTCCGGTGGGCGCGGGGCATGACTATCGTCGCCG
CACTTATGACTGTCTTCTTTATCATGCAACTCGTAGGACAGGTGCCGGCAGCGC
CCAACAGTCCCCCGGCCACGGGGCCTGCCACCATACCCACGCCGAAACAAGC
GCCCTGCACCATTATGTTCCGGATCTGCATCGCAGGATGCTGCTGGCTACCCTG
TGGAACACCTACATCTGTATTAACGAAGCGCTAACCGTTTTTATCAGGCTCTGG
GAGGCAGAATAAATGATCATATCGTCAATTATTACCTCCACGGGGAGAGCCTGA
GCAAACTGGCCTCAGGCATTTGAGAAGCACACGGTCACACTGCTTCCGGTAGTC
AATAAACCGGTAAACCAGCAATAGACATAAGCGGCTATTTAACGACCCTGCCCT
GAACCGACGACCGGGTCGAATTTGCTTTCGAATTTCTGCCATTCATCCGCTTATT
ATCACTTATTCAGGCGTAGCACCAGGCGTTTAAGGGCACCAATAACTGCCTTAA
AAAAATTACGCCCCGCCCTGCCACTCATCGCAGTACTGTTGTAATTCATTAAGCA
TTCTGCCGACATGGAAGCCATCACAGACGGCATGATGAACCTGAATCGCCAGC
GGCATCAGCACCTTGTCGCCTTGCGTATAATATTTGCCCATGGTGAAAACGGGG
GCGAAGAAGTTGTCCATATTGGCCACGTTTAAATCAAAACTGGTGAAACTCACC
CAGGGATTGGCTGAGACGAAAAACATATTCTCAATAAACCCTTTAGGGAAATAG
GCCAGGTTTTCACCGTAACACGCCACATCTTGCGAATATATGTGTAGAAACTGC
CGGAAATCGTCGTGGTATTCACTCCAGAGCGATGAAAACGTTTCAGTTTGCTCA
TGGAAAACGGTGTAACAAGGGTGAACACTATCCCATATCACCAGCTCACCGTCT
TTCATTGCCATACG

pRC2306

GAATTCCGGATGAGCATTCATCAGGCGGGCAAGAATGTGAATAAAGGCCGGAT
AAAACTTGTGCTTATTTTTCTTTACGGTCTTTAAAAAGGCCGTAATATCCAGCTGA
ACGGTCTGGTTATAGGTACATTGAGCAACTGACTGAAATGCCTCAAAATGTTCTT
TACGATGCCATTGGGATATATCAACGGTGGTATATCCAGTGATTTTTTTCTCCAT
TTTAGCTTCCTTAGCTCCTGAAAATCTCGATAACTCAAAAAATACGCCCGGTAGT
GATCTTATTTCATTATGGTGAAAGTTGGAACCTCTTACGTGCCGATCAACGTCTC
ATTTTCGCCAAAAGTTGGCCCAGGGCTTCCCGGTATCAACAGGGACACCAGGAT
TTATTTATTCTGCGAAGTGATCTTCCGTCACAGGTATTTATTCGGCGCAAAGTGC
GTCGGGTGATGCTGCCAACTTACTGATTTAGTGTATGATGGTGTTTTTGAGGTG
CTCCAGTGGCTTCTGTTTCTATCAGCTGTCCCTCCTGTTCAGCTACTGACGGGG
TGGTGCGTAACGGCAAAAGCACCGCCGGACATCAGCGCTAGCGGAGTGTATAC
TGGCTTACTATGTTGGCACTGATGAGGGTGTCAGTGAAGTGCTTCATGTGGCAG
GAGAAAAAAGGCTGCACCGGTGCGTCAGCAGAATATGTGATACAGGATATATTC
CGCTTCCTCGCTCACTGACTCGCTACGCTCGGTCGTTCGACTGCGGCGAGCGG
AAATGGCTTACGAACGGGGCGGAGATTTCCTGGAAGATGCCAGGAAGATACTTA

ACAGGGAAGTGAGAGGGCCGCGGCAAAGCCGTTTTTCCATAGGCTCCGCCCCC
CTGACAAGCATCACGAAATCTGACGCTCAAATCAGTGGTGGCGAAACCCGACA
GGACTATAAAGATACCAGGCGTTTCCCCCTGGCGGCTCCCTCGTGCGCTCTCCT
GTTCCTGCCTTTCGGTTTACCGGTGTCATTCCGCTGTTATGGCCGCGTTTGTCT
CATTCCACGCCTGACACTCAGTTCCGGGTAGGCAGTTCGCTCCAAGCTGGACT
GTATGCACGAACCCCCCGTTCAGTCCGACCGCTGCGCCTTATCCGGTAACTATC
GTCTTGAGTCCAACCCGGAAAGACATGCAAAAGCACCACTGGCAGCAGCCACT
GGTAATTGATTTAGAGGAGTTAGTCTTGAAGTCATGCGCCGGTTAAGGCTAAAC
TGAAAGGACAAGTTTTGGTGACTGCGCTCCTCCAAGCCAGTTACCTCGGTTCAA
AGAGTTGGTAGCTCAGAGAACCTTCGAAAAACCGCCCTGCAAGGCGGTTTTTTC
GTTTTCAGAGCAAGAGATTACGCGCAGACCAAAACGATCTCAAGAAGATCATCT
TATTAATCAGATAAAATATTTCTAGATTTCAGTGCAATTTATCTCTTCAAATGTAG
CACCTGAAGTCAGCCCCATACGATATAAGTTGTAATTCTCATGTTTGACAGCTTA
TCATCGATAAGCTTTAATGCGGTAGTTTATCACAGTTAAATTGCTAACGCAGTCA
GGCACCGTGTATGAAATCTAACAATGCGCTCATCGTCATCCTCGGCACCGTCAC
CCTGGATGCTGTAGGCATAGGCTTGGTTATGCCGGTACTGCCGGGCCTCTTGC
GGGATTACGAAATCATCCTGTGGAGCTTAGTAGGTTTAGCAAGATGGCAGCGCC
TAAATGTAGAATGATAAAAGGATTAAGAGATTAATTTCCCTAAAAATGATAAAACA
AGCGTTTTGAAAGCGCTTGTTTTTTTGGTTTGCAGTCAGAGTAGAATAGAAGTAT
CAAAAAAAGCACCGACTCGGTGCCACTTTTTCAAGTTGATAACGGACTAGCCTT
ATTTTAACTTGCTATGCTGTTTTGAATGGTTCCAACAAGATTATTTTATAACTTTTA
TAACAAATAATCAAGGAGAAATTCAAAGAAATTTATCAGCCATAAAACAATACTTA
ATACTATAGAATGATAACAAAATAAACTACTTTTTAAAAGAATTTTGTGTTATAATC
TATTTATTATTAAGTATTGGGTAATATTTTTTGAAGAGATATTTTGAAAAAGAAAAA
TTAAAGCATATTAAACTAATTTCGGAGGTCATTAAAACTATTATTGAAATCATCAA
ACTCATTATGGATTTAATTTAAACTTTTTATTTTAGGAGGCAACATATGGAAATGA
TGCTCGATAAGAAACAGATTCGTGCGATCTTTCTCTTTGAGTTTAAAATGGGTCG
CAAAGCGGCGGAGACGACGCGTAATATTAACAACGCGTTCGGTCCTGGCACCG
CGAACGAGCGTACCGTGCAATGGTGGTTCAAAAAGTTTCGCAAAGGCGACGAA
TCTCTGGAGGACGAAGAGCGTTCTGGCCGCCCGTCCGAGGTTGACAACGACCA
GCTGCGTGCAATCATCGAAGCTGATCCGCTGACTACCACCCGCGAAGTTGCTG
AAGAACTGAATGTGGATCACTCTACTGTGGTTCGCCACCTGAAACAGATCGGTA
AAGTAAAAAAACTGGACAAATGGGTTCCTCATGAACTGTCTGAAAACCAGAAAAA
CCGTCGTTTCGAAGTTAGCTCCTCTCTGATTCTGCGTAACAACAACGAACCGTT
CCTGGATCGTATCGTAACCTGTGATGAGAAATGGATTCTGTATGATAACCGTCG
CCGCTCTGCTCAGTGGCTGGATCGCGAAGAAGCTCCAAAACACTTCCCGAAAC
CGAATCTGCACCAGAAGAAAGTCATGGTAACCGTATGGTGGTCTGCCGCAGGT
GTTATCCACTATTCCTTCCTGAACCCGGGCGAAACTATCACCAGCGAAAAATACT
GCCAGCAGATTGACGAAATGCACCGTAAACTGCAGCGTCTGCAGCCAGCACTG
GTGAATCGTAAAGGTCCGATCCTGCTGCATGATAACGCCCGTCCGCACGTTGCC
CAACCGACCCTGCAGAAACTGAACGAACTGGGCTATGAAGTTCTGCCACACCC
GCCGTACTCCCCGGATCTGTCCCCGACTGACTACCATTTCTTCAAGCATCTGGA
CAACTTCCTGCAGGGTAAACGTTTTCACAACCAACAGGACGCAGAAAACGCTTT
CCAGGAGTTCGTCGAAAGCCGTTCCACTGACTTCTACGCGACCGGTATCAACAA

GCTGATCAGCCGTTGGCAGAAATGCGTGGACTGTAACGGCAGCTACTTCGATTA
AGGATCCTCTAGAGTCGACCGATGCCCTTGAGAGCCTTCAACCCAGTCAGCTCC
TTCCGGTGGGCGCGGGGCATGACTATCGTCGCCGCACTTATGACTGTCTTCTTT
ATCATGCAACTCGTAGGACAGGTGCCGGCAGCGCTCTGGGTCATTTTCGGCGA
GGACCGCTTTCGCTGGAGCGCGACGATGATCGGCCTGTCGCTTGCGGTATTCG
GAATCTTGCACGCCCTCGCTCAAGCCTTCGTCACTGGTCCCGCCACCAAACGTT
TCGGCGAGAAGCAGGCCATTATCGCCGGCATGGCGGCCGACGCGCTGGGCTA
CGTCTTGCTGGCGTTCGCGACGCGAGGCTGGATGGCCTTCCCCATTATGATTCT
TCTCGCTTCCGGCGGCATCGGGATGCCCGCGTTGCAGGCCATGCTGTCCAGGC
AGGTAGATGACGACCATCAGGGACAGCTTCAAGGATCGCTCGCGGCTCTTACC
AGCCTAACTTCGATCATTGGACCGCTGATCGTCACGGCGATTTATGCCGCCTCG
GCGAGCACATGGAACGGGTTGGCATGGATTGTAGGCGCCGCCCTATACCTTGT
CTGCCTCCCCGCGTTGCGTCGCGGTGCATGGAGCCGGGCCACCTCGACCTGAA
TGGAAGCCGGCGGCACCTCGCTAACGGATTCACCACTCCAAGAATTGGAGCCA
ATCAATTCTTGCGGAGAACTGTGAATGCGCAAACCAACCCTTGGCAGAACATAT
CCATCGCGTCCGCCATCTCCAGCAGCCGCACGCGGCGCATCTCGGGCAGCGTT
GGGTCCTGGCCACGGGTGCGCATGATCGTGCTCCTGTCGTTGAGGACCCGGCT
AGGCTGGCGGGGTTGCCTTACTGGTTAGCAGAATGAATCACCGATACGCGAGC
GAACGTGAAGCGACTGCTGCTGCAAAACGTCTGCGACCTGAGCAACAACATGA
ATGGTCTTCGGTTTCCGTGTTTCGTAAAGTCTGGAAACGCGGAAGTCCCCTACG
TGCTGCTGAAGTTGCCCGCAACAGAGAGTGGAACCAACCGGTGATACCACGAT
ACTATGACTGAGAGTCAACGCCATGAGCGGCCTCATTTCTTATTCTGAGTTACAA
CAGTCCGCACCGCTGTCCGGTAGCTCCTTCCGGTGGGCGCGGGGCATGACTAT
CGTCGCCGCACTTATGACTGTCTTCTTTATCATGCAACTCGTAGGACAGGTGCC
GGCAGCGCCCAACAGTCCCCCGGCCACGGGGCCTGCCACCATACCCACGCCG
AAACAAGCGCCCTGCACCATTATGTTCCGGATCTGCATCGCAGGATGCTGCTGG
CTACCCTGTGGAACACCTACATCTGTATTAACGAAGCGCTAACCGTTTTTATCAG
GCTCTGGGAGGCAGAATAAATGATCATATCGTCAATTATTACCTCCACGGGGAG
AGCCTGAGCAAACTGGCCTCAGGCATTTGAGAAGCACACGGTCACACTGCTTCC
GGTAGTCAATAAACCGGTAAACCAGCAATAGACATAAGCGGCTATTTAACGACC
CTGCCCTGAACCGACGACCGGGTCGAATTTGCTTTCGAATTTCTGCCATTCATC
CGCTTATTATCACTTATTCAGGCGTAGCACCAGGCGTTTAAGGGCACCAATAAC
TGCCTTAAAAAAATTACGCCCCGCCCTGCCACTCATCGCAGTACTGTTGTAATTC
ATTAAGCATTCTGCCGACATGGAAGCCATCACAGACGGCATGATGAACCTGAAT
CGCCAGCGGCATCAGCACCTTGTCGCCTTGCGTATAATATTTGCCCATGGTGAA
AACGGGGGCGAAGAAGTTGTCCATATTGGCCACGTTTAAATCAAAACTGGTGAA
ACTCACCCAGGGATTGGCTGAGACGAAAAACATATTCTCAATAAACCCTTTAGG
GAAATAGGCCAGGTTTTCACCGTAACACGCCACATCTTGCGAATATATGTGTAG
AAACTGCCGGAAATCGTCGTGGTATTCACTCCAGAGCGATGAAAACGTTTCAGT
TTGCTCATGGAAAACGGTGTAACAAGGGTGAACACTATCCCATATCACCAGCTC
ACCGTCTTTCATTGCCATACG

pRC2307

GAATTCCGGATGAGCATTCATCAGGCGGGCAAGAATGTGAATAAAGGCCGGAT
AAAACTTGTGCTTATTTTTCTTTACGGTCTTTAAAAAGGCCGTAATATCCAGCTGA
ACGGTCTGGTTATAGGTACATTGAGCAACTGACTGAAATGCCTCAAAATGTTCTT
TACGATGCCATTGGGATATATCAACGGTGGTATATCCAGTGATTTTTTTCTCCAT
TTTAGCTTCCTTAGCTCCTGAAAATCTCGATAACTCAAAAAATACGCCCGGTAGT
GATCTTATTTCATTATGGTGAAAGTTGGAACCTCTTACGTGCCGATCAACGTCTC
ATTTTCGCCAAAAGTTGGCCCAGGGCTTCCCGGTATCAACAGGGACACCAGGAT
TTATTTATTCTGCGAAGTGATCTTCCGTCACAGGTATTTATTCGGCGCAAAGTGC
GTCGGGTGATGCTGCCAACTTACTGATTTAGTGTATGATGGTGTTTTTGAGGTG
CTCCAGTGGCTTCTGTTTCTATCAGCTGTCCCTCCTGTTCAGCTACTGACGGGG
TGGTGCGTAACGGCAAAAGCACCGCCGGACATCAGCGCTAGCGGAGTGTATAC
TGGCTTACTATGTTGGCACTGATGAGGGTGTCAGTGAAGTGCTTCATGTGGCAG
GAGAAAAAAGGCTGCACCGGTGCGTCAGCAGAATATGTGATACAGGATATATTC
CGCTTCCTCGCTCACTGACTCGCTACGCTCGGTCGTTCGACTGCGGCGAGCGG
AAATGGCTTACGAACGGGGCGGAGATTTCCTGGAAGATGCCAGGAAGATACTTA
ACAGGGAAGTGAGAGGGCCGCGGCAAAGCCGTTTTTCCATAGGCTCCGCCCCC
CTGACAAGCATCACGAAATCTGACGCTCAAATCAGTGGTGGCGAAACCCGACA
GGACTATAAAGATACCAGGCGTTTCCCCCTGGCGGCTCCCTCGTGCGCTCTCCT
GTTCCTGCCTTTCGGTTTACCGGTGTCATTCCGCTGTTATGGCCGCGTTTGTCT
CATTCCACGCCTGACACTCAGTTCCGGGTAGGCAGTTCGCTCCAAGCTGGACT
GTATGCACGAACCCCCCGTTCAGTCCGACCGCTGCGCCTTATCCGGTAACTATC
GTCTTGAGTCCAACCCGGAAAGACATGCAAAAGCACCACTGGCAGCAGCCACT
GGTAATTGATTTAGAGGAGTTAGTCTTGAAGTCATGCGCCGGTTAAGGCTAAAC
TGAAAGGACAAGTTTTGGTGACTGCGCTCCTCCAAGCCAGTTACCTCGGTTCAA
AGAGTTGGTAGCTCAGAGAACCTTCGAAAAACCGCCCTGCAAGGCGGTTTTTTC
GTTTTCAGAGCAAGAGATTACGCGCAGACCAAAACGATCTCAAGAAGATCATCT
TATTAATCAGATAAAATATTTCTAGATTTCAGTGCAATTTATCTCTTCAAATGTAG
CACCTGAAGTCAGCCCCATACGATATAAGTTGTAATTCTCATGTTTGACAGCTTA
TCATCGATAAGCTTTAATGCGGTAGTTTATCACAGTTAAATTGCTAACGCAGTCA
GGCACCGTGTATGAAATCTAACAATGCGCTCATCGTCATCCTCGGCACCGTCAC
CCTGGATGCTGTAGGCATAGGCTTGGTTATGCCGGTACTGCCGGGCCTCTTGC
GGGATTACGAAATCATCCTGTGGAGCTTAGTAGGTTTAGCAAGATGGCAGCGCC
TAAATGTAGAATGATAAAAGGATTAAGAGATTAATTTCCCTAAAAATGATAAAACA
AGCGTTTTGAAAGCGCTTGTTTTTTTGGTTTGCAGTCAGAGTAGAATAGAAGTAT
CAAAAAAAGCACCGACTCGGTGCCACTTTTTCAAGTTGATAACGGACTAGCCTT
ATTTTAACTTGCTATGCTGTTTTGAATGGTTCCAACAAGATTATTTTATAACTTTTA
TAACAAATAATCAAGGAGAAATTCAAAGAAATTTATCAGCCATAAAACAATACTTA
ATACTATAGAATGATAACAAAATAAACTACTTTTTAAAAGAATTTTGTGTTATAATC
TATTTATTATTAAGTATTGGGTAATATTTTTTGAAGAGCGGCCGCGCACGATCTC
GACACTCGAGATGGATAAGAAATACTCAATAGGCTTAGCTATCGGCACAAATAG
CGTCGGATGGGCGGTGATCACTGATGAATATAAGGTTCCGTCTAAAAAGTTCAA
GGTTCTGGGAAATACAGACCGCCACAGTATCAAAAAAAATCTTATAGGGGCTCT
TTTATTTGACAGTGGAGAGACAGCGGAAGCGACTCGTCTCAAACGGACAGCTC
GTAGAAGGTATACACGTCGGAAGAATCGTATTTGTTATCTACAGGAGATTTTTTC

AAATGAGATGGCGAAAGTAGATGATAGTTTCTTTCATCGACTTGAAGAGTCTTTT
TTGGTGGAAGAAGACAAGAAGCATGAACGTCATCCTATTTTTGGAAATATAGTAG
ATGAAGTTGCTTATCATGAGAAATATCCAACTATCTATCATCTGCGAAAAAAATT
GGTAGATTCTACTGATAAAGCGGATTTGCGCTTAATCTATTTGGCCTTAGCGCAT
ATGATTAAGTTTCGTGGTCATTTTTTGATTGAGGGAGATTTAAATCCTGATAATAG
TGATGTGGACAAACTATTTATCCAGTTGGTACAAACCTACAATCAATTATTTGAA
GAAAACCCTATTAACGCAAGTGGAGTAGATGCTAAAGCGATTCTTTCTGCACGA
TTGAGTAAATCAAGACGATTAGAAAATCTCATTGCTCAGCTCCCCGGTGAGAAG
AAAAATGGCTTATTTGGGAATCTCATTGCTTTGTCATTGGGTTTGACCCCTAATT
TTAAATCAAATTTTGATTTGGCAGAAGATGCTAAATTACAGCTTTCAAAAGATACT
TACGATGATGATTTAGATAATTTATTGGCGCAAATTGGAGATCAATATGCTGATT
TGTTTTTGGCAGCTAAGAATTTATCAGATGCTATTTTACTTTCAGATATCCTAAGA
GTAAATACTGAAATAACTAAGGCTCCCCTATCAGCTTCAATGATTAAACGCTACG
ATGAACATCATCAAGACTTGACTCTTTTAAAAGCTTTAGTTCGACAACAACTTCC
AGAAAAGTATAAAGAAATCTTTTTTGATCAATCAAAAAACGGATATGCAGGTTAT
ATTGATGGGGGAGCTAGCCAAGAAGAATTTTATAAATTTATCAAACCAATTTTAG
AAAAAATGGATGGTACTGAGGAATTATTGGTGAAACTAAATCGTGAAGATTTGCT
GCGCAAGCAACGGACCTTTGACAACGGCTCTATTCCCCATCAAATTCACTTGGG
TGAGCTGCATGCTATTTTGAGAAGACAAGAAGACTTTTATCCATTTTTAAAAGAC
AATCGTGAGAAGATTGAAAAAATCTTGACTTTTCGAATTCCTTATTATGTTGGTCC
ATTGGCGCGTGGCAATAGTCGTTTTGCATGGATGACTCGGAAGTCTGAAGAAAC
AATTACCCCATGGAATTTTGAAGAAGTTGTCGATAAAGGTGCTTCAGCTCAATCA
TTTATTGAACGCATGACAAACTTTGATAAAAATCTTCCAAATGAAAAAGTACTACC
AAAACATAGTTTGCTTTATGAGTATTTTACGGTTTATAACGAATTGACAAAGGTCA
AATATGTTACTGAAGGAATGCGAAAACCAGCATTTCTTTCAGGTGAACAGAAGAA
AGCCATTGTTGATTTACTCTTCAAAACAAATCGAAAAGTAACCGTTAAGCAATTA
AAAGAAGATTATTTCAAAAAAATAGAATGTTTTGATAGTGTTGAAATTTCAGGAGT
TGAAGATAGATTTAATGCTTCATTAGGTACCTACCATGATTTGCTAAAAATTATTA
AAGATAAAGATTTTTTGGATAATGAAGAAAATGAAGATATCTTAGAGGATATTGTT
TTAACATTGACCTTATTTGAAGATAGGGAGATGATTGAGGAAAGACTTAAAACAT
ATGCTCACCTCTTTGATGATAAGGTGATGAAACAGCTTAAACGTCGCCGTTATAC
TGGTTGGGGACGTTTGTCTCGAAAATTGATTAATGGTATTAGGGATAAGCAATCT
GGCAAAACAATATTAGATTTTTTGAAATCAGATGGTTTTGCCAATCGCAATTTTAT
GCAGCTGATCCATGATGATAGTTTGACATTTAAAGAAGACATTCAAAAAGCACAA
GTGTCTGGACAAGGCGATAGTTTACATGAACATATTGCAAATTTAGCTGGTAGC
CCTGCTATTAAAAAAGGTATTTTACAGACTGTAAAAGTTGTTGATGAATTGGTCA
AAGTAATGGGGCGGCATAAGCCAGAAAATATCGTTATTGAAATGGCACGTGAAA
ATCAGACAACTCAAAAGGGCCAGAAAAATTCGCGAGAGCGTATGAAACGAATCG
AAGAAGGTATCAAAGAATTAGGAAGTCAGATTCTTAAAGAGCATCCTGTTGAAAA
TACTCAATTGCAAAATGAAAAGCTCTATCTCTATTATCTCCAAAATGGAAGAGAC
ATGTATGTGGACCAAGAATTAGATATTAATCGTTTAAGTGATTATGATGTCGATG
CCATTGTTCCACAAAGTTTCCTTAAAGACGATTCAATAGACAATAAGGTCTTAAC
GCGTTCTGATAAAAATCGTGGTAAATCGGATAACGTTCCAAGTGAAGAAGTAGT
CAAAAAGATGAAAAACTATTGGAGACAACTTCTAAACGCCAAGTTAATCACTCAA

CGTAAGTTTGATAATTTAACGAAAGCTGAACGTGGAGGTTTGAGTGAACTTGATA
AAGCTGGTTTTATCAAACGCCAATTGGTTGAAACTCGCCAAATCACTAAGCATGT
GGCACAAATTTTGGATAGTCGCATGAATACTAAATACGATGAAAATGATAAACTT
ATTCGAGAGGTTAAAGTGATTACCTTAAAATCTAAATTAGTTTCTGACTTCCGAAA
AGATTTCCAATTCTATAAAGTACGTGAGATTAACAATTACCATCATGCCCATGAT
GCGTATCTAAATGCCGTCGTTGGAACTGCTTTGATTAAGAAATATCCAAAACTTG
AATCGGAGTTTGTCTATGGTGATTATAAAGTTTATGATGTTCGTAAAATGATTGCT
AAGTCTGAGCAAGAAATAGGCAAAGCAACCGCAAAATATTTCTTTTACTCTAATA
TCATGAACTTCTTCAAAACAGAAATTACACTTGCAAATGGAGAGATTCGCAAACG
CCCTCTAATCGAAACTAATGGGGAAACTGGAGAAATTGTCTGGGATAAAGGGCG
AGATTTTGCCACAGTGCGCAAAGTATTGTCCATGCCCCAAGTCAATATTGTCAAG
AAAACAGAAGTACAGACAGGCGGATTCTCCAAGGAGTCAATTTTACCAAAAAGA
AATTCGGACAAGCTTATTGCTCGTAAAAAAGACTGGGATCCAAAAAAATATGGTG
GTTTTGATAGTCCAACGGTAGCTTATTCAGTCCTAGTGGTTGCTAAGGTGGAAAA
AGGGAAATCGAAGAAGTTAAAATCCGTTAAAGAGTTACTAGGGATCACAATTATG
GAAAGAAGTTCCTTTGAAAAAAATCCGATTGACTTTTTAGAAGCTAAAGGATATA
AGGAAGTTAAAAAAGACTTAATCATTAAACTACCTAAATATAGTCTTTTTGAGTTA
GAAAACGGTCGTAAACGGATGCTGGCTAGTGCCGGAGAATTACAAAAAGGAAAT
GAGCTGGCTCTGCCAAGCAAATATGTGAATTTTTTATATTTAGCTAGTCATTATG
AAAAGTTGAAGGGTAGTCCAGAAGATAACGAACAAAAACAATTGTTTGTGGAGC
AGCATAAGCATTATTTAGATGAGATTATTGAGCAAATCAGTGAATTTTCTAAGCG
TGTTATTTTAGCAGATGCCAATTTAGATAAAGTTCTTAGTGCATATAACAAACATA
GAGACAAACCAATACGTGAACAAGCAGAAAATATTATTCATTTATTTACGTTGAC
GAATCTTGGAGCTCCCGCTGCTTTTAAATATTTTGATACAACAATTGATCGTAAA
CGATATACGTCTACAAAAGAAGTTTTAGATGCCACTCTTATCCATCAATCCATCA
CTGGTCTTTATGAAACACGCATTGATTTGAGTCAGCTAGGAGGTCCTAGGGGAG
GTGGCTCAGAAGGTGGCGGATCTGAAGGTGGCTCTGGAactagtATGAGCGATAA
AATTATTCACCTGACTGACGACAGTTTTGACACGGATGTACTCAAAGCGGACGG
GGCGATCCTCGTCGATTTCTGGGCAGAGTGGTGCGGTCCGTGCAAAATGATCG
CCCCGATTCTGGATGAAATCGCTGACGAATATCAGGGCAAACTGACCGTTGCAA
AACTGAACATCGATCAAAACCCTGGCACTGCGCCGAAATATGGCATCCGTGGTA
TCCCGACTCTGCTGCTGTTCAAAAACGGTGAAGTGGCGGCAACCAAAGTGGGT
GCACTGTCTAAAGGTCAGTTGAAAGAGTTCCTCGACGCTAACCTGGCCGGTTCT
GGTTCTGGCCATATGCACCATCATCATCATCATTCTTCTGGTCTGGTGCCACGC
GGTTCTGGTATGAAAGAAACCGCTGCTGCTAAATTCGAACGCCAGCACATGGAC
AGCCCAGATCTGGGTACCGACGACGACGACAAGGCCATGGCTGATATCggatccC
ATATGGAAATGATGCTCGATAAGAAACAGATTCGTGCGATCTTTCTCTTTGAGTT
TAAAATGGGTCGCAAAGCGGCGGAGACGACGCGTAATATTAACAACGCGTTCG
GTCCTGGCACCGCGAACGAGCGTACCGTGCAATGGTGGTTCAAAAAGTTTCGC
AAAGGCGACGAATCTCTGGAGGACGAAGAGCGTTCTGGCCGCCCGTCCGAGGT
TGACAACGACCAGCTGCGTGCAATCATCGAAGCTGATCCGCTGACTACCACCC
GCGAAGTTGCTGAAGAACTGAATGTGGATCACTCTACTGTGGTTCGCCACCTGA
AACAGATCGGTAAAGTAAAAAAACTGGACAAATGGGTTCCTCATGAACTGTCTG
AAAACCAGAAAAACCGTCGTTTCGAAGTTAGCTCCTCTCTGATTCTGCGTAACAA

CAACGAACCGTTCCTGGATCGTATCGTAACCTGTGATGAGAAATGGATTCTGTA
TGATAACCGTCGCCGCTCTGCTCAGTGGCTGGATCGCGAAGAAGCTCCAAAAC
ACTTCCCGAAACCGAATCTGCACCAGAAGAAAGTCATGGTAACCGTATGGTGGT
CTGCCGCAGGTGTTATCCACTATTCCTTCCTGAACCCGGGCGAAACTATCACCA
GCGAAAAATACTGCCAGCAGATTGACGAAATGCACCGTAAACTGCAGCGTCTGC
AGCCAGCACTGGTGAATCGTAAAGGTCCGATCCTGCTGCATGATAACGCCCGTC
CGCACGTTGCCCAACCGACCCTGCAGAAACTGAACGAACTGGGCTATGAAGTT
CTGCCACACCCGCCGTACTCCCCGGATCTGTCCCCGACTGACTACCATTTCTTC
AAGCATCTGGACAACTTCCTGCAGGGTAAACGTTTTCACAACCAACAGGACGCA
GAAAACGCTTTCCAGGAGTTCGTCGAAAGCCGTTCCACTGACTTCTACGCGACC
GGTATCAACAAGCTGATCAGCCGTTGGCAGAAATGCGTGGACTGTAACGGCAG
CTACTTCGATTAAGACGTCTTAATAACTAAAAATATGGTATAATACTCTTAATAAA
TGCAGTAATACAGGGGCTTTTCAAGACTGAAGTCTAGCTGAGACAAATAGTGCG
ATTACGAAATTTTTTAGACAAAAATAGTCTACGAGGTTTTAGAGCTATGCTGTTTT
GAATGGTCCCAAAACGGTTTTCCCAGTCACGACGTTGTAAAACGAGTTTTAGAG
CTATGCTGTTTTGAATGGTCCCAAAACTTCAGCACACTGAGACTTGTTGAGTTCC
ATGTTTTAGAGCTATGCTGTTTTGAATGGACTCCATTCAACATTGCCGATGATAA
CTTGAGAAAGAGGGTTAATACCAGCAGTCGGATACCTTCCTATTCTTTCTGTTAA
AGCGTTTTCATGTTATAATAGGCAAAAGAAGAGTAGTGTGATCGTCCATTCCGAC
AGCATCGCCAGTCACTATGGCGTGCTGCTAGCGCTATATGCGTTGATGCAATTT
CTATGCGCACCCGTTCTCGGAGCACTGTCCGACCGCTTTGGCCGCCGCCCAGT
CCTGCTCGCTTCGCTACTTGGAGCCACTATCGACTACGCGATCATGGCGACCAC
ACCCGTCCTGTGGATCCTCTACGCCGGACGCATCGTGGCCGGCATCACCGGCG
CCACAGGTGCGGTTGCTGGCGCCTATATCGCCGACATCACCGATGGGGAAGAT
CGGGCTCGCCACTTCGGGCTCATGAGCGCTTGTTTCGGCGTGGGTATGGTGGC
AGGCCCCGTGGCCGGGGGACTGTTGGGCGCCATCTCCTTGCATGCACCATTCC
TTGCGGCGGCGGTGCTCAACGGCCTCAACCTACTACTGGGCTGCTTCCTAATG
CAGGAGTCGCATAAGGGAGAGCGTCGACCGATGCCCTTGAGAGCCTTCAACCC
AGTCAGCTCCTTCCGGTGGGCGCGGGGCATGACTATCGTCGCCGCACTTATGA
CTGTCTTCTTTATCATGCAACTCGTAGGACAGGTGCCGGCAGCGCTCTGGGTCA
TTTTCGGCGAGGACCGCTTTCGCTGGAGCGCGACGATGATCGGCCTGTCGCTT
GCGGTATTCGGAATCTTGCACGCCCTCGCTCAAGCCTTCGTCACTGGTCCCGC
CACCAAACGTTTCGGCGAGAAGCAGGCCATTATCGCCGGCATGGCGGCCGACG
CGCTGGGCTACGTCTTGCTGGCGTTCGCGACGCGAGGCTGGATGGCCTTCCCC
ATTATGATTCTTCTCGCTTCCGGCGGCATCGGGATGCCCGCGTTGCAGGCCATG
CTGTCCAGGCAGGTAGATGACGACCATCAGGGACAGCTTCAAGGATCGCTCGC
GGCTCTTACCAGCCTAACTTCGATCATTGGACCGCTGATCGTCACGGCGATTTA
TGCCGCCTCGGCGAGCACATGGAACGGGTTGGCATGGATTGTAGGCGCCGCC
CTATACCTTGTCTGCCTCCCCGCGTTGCGTCGCGGTGCATGGAGCCGGGCCAC
CTCGACCTGAATGGAAGCCGGCGGCACCTCGCTAACGGATTCACCACTCCAAG
AATTGGAGCCAATCAATTCTTGCGGAGAACTGTGAATGCGCAAACCAACCCTTG
GCAGAACATATCCATCGCGTCCGCCATCTCCAGCAGCCGCACGCGGCGCATCT
CGGGCAGCGTTGGGTCCTGGCCACGGGTGCGCATGATCGTGCTCCTGTCGTTG
AGGACCCGGCTAGGCTGGCGGGGTTGCCTTACTGGTTAGCAGAATGAATCACC

GATACGCGAGCGAACGTGAAGCGACTGCTGCTGCAAAACGTCTGCGACCTGAG
CAACAACATGAATGGTCTTCGGTTTCCGTGTTTCGTAAAGTCTGGAAACGCGGA
AGTCCCCTACGTGCTGCTGAAGTTGCCCGCAACAGAGAGTGGAACCAACCGGT
GATACCACGATACTATGACTGAGAGTCAACGCCATGAGCGGCCTCATTTCTTAT
TCTGAGTTACAACAGTCCGCACCGCTGTCCGGTAGCTCCTTCCGGTGGGCGCG
GGGCATGACTATCGTCGCCGCACTTATGACTGTCTTCTTTATCATGCAACTCGTA
GGACAGGTGCCGGCAGCGCCCAACAGTCCCCCGGCCACGGGGCCTGCCACCA
TACCCACGCCGAAACAAGCGCCCTGCACCATTATGTTCCGGATCTGCATCGCAG
GATGCTGCTGGCTACCCTGTGGAACACCTACATCTGTATTAACGAAGCGCTAAC
CGTTTTTATCAGGCTCTGGGAGGCAGAATAAATGATCATATCGTCAATTATTACC
TCCACGGGGAGAGCCTGAGCAAACTGGCCTCAGGCATTTGAGAAGCACACGGT
CACACTGCTTCCGGTAGTCAATAAACCGGTAAACCAGCAATAGACATAAGCGGC
TATTTAACGACCCTGCCCTGAACCGACGACCGGGTCGAATTTGCTTTCGAATTT
CTGCCATTCATCCGCTTATTATCACTTATTCAGGCGTAGCACCAGGCGTTTAAGG
GCACCAATAACTGCCTTAAAAAAATTACGCCCCGCCCTGCCACTCATCGCAGTA
CTGTTGTAATTCATTAAGCATTCTGCCGACATGGAAGCCATCACAGACGGCATG
ATGAACCTGAATCGCCAGCGGCATCAGCACCTTGTCGCCTTGCGTATAATATTT
GCCCATGGTGAAAACGGGGGCGAAGAAGTTGTCCATATTGGCCACGTTTAAATC
AAAACTGGTGAAACTCACCCAGGGATTGGCTGAGACGAAAAACATATTCTCAAT
AAACCCTTTAGGGAAATAGGCCAGGTTTTCACCGTAACACGCCACATCTTGCGA
ATATATGTGTAGAAACTGCCGGAAATCGTCGTGGTATTCACTCCAGAGCGATGA
AAACGTTTCAGTTTGCTCATGGAAAACGGTGTAACAAGGGTGAACACTATCCCA
TATCACCAGCTCACCGTCTTTCATTGCCATACG

pRC2308

GAATTCCGGATGAGCATTCATCAGGCGGGCAAGAATGTGAATAAAGGCCGGAT
AAAACTTGTGCTTATTTTTCTTTACGGTCTTTAAAAAGGCCGTAATATCCAGCTGA
ACGGTCTGGTTATAGGTACATTGAGCAACTGACTGAAATGCCTCAAAATGTTCTT
TACGATGCCATTGGGATATATCAACGGTGGTATATCCAGTGATTTTTTTCTCCAT
TTTAGCTTCCTTAGCTCCTGAAAATCTCGATAACTCAAAAAATACGCCCGGTAGT
GATCTTATTTCATTATGGTGAAAGTTGGAACCTCTTACGTGCCGATCAACGTCTC
ATTTTCGCCAAAAGTTGGCCCAGGGCTTCCCGGTATCAACAGGGACACCAGGAT
TTATTTATTCTGCGAAGTGATCTTCCGTCACAGGTATTTATTCGGCGCAAAGTGC
GTCGGGTGATGCTGCCAACTTACTGATTTAGTGTATGATGGTGTTTTTGAGGTG
CTCCAGTGGCTTCTGTTTCTATCAGCTGTCCCTCCTGTTCAGCTACTGACGGGG
TGGTGCGTAACGGCAAAAGCACCGCCGGACATCAGCGCTAGCGGAGTGTATAC
TGGCTTACTATGTTGGCACTGATGAGGGTGTCAGTGAAGTGCTTCATGTGGCAG
GAGAAAAAAGGCTGCACCGGTGCGTCAGCAGAATATGTGATACAGGATATATTC
CGCTTCCTCGCTCACTGACTCGCTACGCTCGGTCGTTCGACTGCGGCGAGCGG
AAATGGCTTACGAACGGGGCGGAGATTTCCTGGAAGATGCCAGGAAGATACTTA
ACAGGGAAGTGAGAGGGCCGCGGCAAAGCCGTTTTTCCATAGGCTCCGCCCCC
CTGACAAGCATCACGAAATCTGACGCTCAAATCAGTGGTGGCGAAACCCGACA
GGACTATAAAGATACCAGGCGTTTCCCCCTGGCGGCTCCCTCGTGCGCTCTCCT

GTTCCTGCCTTTCGGTTTACCGGTGTCATTCCGCTGTTATGGCCGCGTTTGTCT
CATTCCACGCCTGACACTCAGTTCCGGGTAGGCAGTTCGCTCCAAGCTGGACT
GTATGCACGAACCCCCCGTTCAGTCCGACCGCTGCGCCTTATCCGGTAACTATC
GTCTTGAGTCCAACCCGGAAAGACATGCAAAAGCACCACTGGCAGCAGCCACT
GGTAATTGATTTAGAGGAGTTAGTCTTGAAGTCATGCGCCGGTTAAGGCTAAAC
TGAAAGGACAAGTTTTGGTGACTGCGCTCCTCCAAGCCAGTTACCTCGGTTCAA
AGAGTTGGTAGCTCAGAGAACCTTCGAAAAACCGCCCTGCAAGGCGGTTTTTTC
GTTTTCAGAGCAAGAGATTACGCGCAGACCAAAACGATCTCAAGAAGATCATCT
TATTAATCAGATAAAATATTTCTAGATTTCAGTGCAATTTATCTCTTCAAATGTAG
CACCTGAAGTCAGCCCCATACGATATAAGTTGTAATTCTCATGTTTGACAGCTTA
TCATCGATAAGCTTTAATGCGGTAGTTTATCACAGTTAAATTGCTAACGCAGTCA
GGCACCGTGTATGAAATCTAACAATGCGCTCATCGTCATCCTCGGCACCGTCAC
CCTGGATGCTGTAGGCATAGGCTTGGTTATGCCGGTACTGCCGGGCCTCTTGC
GGGATTACGAAATCATCCTGTGGAGCTTAGTAGGTTTAGCAAGATGGCAGCGCC
TAAATGTAGAATGATAAAAGGATTAAGAGATTAATTTCCCTAAAAATGATAAAACA
AGCGTTTTGAAAGCGCTTGTTTTTTTGGTTTGCAGTCAGAGTAGAATAGAAGTAT
CAAAAAAAGCACCGACTCGGTGCCACTTTTTCAAGTTGATAACGGACTAGCCTT
ATTTTAACTTGCTATGCTGTTTTGAATGGTTCCAACAAGATTATTTTATAACTTTTA
TAACAAATAATCAAGGAGAAATTCAAAGAAATTTATCAGCCATAAAACAATACTTA
ATACTATAGAATGATAACAAAATAAACTACTTTTTAAAAGAATTTTGTGTTATAATC
TATTTATTATTAAGTATTGGGTAATATTTTTTGAAGAGCGGCCGCGTTAATAACAG
GGGACGTGGTAATCCGTCCCCTTTTTATTTCTGACTGAGTTAATAACAGGCCTG
CTTCGGCAGGCCTTTTTATTTCTGACTGAGTTCTTCTCAGGCCTGCTGGTAATCG
CAGGCCTTTTTATTTTCTAGTCTGACTGACTGACTGACTGACTGACTGACTGACT
GACTGACTGACTGACTGACTGACTGACTGACTGACTGACTGACTGACTGACTGA
CTGACTGACTGACTGACTGACTGACTGACTGACTGACTGACTGACTGACTGACT
GACTGACTGACTGACTGACTGACTGACTgactgaCTGACCTCGAGATGGATAAGAA
ATACTCAATAGGCTTAGCTATCGGCACAAATAGCGTCGGATGGGCGGTGATCAC
TGATGAATATAAGGTTCCGTCTAAAAAGTTCAAGGTTCTGGGAAATACAGACCG
CCACAGTATCAAAAAAAATCTTATAGGGGCTCTTTTATTTGACAGTGGAGAGACA
GCGGAAGCGACTCGTCTCAAACGGACAGCTCGTAGAAGGTATACACGTCGGAA
GAATCGTATTTGTTATCTACAGGAGATTTTTTCAAATGAGATGGCGAAAGTAGAT
GATAGTTTCTTTCATCGACTTGAAGAGTCTTTTTTGGTGGAAGAAGACAAGAAGC
ATGAACGTCATCCTATTTTTGGAAATATAGTAGATGAAGTTGCTTATCATGAGAA
ATATCCAACTATCTATCATCTGCGAAAAAAATTGGTAGATTCTACTGATAAAGCG
GATTTGCGCTTAATCTATTTGGCCTTAGCGCATATGATTAAGTTTCGTGGTCATT
TTTTGATTGAGGGAGATTTAAATCCTGATAATAGTGATGTGGACAAACTATTTAT
CCAGTTGGTACAAACCTACAATCAATTATTTGAAGAAAACCCTATTAACGCAAGT
GGAGTAGATGCTAAAGCGATTCTTTCTGCACGATTGAGTAAATCAAGACGATTA
GAAAATCTCATTGCTCAGCTCCCCGGTGAGAAGAAAAATGGCTTATTTGGGAAT
CTCATTGCTTTGTCATTGGGTTTGACCCCTAATTTTAAATCAAATTTTGATTTGGC
AGAAGATGCTAAATTACAGCTTTCAAAAGATACTTACGATGATGATTTAGATAATT
TATTGGCGCAAATTGGAGATCAATATGCTGATTTGTTTTTGGCAGCTAAGAATTT
ATCAGATGCTATTTTACTTTCAGATATCCTAAGAGTAAATACTGAAATAACTAAGG

CTCCCCTATCAGCTTCAATGATTAAACGCTACGATGAACATCATCAAGACTTGAC
TCTTTTAAAAGCTTTAGTTCGACAACAACTTCCAGAAAAGTATAAAGAAATCTTTT
TTGATCAATCAAAAAACGGATATGCAGGTTATATTGATGGGGGAGCTAGCCAAG
AAGAATTTTATAAATTTATCAAACCAATTTTAGAAAAAATGGATGGTACTGAGGAA
TTATTGGTGAAACTAAATCGTGAAGATTTGCTGCGCAAGCAACGGACCTTTGAC
AACGGCTCTATTCCCCATCAAATTCACTTGGGTGAGCTGCATGCTATTTTGAGAA
GACAAGAAGACTTTTATCCATTTTTAAAAGACAATCGTGAGAAGATTGAAAAAAT
CTTGACTTTTCGAATTCCTTATTATGTTGGTCCATTGGCGCGTGGCAATAGTCGT
TTTGCATGGATGACTCGGAAGTCTGAAGAAACAATTACCCCATGGAATTTTGAAG
AAGTTGTCGATAAAGGTGCTTCAGCTCAATCATTTATTGAACGCATGACAAACTT
TGATAAAAATCTTCCAAATGAAAAAGTACTACCAAAACATAGTTTGCTTTATGAGT
ATTTTACGGTTTATAACGAATTGACAAAGGTCAAATATGTTACTGAAGGAATGCG
AAAACCAGCATTTCTTTCAGGTGAACAGAAGAAAGCCATTGTTGATTTACTCTTC
AAAACAAATCGAAAAGTAACCGTTAAGCAATTAAAAGAAGATTATTTCAAAAAAAT
AGAATGTTTTGATAGTGTTGAAATTTCAGGAGTTGAAGATAGATTTAATGCTTCAT
TAGGTACCTACCATGATTTGCTAAAAATTATTAAAGATAAAGATTTTTTGGATAAT
GAAGAAAATGAAGATATCTTAGAGGATATTGTTTTAACATTGACCTTATTTGAAGA
TAGGGAGATGATTGAGGAAAGACTTAAAACATATGCTCACCTCTTTGATGATAAG
GTGATGAAACAGCTTAAACGTCGCCGTTATACTGGTTGGGGACGTTTGTCTCGA
AAATTGATTAATGGTATTAGGGATAAGCAATCTGGCAAAACAATATTAGATTTTTT
GAAATCAGATGGTTTTGCCAATCGCAATTTTATGCAGCTGATCCATGATGATAGT
TTGACATTTAAAGAAGACATTCAAAAAGCACAAGTGTCTGGACAAGGCGATAGTT
TACATGAACATATTGCAAATTTAGCTGGTAGCCCTGCTATTAAAAAAGGTATTTTA
CAGACTGTAAAAGTTGTTGATGAATTGGTCAAAGTAATGGGGCGGCATAAGCCA
GAAAATATCGTTATTGAAATGGCACGTGAAAATCAGACAACTCAAAAGGGCCAG
AAAAATTCGCGAGAGCGTATGAAACGAATCGAAGAAGGTATCAAAGAATTAGGA
AGTCAGATTCTTAAAGAGCATCCTGTTGAAAATACTCAATTGCAAAATGAAAAGC
TCTATCTCTATTATCTCCAAAATGGAAGAGACATGTATGTGGACCAAGAATTAGA
TATTAATCGTTTAAGTGATTATGATGTCGATGCCATTGTTCCACAAAGTTTCCTTA
AAGACGATTCAATAGACAATAAGGTCTTAACGCGTTCTGATAAAAATCGTGGTAA
ATCGGATAACGTTCCAAGTGAAGAAGTAGTCAAAAAGATGAAAAACTATTGGAG
ACAACTTCTAAACGCCAAGTTAATCACTCAACGTAAGTTTGATAATTTAACGAAA
GCTGAACGTGGAGGTTTGAGTGAACTTGATAAAGCTGGTTTTATCAAACGCCAA
TTGGTTGAAACTCGCCAAATCACTAAGCATGTGGCACAAATTTTGGATAGTCGCA
TGAATACTAAATACGATGAAAATGATAAACTTATTCGAGAGGTTAAAGTGATTAC
CTTAAAATCTAAATTAGTTTCTGACTTCCGAAAAGATTTCCAATTCTATAAAGTAC
GTGAGATTAACAATTACCATCATGCCCATGATGCGTATCTAAATGCCGTCGTTGG
AACTGCTTTGATTAAGAAATATCCAAAACTTGAATCGGAGTTTGTCTATGGTGAT
TATAAAGTTTATGATGTTCGTAAAATGATTGCTAAGTCTGAGCAAGAAATAGGCA
AAGCAACCGCAAAATATTTCTTTTACTCTAATATCATGAACTTCTTCAAAACAGAA
ATTACACTTGCAAATGGAGAGATTCGCAAACGCCCTCTAATCGAAACTAATGGG
GAAACTGGAGAAATTGTCTGGGATAAAGGGCGAGATTTTGCCACAGTGCGCAAA
GTATTGTCCATGCCCCAAGTCAATATTGTCAAGAAAACAGAAGTACAGACAGGC
GGATTCTCCAAGGAGTCAATTTTACCAAAAAGAAATTCGGACAAGCTTATTGCTC

GTAAAAAAGACTGGGATCCAAAAAAATATGGTGGTTTTGATAGTCCAACGGTAG
CTTATTCAGTCCTAGTGGTTGCTAAGGTGGAAAAAGGGAAATCGAAGAAGTTAA
AATCCGTTAAAGAGTTACTAGGGATCACAATTATGGAAAGAAGTTCCTTTGAAAA
AAATCCGATTGACTTTTTAGAAGCTAAAGGATATAAGGAAGTTAAAAAAGACTTA
ATCATTAAACTACCTAAATATAGTCTTTTTGAGTTAGAAAACGGTCGTAAACGGA
TGCTGGCTAGTGCCGGAGAATTACAAAAAGGAAATGAGCTGGCTCTGCCAAGC
AAATATGTGAATTTTTTATATTTAGCTAGTCATTATGAAAAGTTGAAGGGTAGTCC
AGAAGATAACGAACAAAAACAATTGTTTGTGGAGCAGCATAAGCATTATTTAGAT
GAGATTATTGAGCAAATCAGTGAATTTTCTAAGCGTGTTATTTTAGCAGATGCCA
ATTTAGATAAAGTTCTTAGTGCATATAACAAACATAGAGACAAACCAATACGTGA
ACAAGCAGAAAATATTATTCATTTATTTACGTTGACGAATCTTGGAGCTCCCGCT
GCTTTTAAATATTTTGATACAACAATTGATCGTAAACGATATACGTCTACAAAAGA
AGTTTTAGATGCCACTCTTATCCATCAATCCATCACTGGTCTTTATGAAACACGC
ATTGATTTGAGTCAGCTAGGAGGTCCTAGGGGAGGTGGCTCAGAAGGTGGCGG
ATCTGAAGGTGGCTCTGGAactagtATGAGCGATAAAATTATTCACCTGACTGACG
ACAGTTTTGACACGGATGTACTCAAAGCGGACGGGGCGATCCTCGTCGATTTCT
GGGCAGAGTGGTGCGGTCCGTGCAAAATGATCGCCCCGATTCTGGATGAAATC
GCTGACGAATATCAGGGCAAACTGACCGTTGCAAAACTGAACATCGATCAAAAC
CCTGGCACTGCGCCGAAATATGGCATCCGTGGTATCCCGACTCTGCTGCTGTTC
AAAAACGGTGAAGTGGCGGCAACCAAAGTGGGTGCACTGTCTAAAGGTCAGTT
GAAAGAGTTCCTCGACGCTAACCTGGCCGGTTCTGGTTCTGGCCATATGCACCA
TCATCATCATCATTCTTCTGGTCTGGTGCCACGCGGTTCTGGTATGAAAGAAAC
CGCTGCTGCTAAATTCGAACGCCAGCACATGGACAGCCCAGATCTGGGTACCG
ACGACGACGACAAGGCCATGGCTGATATCggatccCATATGGAAATGATGCTCGAT
AAGAAACAGATTCGTGCGATCTTTCTCTTTGAGTTTAAAATGGGTCGCAAAGCG
GCGGAGACGACGCGTAATATTAACAACGCGTTCGGTCCTGGCACCGCGAACGA
GCGTACCGTGCAATGGTGGTTCAAAAAGTTTCGCAAAGGCGACGAATCTCTGGA
GGACGAAGAGCGTTCTGGCCGCCCGTCCGAGGTTGACAACGACCAGCTGCGTG
CAATCATCGAAGCTGATCCGCTGACTACCACCCGCGAAGTTGCTGAAGAACTGA
ATGTGGATCACTCTACTGTGGTTCGCCACCTGAAACAGATCGGTAAAGTAAAAA
AACTGGACAAATGGGTTCCTCATGAACTGTCTGAAAACCAGAAAAACCGTCGTT
TCGAAGTTAGCTCCTCTCTGATTCTGCGTAACAACAACGAACCGTTCCTGGATC
GTATCGTAACCTGTGATGAGAAATGGATTCTGTATGATAACCGTCGCCGCTCTG
CTCAGTGGCTGGATCGCGAAGAAGCTCCAAAACACTTCCCGAAACCGAATCTGC
ACCAGAAGAAAGTCATGGTAACCGTATGGTGGTCTGCCGCAGGTGTTATCCACT
ATTCCTTCCTGAACCCGGGCGAAACTATCACCAGCGAAAAATACTGCCAGCAGA
TTGACGAAATGCACCGTAAACTGCAGCGTCTGCAGCCAGCACTGGTGAATCGTA
AAGGTCCGATCCTGCTGCATGATAACGCCCGTCCGCACGTTGCCCAACCGACC
CTGCAGAAACTGAACGAACTGGGCTATGAAGTTCTGCCACACCCGCCGTACTCC
CCGGATCTGTCCCCGACTGACTACCATTTCTTCAAGCATCTGGACAACTTCCTG
CAGGGTAAACGTTTTCACAACCAACAGGACGCAGAAAACGCTTTCCAGGAGTTC
GTCGAAAGCCGTTCCACTGACTTCTACGCGACCGGTATCAACAAGCTGATCAGC
CGTTGGCAGAAATGCGTGGACTGTAACGGCAGCTACTTCGATTAAGACGTCTTA
ATAACTAAAAATATGGTATAATACTCTTAATAAATGCAGTAATACAGGGGCTTTTC

AAGACTGAAGTCTAGCTGAGACAAATAGTGCGATTACGAAATTTTTTAGACAAAA
ATAGTCTACGAGGTTTTAGAGCTATGCTGTTTTGAATGGTCCCAAAACGGTTTTC
CCAGTCACGACGTTGTAAAACGAGTTTTAGAGCTATGCTGTTTTGAATGGTCCCA
AAACTTCAGCACACTGAGACTTGTTGAGTTCCATGTTTTAGAGCTATGCTGTTTT
GAATGGACTCCATTCAACATTGCCGATGATAACTTGAGAAAGAGGGTTAATACC
AGCAGTCGGATACCTTCCTATTCTTTCTGTTAAAGCGTTTTCATGTTATAATAGG
CAAAAGAAGAGTAGTGTGATCGTCCATTCCGACAGCATCGCCAGTCACTATGGC
GTGCTGCTAGCGCTATATGCGTTGATGCAATTTCTATGCGCACCCGTTCTCGGA
GCACTGTCCGACCGCTTTGGCCGCCGCCCAGTCCTGCTCGCTTCGCTACTTGG
AGCCACTATCGACTACGCGATCATGGCGACCACACCCGTCCTGTGGATCCTCTA
CGCCGGACGCATCGTGGCCGGCATCACCGGCGCCACAGGTGCGGTTGCTGGC
GCCTATATCGCCGACATCACCGATGGGGAAGATCGGGCTCGCCACTTCGGGCT
CATGAGCGCTTGTTTCGGCGTGGGTATGGTGGCAGGCCCCGTGGCCGGGGGA
CTGTTGGGCGCCATCTCCTTGCATGCACCATTCCTTGCGGCGGCGGTGCTCAA
CGGCCTCAACCTACTACTGGGCTGCTTCCTAATGCAGGAGTCGCATAAGGGAG
AGCGTCGACCGATGCCCTTGAGAGCCTTCAACCCAGTCAGCTCCTTCCGGTGG
GCGCGGGGCATGACTATCGTCGCCGCACTTATGACTGTCTTCTTTATCATGCAA
CTCGTAGGACAGGTGCCGGCAGCGCTCTGGGTCATTTTCGGCGAGGACCGCTT
TCGCTGGAGCGCGACGATGATCGGCCTGTCGCTTGCGGTATTCGGAATCTTGC
ACGCCCTCGCTCAAGCCTTCGTCACTGGTCCCGCCACCAAACGTTTCGGCGAG
AAGCAGGCCATTATCGCCGGCATGGCGGCCGACGCGCTGGGCTACGTCTTGCT
GGCGTTCGCGACGCGAGGCTGGATGGCCTTCCCCATTATGATTCTTCTCGCTTC
CGGCGGCATCGGGATGCCCGCGTTGCAGGCCATGCTGTCCAGGCAGGTAGAT
GACGACCATCAGGGACAGCTTCAAGGATCGCTCGCGGCTCTTACCAGCCTAAC
TTCGATCATTGGACCGCTGATCGTCACGGCGATTTATGCCGCCTCGGCGAGCA
CATGGAACGGGTTGGCATGGATTGTAGGCGCCGCCCTATACCTTGTCTGCCTC
CCCGCGTTGCGTCGCGGTGCATGGAGCCGGGCCACCTCGACCTGAATGGAAG
CCGGCGGCACCTCGCTAACGGATTCACCACTCCAAGAATTGGAGCCAATCAATT
CTTGCGGAGAACTGTGAATGCGCAAACCAACCCTTGGCAGAACATATCCATCGC
GTCCGCCATCTCCAGCAGCCGCACGCGGCGCATCTCGGGCAGCGTTGGGTCCT
GGCCACGGGTGCGCATGATCGTGCTCCTGTCGTTGAGGACCCGGCTAGGCTGG
CGGGGTTGCCTTACTGGTTAGCAGAATGAATCACCGATACGCGAGCGAACGTG
AAGCGACTGCTGCTGCAAAACGTCTGCGACCTGAGCAACAACATGAATGGTCTT
CGGTTTCCGTGTTTCGTAAAGTCTGGAAACGCGGAAGTCCCCTACGTGCTGCTG
AAGTTGCCCGCAACAGAGAGTGGAACCAACCGGTGATACCACGATACTATGACT
GAGAGTCAACGCCATGAGCGGCCTCATTTCTTATTCTGAGTTACAACAGTCCGC
ACCGCTGTCCGGTAGCTCCTTCCGGTGGGCGCGGGGCATGACTATCGTCGCCG
CACTTATGACTGTCTTCTTTATCATGCAACTCGTAGGACAGGTGCCGGCAGCGC
CCAACAGTCCCCCGGCCACGGGGCCTGCCACCATACCCACGCCGAAACAAGC
GCCCTGCACCATTATGTTCCGGATCTGCATCGCAGGATGCTGCTGGCTACCCTG
TGGAACACCTACATCTGTATTAACGAAGCGCTAACCGTTTTTATCAGGCTCTGG
GAGGCAGAATAAATGATCATATCGTCAATTATTACCTCCACGGGGAGAGCCTGA
GCAAACTGGCCTCAGGCATTTGAGAAGCACACGGTCACACTGCTTCCGGTAGTC
AATAAACCGGTAAACCAGCAATAGACATAAGCGGCTATTTAACGACCCTGCCCT

GAACCGACGACCGGGTCGAATTTGCTTTCGAATTTCTGCCATTCATCCGCTTATT
ATCACTTATTCAGGCGTAGCACCAGGCGTTTAAGGGCACCAATAACTGCCTTAA
AAAAATTACGCCCCGCCCTGCCACTCATCGCAGTACTGTTGTAATTCATTAAGCA
TTCTGCCGACATGGAAGCCATCACAGACGGCATGATGAACCTGAATCGCCAGC
GGCATCAGCACCTTGTCGCCTTGCGTATAATATTTGCCCATGGTGAAAACGGGG
GCGAAGAAGTTGTCCATATTGGCCACGTTTAAATCAAAACTGGTGAAACTCACC
CAGGGATTGGCTGAGACGAAAAACATATTCTCAATAAACCCTTTAGGGAAATAG
GCCAGGTTTTCACCGTAACACGCCACATCTTGCGAATATATGTGTAGAAACTGC
CGGAAATCGTCGTGGTATTCACTCCAGAGCGATGAAAACGTTTCAGTTTGCTCA
TGGAAAACGGTGTAACAAGGGTGAACACTATCCCATATCACCAGCTCACCGTCT
TTCATTGCCATACG

pRC2309

GAATTCCGGATGAGCATTCATCAGGCGGGCAAGAATGTGAATAAAGGCCGGAT
AAAACTTGTGCTTATTTTTCTTTACGGTCTTTAAAAAGGCCGTAATATCCAGCTGA
ACGGTCTGGTTATAGGTACATTGAGCAACTGACTGAAATGCCTCAAAATGTTCTT
TACGATGCCATTGGGATATATCAACGGTGGTATATCCAGTGATTTTTTTCTCCAT
TTTAGCTTCCTTAGCTCCTGAAAATCTCGATAACTCAAAAAATACGCCCGGTAGT
GATCTTATTTCATTATGGTGAAAGTTGGAACCTCTTACGTGCCGATCAACGTCTC
ATTTTCGCCAAAAGTTGGCCCAGGGCTTCCCGGTATCAACAGGGACACCAGGAT
TTATTTATTCTGCGAAGTGATCTTCCGTCACAGGTATTTATTCGGCGCAAAGTGC
GTCGGGTGATGCTGCCAACTTACTGATTTAGTGTATGATGGTGTTTTTGAGGTG
CTCCAGTGGCTTCTGTTTCTATCAGCTGTCCCTCCTGTTCAGCTACTGACGGGG
TGGTGCGTAACGGCAAAAGCACCGCCGGACATCAGCGCTAGCGGAGTGTATAC
TGGCTTACTATGTTGGCACTGATGAGGGTGTCAGTGAAGTGCTTCATGTGGCAG
GAGAAAAAAGGCTGCACCGGTGCGTCAGCAGAATATGTGATACAGGATATATTC
CGCTTCCTCGCTCACTGACTCGCTACGCTCGGTCGTTCGACTGCGGCGAGCGG
AAATGGCTTACGAACGGGGCGGAGATTTCCTGGAAGATGCCAGGAAGATACTTA
ACAGGGAAGTGAGAGGGCCGCGGCAAAGCCGTTTTTCCATAGGCTCCGCCCCC
CTGACAAGCATCACGAAATCTGACGCTCAAATCAGTGGTGGCGAAACCCGACA
GGACTATAAAGATACCAGGCGTTTCCCCCTGGCGGCTCCCTCGTGCGCTCTCCT
GTTCCTGCCTTTCGGTTTACCGGTGTCATTCCGCTGTTATGGCCGCGTTTGTCT
CATTCCACGCCTGACACTCAGTTCCGGGTAGGCAGTTCGCTCCAAGCTGGACT
GTATGCACGAACCCCCCGTTCAGTCCGACCGCTGCGCCTTATCCGGTAACTATC
GTCTTGAGTCCAACCCGGAAAGACATGCAAAAGCACCACTGGCAGCAGCCACT
GGTAATTGATTTAGAGGAGTTAGTCTTGAAGTCATGCGCCGGTTAAGGCTAAAC
TGAAAGGACAAGTTTTGGTGACTGCGCTCCTCCAAGCCAGTTACCTCGGTTCAA
AGAGTTGGTAGCTCAGAGAACCTTCGAAAAACCGCCCTGCAAGGCGGTTTTTTC
GTTTTCAGAGCAAGAGATTACGCGCAGACCAAAACGATCTCAAGAAGATCATCT
TATTAATCAGATAAAATATTTCTAGATTTCAGTGCAATTTATCTCTTCAAATGTAG
CACCTGAAGTCAGCCCCATACGATATAAGTTGTAATTCTCATGTTTGACAGCTTA
TCATCGATAAGCTTTAATGCGGTAGTTTATCACAGTTAAATTGCTAACGCAGTCA
GGCACCGTGTATGAAATCTAACAATGCGCTCATCGTCATCCTCGGCACCGTCAC

CCTGGATGCTGTAGGCATAGGCTTGGTTATGCCGGTACTGCCGGGCCTCTTGC
GGGATTACGAAATCATCCTGTGGAGCTTAGTAGGTTTAGCAAGATGGCAGCGCC
TAAATGTAGAATGATAAAAGGATTAAGAGATTAATTTCCCTAAAAATGATAAAACA
AGCGTTTTGAAAGCGCTTGTTTTTTTGGTTTGCAGTCAGAGTAGAATAGAAGTAT
CAAAAAAAGCACCGACTCGGTGCCACTTTTTCAAGTTGATAACGGACTAGCCTT
ATTTTAACTTGCTATGCTGTTTTGAATGGTTCCAACAAGATTATTTTATAACTTTTA
TAACAAATAATCAAGGAGAAATTCAAAGAAATTTATCAGCCATAAAACAATACTTA
ATACTATAGAATGATAACAAAATAAACTACTTTTTAAAAGAATTTTGTGTTATAATC
TATTTATTATTAAGTATTGGGTAATATTTTTTGAAGAGCggccgctaatacgactcactatag
ggagaggatccgcggaattgCAGCTGGTTAATAACAGGGGACGTGGTAATCCGTCCCCT
TTTTATTTCTGACTGAGTTAATAACAGGCCTGCTTCGGCAGGCCTTTTTATTTCT
GACTGAGTTCTTCTCAGGCCTGCTGGTAATCGCAGGCCTTTTTATTTTCTAGACA
ATTCCGACGTCTAAGGAAACCATTATCATGACATCAACCTATAAAAATAGGCGTA
TCACGAGGCCCTCTCGTCTCCACCTCAAGCTCCCTATCTAGTGATAGCGATTGA
CATCCCTATCAGTGACGGAGATATTGAGCACATCAGCAGGACGCACTGACCACT
TTAAGaggaggCTCGAGATGGATAAGAAATACTCAATAGGCTTAGCTATCGGCACA
AATAGCGTCGGATGGGCGGTGATCACTGATGAATATAAGGTTCCGTCTAAAAAG
TTCAAGGTTCTGGGAAATACAGACCGCCACAGTATCAAAAAAAATCTTATAGGG
GCTCTTTTATTTGACAGTGGAGAGACAGCGGAAGCGACTCGTCTCAAACGGACA
GCTCGTAGAAGGTATACACGTCGGAAGAATCGTATTTGTTATCTACAGGAGATTT
TTTCAAATGAGATGGCGAAAGTAGATGATAGTTTCTTTCATCGACTTGAAGAGTC
TTTTTTGGTGGAAGAAGACAAGAAGCATGAACGTCATCCTATTTTTGGAAATATA
GTAGATGAAGTTGCTTATCATGAGAAATATCCAACTATCTATCATCTGCGAAAAA
AATTGGTAGATTCTACTGATAAAGCGGATTTGCGCTTAATCTATTTGGCCTTAGC
GCATATGATTAAGTTTCGTGGTCATTTTTTGATTGAGGGAGATTTAAATCCTGAT
AATAGTGATGTGGACAAACTATTTATCCAGTTGGTACAAACCTACAATCAATTATT
TGAAGAAAACCCTATTAACGCAAGTGGAGTAGATGCTAAAGCGATTCTTTCTGCA
CGATTGAGTAAATCAAGACGATTAGAAAATCTCATTGCTCAGCTCCCCGGTGAG
AAGAAAAATGGCTTATTTGGGAATCTCATTGCTTTGTCATTGGGTTTGACCCCTA
ATTTTAAATCAAATTTTGATTTGGCAGAAGATGCTAAATTACAGCTTTCAAAAGAT
ACTTACGATGATGATTTAGATAATTTATTGGCGCAAATTGGAGATCAATATGCTG
ATTTGTTTTTGGCAGCTAAGAATTTATCAGATGCTATTTTACTTTCAGATATCCTA
AGAGTAAATACTGAAATAACTAAGGCTCCCCTATCAGCTTCAATGATTAAACGCT
ACGATGAACATCATCAAGACTTGACTCTTTTAAAAGCTTTAGTTCGACAACAACT
TCCAGAAAAGTATAAAGAAATCTTTTTTGATCAATCAAAAAACGGATATGCAGGT
TATATTGATGGGGGAGCTAGCCAAGAAGAATTTTATAAATTTATCAAACCAATTTT
AGAAAAAATGGATGGTACTGAGGAATTATTGGTGAAACTAAATCGTGAAGATTTG
CTGCGCAAGCAACGGACCTTTGACAACGGCTCTATTCCCCATCAAATTCACTTG
GGTGAGCTGCATGCTATTTTGAGAAGACAAGAAGACTTTTATCCATTTTTAAAAG
ACAATCGTGAGAAGATTGAAAAAATCTTGACTTTTCGAATTCCTTATTATGTTGGT
CCATTGGCGCGTGGCAATAGTCGTTTTGCATGGATGACTCGGAAGTCTGAAGAA
ACAATTACCCCATGGAATTTTGAAGAAGTTGTCGATAAAGGTGCTTCAGCTCAAT
CATTTATTGAACGCATGACAAACTTTGATAAAAATCTTCCAAATGAAAAAGTACTA
CCAAAACATAGTTTGCTTTATGAGTATTTTACGGTTTATAACGAATTGACAAAGGT

CAAATATGTTACTGAAGGAATGCGAAAACCAGCATTTCTTTCAGGTGAACAGAA
GAAAGCCATTGTTGATTTACTCTTCAAAACAAATCGAAAAGTAACCGTTAAGCAA
TTAAAAGAAGATTATTTCAAAAAAATAGAATGTTTTGATAGTGTTGAAATTTCAGG
AGTTGAAGATAGATTTAATGCTTCATTAGGTACCTACCATGATTTGCTAAAAATTA
TTAAAGATAAAGATTTTTTGGATAATGAAGAAAATGAAGATATCTTAGAGGATATT
GTTTTAACATTGACCTTATTTGAAGATAGGGAGATGATTGAGGAAAGACTTAAAA
CATATGCTCACCTCTTTGATGATAAGGTGATGAAACAGCTTAAACGTCGCCGTTA
TACTGGTTGGGGACGTTTGTCTCGAAAATTGATTAATGGTATTAGGGATAAGCAA
TCTGGCAAAACAATATTAGATTTTTTGAAATCAGATGGTTTTGCCAATCGCAATTT
TATGCAGCTGATCCATGATGATAGTTTGACATTTAAAGAAGACATTCAAAAAGCA
CAAGTGTCTGGACAAGGCGATAGTTTACATGAACATATTGCAAATTTAGCTGGTA
GCCCTGCTATTAAAAAAGGTATTTTACAGACTGTAAAAGTTGTTGATGAATTGGT
CAAAGTAATGGGGCGGCATAAGCCAGAAAATATCGTTATTGAAATGGCACGTGA
AAATCAGACAACTCAAAAGGGCCAGAAAAATTCGCGAGAGCGTATGAAACGAAT
CGAAGAAGGTATCAAAGAATTAGGAAGTCAGATTCTTAAAGAGCATCCTGTTGA
AAATACTCAATTGCAAAATGAAAAGCTCTATCTCTATTATCTCCAAAATGGAAGA
GACATGTATGTGGACCAAGAATTAGATATTAATCGTTTAAGTGATTATGATGTCG
ATGCCATTGTTCCACAAAGTTTCCTTAAAGACGATTCAATAGACAATAAGGTCTT
AACGCGTTCTGATAAAAATCGTGGTAAATCGGATAACGTTCCAAGTGAAGAAGT
AGTCAAAAAGATGAAAAACTATTGGAGACAACTTCTAAACGCCAAGTTAATCACT
CAACGTAAGTTTGATAATTTAACGAAAGCTGAACGTGGAGGTTTGAGTGAACTTG
ATAAAGCTGGTTTTATCAAACGCCAATTGGTTGAAACTCGCCAAATCACTAAGCA
TGTGGCACAAATTTTGGATAGTCGCATGAATACTAAATACGATGAAAATGATAAA
CTTATTCGAGAGGTTAAAGTGATTACCTTAAAATCTAAATTAGTTTCTGACTTCCG
AAAAGATTTCCAATTCTATAAAGTACGTGAGATTAACAATTACCATCATGCCCAT
GATGCGTATCTAAATGCCGTCGTTGGAACTGCTTTGATTAAGAAATATCCAAAAC
TTGAATCGGAGTTTGTCTATGGTGATTATAAAGTTTATGATGTTCGTAAAATGATT
GCTAAGTCTGAGCAAGAAATAGGCAAAGCAACCGCAAAATATTTCTTTTACTCTA
ATATCATGAACTTCTTCAAAACAGAAATTACACTTGCAAATGGAGAGATTCGCAA
ACGCCCTCTAATCGAAACTAATGGGGAAACTGGAGAAATTGTCTGGGATAAAGG
GCGAGATTTTGCCACAGTGCGCAAAGTATTGTCCATGCCCCAAGTCAATATTGT
CAAGAAAACAGAAGTACAGACAGGCGGATTCTCCAAGGAGTCAATTTTACCAAA
AAGAAATTCGGACAAGCTTATTGCTCGTAAAAAAGACTGGGATCCAAAAAAATAT
GGTGGTTTTGATAGTCCAACGGTAGCTTATTCAGTCCTAGTGGTTGCTAAGGTG
GAAAAAGGGAAATCGAAGAAGTTAAAATCCGTTAAAGAGTTACTAGGGATCACA
ATTATGGAAAGAAGTTCCTTTGAAAAAAATCCGATTGACTTTTTAGAAGCTAAAG
GATATAAGGAAGTTAAAAAAGACTTAATCATTAAACTACCTAAATATAGTCTTTTT
GAGTTAGAAAACGGTCGTAAACGGATGCTGGCTAGTGCCGGAGAATTACAAAAA
GGAAATGAGCTGGCTCTGCCAAGCAAATATGTGAATTTTTTATATTTAGCTAGTC
ATTATGAAAAGTTGAAGGGTAGTCCAGAAGATAACGAACAAAAACAATTGTTTGT
GGAGCAGCATAAGCATTATTTAGATGAGATTATTGAGCAAATCAGTGAATTTTCT
AAGCGTGTTATTTTAGCAGATGCCAATTTAGATAAAGTTCTTAGTGCATATAACA
AACATAGAGACAAACCAATACGTGAACAAGCAGAAAATATTATTCATTTATTTAC
GTTGACGAATCTTGGAGCTCCCGCTGCTTTTAAATATTTTGATACAACAATTGAT

CGTAAACGATATACGTCTACAAAAGAAGTTTTAGATGCCACTCTTATCCATCAAT
CCATCACTGGTCTTTATGAAACACGCATTGATTTGAGTCAGCTAGGAGGTCCTA
GGGGAGGTGGCTCAGAAGGTGGCGGATCTGAAGGTGGCTCTGGAactagtATGA
GCGATAAAATTATTCACCTGACTGACGACAGTTTTGACACGGATGTACTCAAAGC
GGACGGGGCGATCCTCGTCGATTTCTGGGCAGAGTGGTGCGGTCCGTGCAAAA
TGATCGCCCCGATTCTGGATGAAATCGCTGACGAATATCAGGGCAAACTGACCG
TTGCAAAACTGAACATCGATCAAAACCCTGGCACTGCGCCGAAATATGGCATCC
GTGGTATCCCGACTCTGCTGCTGTTCAAAAACGGTGAAGTGGCGGCAACCAAA
GTGGGTGCACTGTCTAAAGGTCAGTTGAAAGAGTTCCTCGACGCTAACCTGGCC
GGTTCTGGTTCTGGCCATATGCACCATCATCATCATCATTCTTCTGGTCTGGTGC
CACGCGGTTCTGGTATGAAAGAAACCGCTGCTGCTAAATTCGAACGCCAGCACA
TGGACAGCCCAGATCTGGGTACCGACGACGACGACAAGGCCATGGCTGATATC
ggatccCATATGGAAATGATGCTCGATAAGAAACAGATTCGTGCGATCTTTCTCTTT
GAGTTTAAAATGGGTCGCAAAGCGGCGGAGACGACGCGTAATATTAACAACGC
GTTCGGTCCTGGCACCGCGAACGAGCGTACCGTGCAATGGTGGTTCAAAAAGT
TTCGCAAAGGCGACGAATCTCTGGAGGACGAAGAGCGTTCTGGCCGCCCGTCC
GAGGTTGACAACGACCAGCTGCGTGCAATCATCGAAGCTGATCCGCTGACTAC
CACCCGCGAAGTTGCTGAAGAACTGAATGTGGATCACTCTACTGTGGTTCGCCA
CCTGAAACAGATCGGTAAAGTAAAAAAACTGGACAAATGGGTTCCTCATGAACT
GTCTGAAAACCAGAAAAACCGTCGTTTCGAAGTTAGCTCCTCTCTGATTCTGCGT
AACAACAACGAACCGTTCCTGGATCGTATCGTAACCTGTGATGAGAAATGGATT
CTGTATGATAACCGTCGCCGCTCTGCTCAGTGGCTGGATCGCGAAGAAGCTCC
AAAACACTTCCCGAAACCGAATCTGCACCAGAAGAAAGTCATGGTAACCGTATG
GTGGTCTGCCGCAGGTGTTATCCACTATTCCTTCCTGAACCCGGGCGAAACTAT
CACCAGCGAAAAATACTGCCAGCAGATTGACGAAATGCACCGTAAACTGCAGCG
TCTGCAGCCAGCACTGGTGAATCGTAAAGGTCCGATCCTGCTGCATGATAACGC
CCGTCCGCACGTTGCCCAACCGACCCTGCAGAAACTGAACGAACTGGGCTATG
AAGTTCTGCCACACCCGCCGTACTCCCCGGATCTGTCCCCGACTGACTACCATT
TCTTCAAGCATCTGGACAACTTCCTGCAGGGTAAACGTTTTCACAACCAACAGG
ACGCAGAAAACGCTTTCCAGGAGTTCGTCGAAAGCCGTTCCACTGACTTCTACG
CGACCGGTATCAACAAGCTGATCAGCCGTTGGCAGAAATGCGTGGACTGTAAC
GGCAGCTACTTCGATTAAGACGTCTTAATAACTAAAAATATGGTATAATACTCTTA
ATAAATGCAGTAATACAGGGGCTTTTCAAGACTGAAGTCTAGCTGAGACAAATA
GTGCGATTACGAAATTTTTTAGACAAAAATAGTCTACGAGGTTTTAGAGCTATGC
TGTTTTGAATGGTCCCAAAACGGTTTTCCCAGTCACGACGTTGTAAAACGAGTTT
TAGAGCTATGCTGTTTTGAATGGTCCCAAAACTTCAGCACACTGAGACTTGTTGA
GTTCCATGTTTTAGAGCTATGCTGTTTTGAATGGACTCCATTCAACATTGCCGAT
GATAACTTGAGAAAGAGGGTTAATACCAGCAGTCGGATACCTTCCTATTCTTTCT
GTTAAAGCGTTTTCATGTTATAATAGGCAAAAGAAGAGTAGTGTGATCGTCCATT
CCGACAGCATCGCCAGTCACTATGGCGTGCTGCTAGCGCTATATGCGTTGATGC
AATTTCTATGCGCACCCGTTCTCGGAGCACTGTCCGACCGCTTTGGCCGCCGC
CCAGTCCTGCTCGCTTCGCTACTTGGAGCCACTATCGACTACGCGATCATGGCG
ACCACACCCGTCCTGTGGATCCTCTACGCCGGACGCATCGTGGCCGGCATCAC
CGGCGCCACAGGTGCGGTTGCTGGCGCCTATATCGCCGACATCACCGATGGGG

AAGATCGGGCTCGCCACTTCGGGCTCATGAGCGCTTGTTTCGGCGTGGGTATG
GTGGCAGGCCCCGTGGCCGGGGGACTGTTGGGCGCCATCTCCTTGCATGCAC
CATTCCTTGCGGCGGCGGTGCTCAACGGCCTCAACCTACTACTGGGCTGCTTC
CTAATGCAGGAGTCGCATAAGGGAGAGCGTCGACCGATGCCCTTGAGAGCCTT
CAACCCAGTCAGCTCCTTCCGGTGGGCGCGGGGCATGACTATCGTCGCCGCAC
TTATGACTGTCTTCTTTATCATGCAACTCGTAGGACAGGTGCCGGCAGCGCTCT
GGGTCATTTTCGGCGAGGACCGCTTTCGCTGGAGCGCGACGATGATCGGCCTG
TCGCTTGCGGTATTCGGAATCTTGCACGCCCTCGCTCAAGCCTTCGTCACTGGT
CCCGCCACCAAACGTTTCGGCGAGAAGCAGGCCATTATCGCCGGCATGGCGGC
CGACGCGCTGGGCTACGTCTTGCTGGCGTTCGCGACGCGAGGCTGGATGGCC
TTCCCCATTATGATTCTTCTCGCTTCCGGCGGCATCGGGATGCCCGCGTTGCAG
GCCATGCTGTCCAGGCAGGTAGATGACGACCATCAGGGACAGCTTCAAGGATC
GCTCGCGGCTCTTACCAGCCTAACTTCGATCATTGGACCGCTGATCGTCACGGC
GATTTATGCCGCCTCGGCGAGCACATGGAACGGGTTGGCATGGATTGTAGGCG
CCGCCCTATACCTTGTCTGCCTCCCCGCGTTGCGTCGCGGTGCATGGAGCCGG
GCCACCTCGACCTGAATGGAAGCCGGCGGCACCTCGCTAACGGATTCACCACT
CCAAGAATTGGAGCCAATCAATTCTTGCGGAGAACTGTGAATGCGCAAACCAAC
CCTTGGCAGAACATATCCATCGCGTCCGCCATCTCCAGCAGCCGCACGCGGCG
CATCTCGGGCAGCGTTGGGTCCTGGCCACGGGTGCGCATGATCGTGCTCCTGT
CGTTGAGGACCCGGCTAGGCTGGCGGGGTTGCCTTACTGGTTAGCAGAATGAA
TCACCGATACGCGAGCGAACGTGAAGCGACTGCTGCTGCAAAACGTCTGCGAC
CTGAGCAACAACATGAATGGTCTTCGGTTTCCGTGTTTCGTAAAGTCTGGAAAC
GCGGAAGTCCCCTACGTGCTGCTGAAGTTGCCCGCAACAGAGAGTGGAACCAA
CCGGTGATACCACGATACTATGACTGAGAGTCAACGCCATGAGCGGCCTCATTT
CTTATTCTGAGTTACAACAGTCCGCACCGCTGTCCGGTAGCTCCTTCCGGTGGG
CGCGGGGCATGACTATCGTCGCCGCACTTATGACTGTCTTCTTTATCATGCAAC
TCGTAGGACAGGTGCCGGCAGCGCCCAACAGTCCCCCGGCCACGGGGCCTGC
CACCATACCCACGCCGAAACAAGCGCCCTGCACCATTATGTTCCGGATCTGCAT
CGCAGGATGCTGCTGGCTACCCTGTGGAACACCTACATCTGTATTAACGAAGCG
CTAACCGTTTTTATCAGGCTCTGGGAGGCAGAATAAATGATCATATCGTCAATTA
TTACCTCCACGGGGAGAGCCTGAGCAAACTGGCCTCAGGCATTTGAGAAGCAC
ACGGTCACACTGCTTCCGGTAGTCAATAAACCGGTAAACCAGCAATAGACATAA
GCGGCTATTTAACGACCCTGCCCTGAACCGACGACCGGGTCGAATTTGCTTTCG
AATTTCTGCCATTCATCCGCTTATTATCACTTATTCAGGCGTAGCACCAGGCGTT
TAAGGGCACCAATAACTGCCTTAAAAAAATTACGCCCCGCCCTGCCACTCATCG
CAGTACTGTTGTAATTCATTAAGCATTCTGCCGACATGGAAGCCATCACAGACG
GCATGATGAACCTGAATCGCCAGCGGCATCAGCACCTTGTCGCCTTGCGTATAA
TATTTGCCCATGGTGAAAACGGGGGCGAAGAAGTTGTCCATATTGGCCACGTTT
AAATCAAAACTGGTGAAACTCACCCAGGGATTGGCTGAGACGAAAAACATATTC
TCAATAAACCCTTTAGGGAAATAGGCCAGGTTTTCACCGTAACACGCCACATCTT
GCGAATATATGTGTAGAAACTGCCGGAAATCGTCGTGGTATTCACTCCAGAGCG
ATGAAAACGTTTCAGTTTGCTCATGGAAAACGGTGTAACAAGGGTGAACACTAT
CCCATATCACCAGCTCACCGTCTTTCATTGCCATACG

pRC2311

TTTCTCTGTCCTTCCTGTGCGACGGTTACGCCGCTCCATGAGCTTATCGCGAAT
AAATACCTGTGACGGAAGATCACTTCGCAGAATAAATAAATCCTGGTGTCCCTGT
TGATACCGGGAAGCCCTGGGCCAACTTTTGGCGAAAATGAGACGTTGATCGGC
ACGTAAGAGGTTCCAACTTTCACCATAATGAAATAAGATCACTACCGGGCGTATT
TTTTGAGTTATCGAGATTTTCAGGAGCTAAGGAAGCTAAAATGGAGAAAAAAATC
ACTGGATATACCACCGTTGATATATCCCAATGGCATCGTAAAGAACATTTTGAGG
CATTTCAGTCAGTTGCTCAATGTACCTATAACCAGACCGTTCAGCTGGATATTAC
GGCCTTTTTAAAGACCGTAAAGAAAAATAAGCACAAGTTTTATCCGGCCTTTATT
CACATTCTTGCCCGCCTGATGAATGCTCATCCGGAGTTCCGTATGGCAATGAAA
GACGGTGAGCTGGTGATATGGGATAGTGTTCACCCTTGTTACACCGTTTTCCAT
GAGCAAACTGAAACGTTTTCATCGCTCTGGAGTGAATACCACGACGATTTCCGG
CAGTTTCTACACATATATTCGCAAGATGTGGCGTGTTACGGTGAAAACCTGGCC
TATTTCCCTAAAGGGTTTATTGAGAATATGTTTTTCGTCTCAGCCAATCCCTGGG
TGAGTTTCACCAGTTTTGATTTAAACGTGGCCAATATGGACAACTTCTTCGCCCC
CGTTTTCACCATGGGCAAATATTATACGCAAGGCGACAAGGTGCTGATGCCGCT
GGCGATTCAGGTTCATCATGCCGTTTGTGATGGCTTCCATGTCGGCAGAATGCT
TAATGAATTACAACAGTACTGCGATGAGTGGCAGGGCGGGGCGTAATTTTTTTA
AGGCAGTTATTGGTGCCCTTAAACGCCTGGTTGCTACGCCTGAATAAGTGATAA
TAAGCGGATGAATGGCAGAAATTCGATGATAAGCTGTCAAACATGAGAATTGGT
CGACGGCGCGCCAAAGCTTGCATGCCTGCAGCCGCGTAACCTGGCAAAATCGG
TTACGGTTGAGTAATAAATGGATGCCCTGCGTAAGCGGGGCACATTTCATTACC
TCTTTCTCCGCACCCGACATAGATAATAACTTCGTATAGTATACATTATACGAAG
TTATCTAGTAGACTTAATTAAGGATCGATCCGGCGCGCCAATAGTCATGCCCCG
CGCCCACCGGAAGGAGCTGACTGGGTTGAAGGCTCTCAAGGGCATCGGTCGA
GCTTGACATTGTAGGACTATATTGCTCTAATAAATTTGCGGCCGCTAATACGACT
CACTATAGGGAGAGGATCCGCGGGATTACGAAATCATCCTGTGGAGCTTAGTAG
GTTTAGCAAGATGGCAGCGCCTAAATGTAGAATGATAAAAGGATTAAGAGATTA
ATTTCCCTAAAAATGATAAAACAAGCGTTTTGAAAGCGCTTGTTTTTTTGGTTTGC
AGTCAGAGTAGAATAGAAGTATCAAAAAAAGCACCGACTCGGTGCCACTTTTTC
AAGTTGATAACGGACTAGCCTTATTTTAACTTGCTATGCTGTTTTGAATGGTTCC
AACAAGATTATTTTATAACTTTTATAACAAATAATCAAGGAGAAATTCAAAGAAAT
TTATCAGCCATAAAACAATACTTAATACTATAGAATGATAACAAAATAAACTACTT
TTTAAAAGAATTTTGTGTTATAATCTATTTATTATTAAGTATTGGGTAATATTTTTT
GAAGAGCGGCCGCGTTAATAACAGGGGACGTGGTAATCCGTCCCCTTTTTATTT
CTGACTGAGTTAATAACAGGCCTGCTTCGGCAGGCCTTTTTATTTCTGACTGAGT
TCTTCTCAGGCCTGCTGGTAATCGCAGGCCTTTTTATTTTCTAGTCTGACTGACT
GACTGACTGACTGACTGACTGACTGACTGACTGACTGACTGACTGACTGACTGA
CTGACTGACTGACTGACTGACTGACTGACTGACTGACTGACTGACTGACTGACT
GACTGACTGACTGACTGACTGACTGACTGACTGACTGACTGACTGACTGACTGA
CTGACTGACCTCGAGATGGATAAGAAATACTCAATAGGCTTAGCTATCGGCACA
AATAGCGTCGGATGGGCGGTGATCACTGATGAATATAAGGTTCCGTCTAAAAAG
TTCAAGGTTCTGGGAAATACAGACCGCCACAGTATCAAAAAAAATCTTATAGGG
GCTCTTTTATTTGACAGTGGAGAGACAGCGGAAGCGACTCGTCTCAAACGGACA

GCTCGTAGAAGGTATACACGTCGGAAGAATCGTATTTGTTATCTACAGGAGATTT
TTTCAAATGAGATGGCGAAAGTAGATGATAGTTTCTTTCATCGACTTGAAGAGTC
TTTTTTGGTGGAAGAAGACAAGAAGCATGAACGTCATCCTATTTTTGGAAATATA
GTAGATGAAGTTGCTTATCATGAGAAATATCCAACTATCTATCATCTGCGAAAAA
AATTGGTAGATTCTACTGATAAAGCGGATTTGCGCTTAATCTATTTGGCCTTAGC
GCATATGATTAAGTTTCGTGGTCATTTTTTGATTGAGGGAGATTTAAATCCTGAT
AATAGTGATGTGGACAAACTATTTATCCAGTTGGTACAAACCTACAATCAATTATT
TGAAGAAAACCCTATTAACGCAAGTGGAGTAGATGCTAAAGCGATTCTTTCTGCA
CGATTGAGTAAATCAAGACGATTAGAAAATCTCATTGCTCAGCTCCCCGGTGAG
AAGAAAAATGGCTTATTTGGGAATCTCATTGCTTTGTCATTGGGTTTGACCCCTA
ATTTTAAATCAAATTTTGATTTGGCAGAAGATGCTAAATTACAGCTTTCAAAAGAT
ACTTACGATGATGATTTAGATAATTTATTGGCGCAAATTGGAGATCAATATGCTG
ATTTGTTTTTGGCAGCTAAGAATTTATCAGATGCTATTTTACTTTCAGATATCCTA
AGAGTAAATACTGAAATAACTAAGGCTCCCCTATCAGCTTCAATGATTAAACGCT
ACGATGAACATCATCAAGACTTGACTCTTTTAAAAGCTTTAGTTCGACAACAACT
TCCAGAAAAGTATAAAGAAATCTTTTTTGATCAATCAAAAAACGGATATGCAGGT
TATATTGATGGGGGAGCTAGCCAAGAAGAATTTTATAAATTTATCAAACCAATTTT
AGAAAAAATGGATGGTACTGAGGAATTATTGGTGAAACTAAATCGTGAAGATTTG
CTGCGCAAGCAACGGACCTTTGACAACGGCTCTATTCCCCATCAAATTCACTTG
GGTGAGCTGCATGCTATTTTGAGAAGACAAGAAGACTTTTATCCATTTTTAAAAG
ACAATCGTGAGAAGATTGAAAAAATCTTGACTTTTCGAATTCCTTATTATGTTGGT
CCATTGGCGCGTGGCAATAGTCGTTTTGCATGGATGACTCGGAAGTCTGAAGAA
ACAATTACCCCATGGAATTTTGAAGAAGTTGTCGATAAAGGTGCTTCAGCTCAAT
CATTTATTGAACGCATGACAAACTTTGATAAAAATCTTCCAAATGAAAAAGTACTA
CCAAAACATAGTTTGCTTTATGAGTATTTTACGGTTTATAACGAATTGACAAAGGT
CAAATATGTTACTGAAGGAATGCGAAAACCAGCATTTCTTTCAGGTGAACAGAA
GAAAGCCATTGTTGATTTACTCTTCAAAACAAATCGAAAAGTAACCGTTAAGCAA
TTAAAAGAAGATTATTTCAAAAAAATAGAATGTTTTGATAGTGTTGAAATTTCAGG
AGTTGAAGATAGATTTAATGCTTCATTAGGTACCTACCATGATTTGCTAAAAATTA
TTAAAGATAAAGATTTTTTGGATAATGAAGAAAATGAAGATATCTTAGAGGATATT
GTTTTAACATTGACCTTATTTGAAGATAGGGAGATGATTGAGGAAAGACTTAAAA
CATATGCTCACCTCTTTGATGATAAGGTGATGAAACAGCTTAAACGTCGCCGTTA
TACTGGTTGGGGACGTTTGTCTCGAAAATTGATTAATGGTATTAGGGATAAGCAA
TCTGGCAAAACAATATTAGATTTTTTGAAATCAGATGGTTTTGCCAATCGCAATTT
TATGCAGCTGATCCATGATGATAGTTTGACATTTAAAGAAGACATTCAAAAAGCA
CAAGTGTCTGGACAAGGCGATAGTTTACATGAACATATTGCAAATTTAGCTGGTA
GCCCTGCTATTAAAAAAGGTATTTTACAGACTGTAAAAGTTGTTGATGAATTGGT
CAAAGTAATGGGGCGGCATAAGCCAGAAAATATCGTTATTGAAATGGCACGTGA
AAATCAGACAACTCAAAAGGGCCAGAAAAATTCGCGAGAGCGTATGAAACGAAT
CGAAGAAGGTATCAAAGAATTAGGAAGTCAGATTCTTAAAGAGCATCCTGTTGA
AAATACTCAATTGCAAAATGAAAAGCTCTATCTCTATTATCTCCAAAATGGAAGA
GACATGTATGTGGACCAAGAATTAGATATTAATCGTTTAAGTGATTATGATGTCG
ATGCCATTGTTCCACAAAGTTTCCTTAAAGACGATTCAATAGACAATAAGGTCTT
AACGCGTTCTGATAAAAATCGTGGTAAATCGGATAACGTTCCAAGTGAAGAAGT

AGTCAAAAAGATGAAAAACTATTGGAGACAACTTCTAAACGCCAAGTTAATCACT
CAACGTAAGTTTGATAATTTAACGAAAGCTGAACGTGGAGGTTTGAGTGAACTTG
ATAAAGCTGGTTTTATCAAACGCCAATTGGTTGAAACTCGCCAAATCACTAAGCA
TGTGGCACAAATTTTGGATAGTCGCATGAATACTAAATACGATGAAAATGATAAA
CTTATTCGAGAGGTTAAAGTGATTACCTTAAAATCTAAATTAGTTTCTGACTTCCG
AAAAGATTTCCAATTCTATAAAGTACGTGAGATTAACAATTACCATCATGCCCAT
GATGCGTATCTAAATGCCGTCGTTGGAACTGCTTTGATTAAGAAATATCCAAAAC
TTGAATCGGAGTTTGTCTATGGTGATTATAAAGTTTATGATGTTCGTAAAATGATT
GCTAAGTCTGAGCAAGAAATAGGCAAAGCAACCGCAAAATATTTCTTTTACTCTA
ATATCATGAACTTCTTCAAAACAGAAATTACACTTGCAAATGGAGAGATTCGCAA
ACGCCCTCTAATCGAAACTAATGGGGAAACTGGAGAAATTGTCTGGGATAAAGG
GCGAGATTTTGCCACAGTGCGCAAAGTATTGTCCATGCCCCAAGTCAATATTGT
CAAGAAAACAGAAGTACAGACAGGCGGATTCTCCAAGGAGTCAATTTTACCAAA
AAGAAATTCGGACAAGCTTATTGCTCGTAAAAAAGACTGGGATCCAAAAAAATAT
GGTGGTTTTGATAGTCCAACGGTAGCTTATTCAGTCCTAGTGGTTGCTAAGGTG
GAAAAAGGGAAATCGAAGAAGTTAAAATCCGTTAAAGAGTTACTAGGGATCACA
ATTATGGAAAGAAGTTCCTTTGAAAAAAATCCGATTGACTTTTTAGAAGCTAAAG
GATATAAGGAAGTTAAAAAAGACTTAATCATTAAACTACCTAAATATAGTCTTTTT
GAGTTAGAAAACGGTCGTAAACGGATGCTGGCTAGTGCCGGAGAATTACAAAAA
GGAAATGAGCTGGCTCTGCCAAGCAAATATGTGAATTTTTTATATTTAGCTAGTC
ATTATGAAAAGTTGAAGGGTAGTCCAGAAGATAACGAACAAAAACAATTGTTTGT
GGAGCAGCATAAGCATTATTTAGATGAGATTATTGAGCAAATCAGTGAATTTTCT
AAGCGTGTTATTTTAGCAGATGCCAATTTAGATAAAGTTCTTAGTGCATATAACA
AACATAGAGACAAACCAATACGTGAACAAGCAGAAAATATTATTCATTTATTTAC
GTTGACGAATCTTGGAGCTCCCGCTGCTTTTAAATATTTTGATACAACAATTGAT
CGTAAACGATATACGTCTACAAAAGAAGTTTTAGATGCCACTCTTATCCATCAAT
CCATCACTGGTCTTTATGAAACACGCATTGATTTGAGTCAGCTAGGAGGTCCTA
GGGGAGGTGGCTCAGAAGGTGGCGGATCTGAAGGTGGCTCTGGAACTAGTATG
AGCGATAAAATTATTCACCTGACTGACGACAGTTTTGACACGGATGTACTCAAAG
CGGACGGGGCGATCCTCGTCGATTTCTGGGCAGAGTGGTGCGGTCCGTGCAAA
ATGATCGCCCCGATTCTGGATGAAATCGCTGACGAATATCAGGGCAAACTGACC
GTTGCAAAACTGAACATCGATCAAAACCCTGGCACTGCGCCGAAATATGGCATC
CGTGGTATCCCGACTCTGCTGCTGTTCAAAAACGGTGAAGTGGCGGCAACCAAA
GTGGGTGCACTGTCTAAAGGTCAGTTGAAAGAGTTCCTCGACGCTAACCTGGCC
GGTTCTGGTTCTGGCCATATGCACCATCATCATCATCATTCTTCTGGTCTGGTGC
CACGCGGTTCTGGTATGAAAGAAACCGCTGCTGCTAAATTCGAACGCCAGCACA
TGGACAGCCCAGATCTGGGTACCGACGACGACGACAAGGCCATGGCTGATATC
GGATCCCATATGGAAATGATGCTCGATAAGAAACAGATTCGTGCGATCTTTCTCT
TTGAGTTTAAAATGGGTCGCAAAGCGGCGGAGACGACGCGTAATATTAACAACG
CGTTCGGTCCTGGCACCGCGAACGAGCGTACCGTGCAATGGTGGTTCAAAAAG
TTTCGCAAAGGCGACGAATCTCTGGAGGACGAAGAGCGTTCTGGCCGCCCGTC
CGAGGTTGACAACGACCAGCTGCGTGCAATCATCGAAGCTGATCCGCTGACTA
CCACCCGCGAAGTTGCTGAAGAACTGAATGTGGATCACTCTACTGTGGTTCGCC
ACCTGAAACAGATCGGTAAAGTAAAAAAACTGGACAAATGGGTTCCTCATGAAC

TGTCTGAAAACCAGAAAAACCGTCGTTTCGAAGTTAGCTCCTCTCTGATTCTGCG
TAACAACAACGAACCGTTCCTGGATCGTATCGTAACCTGTGATGAGAAATGGAT
TCTGTATGATAACCGTCGCCGCTCTGCTCAGTGGCTGGATCGCGAAGAAGCTCC
AAAACACTTCCCGAAACCGAATCTGCACCAGAAGAAAGTCATGGTAACCGTATG
GTGGTCTGCCGCAGGTGTTATCCACTATTCCTTCCTGAACCCGGGCGAAACTAT
CACCAGCGAAAAATACTGCCAGCAGATTGACGAAATGCACCGTAAACTGCAGCG
TCTGCAGCCAGCACTGGTGAATCGTAAAGGTCCGATCCTGCTGCATGATAACGC
CCGTCCGCACGTTGCCCAACCGACCCTGCAGAAACTGAACGAACTGGGCTATG
AAGTTCTGCCACACCCGCCGTACTCCCCGGATCTGTCCCCGACTGACTACCATT
TCTTCAAGCATCTGGACAACTTCCTGCAGGGTAAACGTTTTCACAACCAACAGG
ACGCAGAAAACGCTTTCCAGGAGTTCGTCGAAAGCCGTTCCACTGACTTCTACG
CGACCGGTATCAACAAGCTGATCAGCCGTTGGCAGAAATGCGTGGACTGTAAC
GGCAGCTACTTCGATTAAGACGTCTTAATAACTAAAAATATGGTATAATACTCTTA
ATAAATGCAGTAATACAGGGGCTTTTCAAGACTGAAGTCTAGCTGAGACAAATA
GTGCGATTACGAAATTTTTTAGACAAAAATAGTCTACGAGGTTTTAGAGCTATGC
TGTTTTGAATGGTCCCAAAACGGTTTTCCCAGTCACGACGTTGTAAAACGAGTTT
TAGAGCTATGCTGTTTTGAATGGTCCCAAAACTTCAGCACACTGAGACTTGTTGA
GTTCCATGTTTTAGAGCTATGCTGTTTTGAATGGACTCCATTCAACATTGCCGAT
GATAACTTGAGAAAGAGGGTTAATACCAGCAGTCGGATACCTTCCTATTCTTTCT
GTTAAAGCGTTTTCATGTTATAATAGGCAAAAGAAGAGTAGTGTGATCGCCGCG
GATCCTTCTATAGTGTCACCTAAATGTCGACGGCCAGGCGGCCGCCAGGCCTA
CCCACTAGTCAATTCGGGAGGATCGAAACGGCAGATCGCAAAAAACAGTACATA
CAGAAGGAGACATGAACATGAACATCAAAAAAATTGTAAAACAAGCCACAGTTCT
GACTTTTACGACTGCACTTCTGGCAGGAGGAGCGACTCAAGCCTTCGCGAAAGA
AAATAACCAAAAAGCATACAAAGAAACGTACGGCGTCTCTCATATTACACGCCAT
GATATGCTGCAGATCCCTAAACAGCAGCAAAACGAAAAATACCAAGTGCCTCAA
TTCGATCAATCAACGATTAAAAATATTGAGTCTGCAAAAGGACTTGATGTGTGGG
ACAGCTGGCCGCTGCAAAACGCTGACGGAACAGTAGCTGAATACAACGGCTAT
CACGTTGTGTTTGCTCTTGCGGGAAGCCCGAAAGACGCTGATGACACATCAATC
TACATGTTTTATCAAAAGGTCGGCGACAACTCAATCGACAGCTGGAAAAACGCG
GGCCGTGTCTTTAAAGACAGCGATAAGTTCGACGCCAACGATCCGATCCTGAAA
GATCAGACGCAAGAATGGTCCGGTTCTGCAACCTTTACATCTGACGGAAAAATC
CGTTTATTCTACACTGACTATTCCGGTAAACATTACGGCAAACAAAGCCTGACAA
CAGCGCAGGTAAATGTGTCAAAATCTGATGACACACTCAAAATCAACGGAGTGG
AAGATCACAAAACGATTTTTGACGGAGACGGAAAAACATATCAGAACGTTCAGC
AGTTTATCGATGAAGGCAATTATACATCCGGCGACAACCATACGCTGAGAGACC
CTCACTACGTTGAAGACAAAGGCCATAAATACCTTGTATTCGAAGCCAACACGG
GAACAGAAAACGGATACCAAGGCGAAGAATCTTTATTTAACAAAGCGTACTACG
GCGGCGGCACGAACTTCTTCCGTAAAGAAAGCCAGAAGCTTCAGCAGAGCGCT
AAAAAACGCGATGCTGAGTTAGCGAACGGCGCCCTCGGTATCATAGAGTTAAAT
AATGATTACACATTGAAAAAAGTAATGAAGCCGCTGATCACTTCAAACACGGTAA
CTGATGAAATCGAGCGCGCGAATGTTTTCAAAATGAACGGCAAATGGTACTTGT
TCACTGATTCACGCGGTTCAAAAATGACGATCGATGGTATTAACTCAAACGATAT
TTACATGCTTGGTTATGTATCAAACTCTTTAACCGGCCCTTACAAGCCGCTGAAC

AAAACAGGGCTTGTGCTGCAAATGGGTCTTGATCCAAACGATGTGACATTCACT
TACTCTCACTTCGCAGTGCCGCAAGCCAAAGGCAACAATGTGGTTATCACAAGC
TACATGACAAACAGAGGCTTCTTCGAGGATAAAAAGGCAACATTTGCGCCAAGC
TTCTTAATGAACATCAAAGGCAATAAAACATCCGTTGTCAAAAACAGCATCCTGG
AGCAAGGACAGCTGACAGTCAACTAATAACAGCAAAAAGAAAATGCCGATACTT
CATTGGCATTTTCTTTTATTTCTCAACAAGATGGTGAATTGACTAGTGGGTAGAT
CCACAGGACGGGTGTGGTCGCCATGATCGCGTAGTCGATAGTGGCTCCAAGTA
GCGAAGCGAGCAGGACTGGGCGGCGGCCAAAGCGGTCGGACAGTGCTCCGAG
AACGGGTGCGCATAGAAATTGCATCAACGCATATAGCGCTAGCAGCACGCCATA
GTGACTGGCGATGCTGTCGGAATGGACGATATCCCGCAAGAGGCCCGGCAGTA
CCGGCATAACCAAGCCTATGCCTACAGCATCCAGGGTGACGGTGCCGAGGATG
ACGATGAGCGCATTGTTAGATTTCATACACGGTGCCTGACTGCGTTAGCAATTTA
ACTGTGATAAACTACCGCATTAAAGCTTATCGATGATAAGCTGTCAAACATGAGA
ATTGATCCGGAACCCTTAATATAACTTCGTATAATGTATGCTATACGAAGTTATTA
GGTCCCTCGACTATAGGGTCACCGTCGACAGCGACACACTTGCATCGGATGCA
GCCCGGTTAACGTGCCGGCACGGCCTGGGTAACCAGGTATTTTGTCCACATAA
CCGTGCGCAAAATGTTGTGGATAAGCAGGACACAGCAGCAATCCACAGCAGGC
ATACAACCGCACACCGAGGTTACTCCGTTCTACAGGTTACGACGACATGTCAAT
ACTTGCCCTTGACAGGCATTGATGGAATCGTAGTCTCACGCTGATAGTCTGATC
GACAATACAAGTGGGACCGTGGTCCCAGACCGATAATCAGACCGACAACACGA
GTGGGATCGTGGTCCCAGACTAATAATCAGACCGACGATACGAGTGGGACCGT
GGTCCCAGACTAATAATCAGACCGACGATACGAGTGGGACCGTGGTTCCAGAC
TAATAATCAGACCGACGATACGAGTGGGACCGTGGTCCCAGACTAATAATCAGA
CCGACGATACGAGTGGGACCATGGTCCCAGACTAATAATCAGACCGACGATAC
GAGTGGGACCGTGGTCCCAGTCTGATTATCAGACCGACGATACGAGTGGGACC
GTGGTCCCAGACTAATAATCAGACCGACGATACGAGTGGGACCGTGGTCCCAG
ACTAATAATCAGACCGACGATACGAGTGGGACCGTGGTCCCAGTCTGATTATCA
GACCGACGATACAAGTGGAACAGTGGGCCCAGAGAGAATATTCAGGCCAGTTA
TGCTTTCTGGCCTGTAACAAAGGACATTAAGTAAAGACAGATAAACGTAGACTAA
AACGTGGTCGCATCAGGGTGCTGGCTTTTCAAGTTCCTTAAGAATGGCCTCAAT
TTTCTCTATACACTCAGTTGGAACACGAGACCTGTCCAGGTTAAGCACCATTTTA
TCGCCCTTATACAATACTGTCGCTCCAGGAGCAAACTGATGTCGTGAGCTTAAA
CTAGTTCTTGATGCAGATGACGTTTTAAGCACAGAAGTTAAAAGAGTGATAACTT
CTTCAGCTTCAAATATCACCCCAGCTTTTTTCTGCTCATGAAGGTTAGATGCCTG
CTGCTTAAGTAATTCCTCTTTATCTGTAAAGGCTTTTTGAAGTGCATCACCTGAC
CGGGCAGATAGTTCACCGGGGTGAGAAAAAAGAGCAACAACTGATTTAGGCAAT
TTGGCGGTGTTGATACAGCGGGTAATAATCTTACGTGAAATATTTTCCGCATCAG
CCAGCGCAGAAATATTTCCAGCAAATTCATTCTGCAATCGGCTTGCATAACGCT
GACCACGTTCATAAGCACTTGTTGGGCGATAATCGTTACCCAATCTGGATAATG
CAGCCATCTGCTCATCATCCAGCTCGCCAACCAGAACACGATAATCACTTTCGG
TAAGTGCAGCAGCTTTACGACGGCGACTCCCATCGGCAATTTCTATGACACCAG
ATACTCTTCGACCGAACGCCGGTGTCTGTTGACCAGTCAGTAGAAAAGAAGGGA
TGAGATCATCCAGTGCGTCCTCAGTAAGCAGCTCCTGGTCACGTTCATTACCTG
ACCATACCCGAGAGGTCTTCTCAACACTATCACCCCGGAGCACTTCAAGAGTAA

ACTTCACATCCCGACCACATACAGGCAAAGTAATGGCATTACCGCGAGCCATTA
CTCCTACGCGCGCAATTAACGAATCCACCATCGGGGCAGCTGGTGTCGATAAC
GAAGTATCTTCAACCGGTTGAGTATTGAGCGTATGTTTTGGAATAACAGGCGCA
CGCTTCATTATCTAATCTCCCAGCGTGGTTTAATCAGACGATCGAAAATTTCATT
GCAGACAGGTTCCCAAATAGAAAGAGCATTTCTCCAGGCACCAGTTGAAGAGCG
TTGATCAATGGCCTGTTCAAAAACAGTTCTCATCCGGATCTGACCTTTACCAACT
TCATCCGTTTCACGTACAACATTTTTTAGAACCATGCTTCCCCAGGCATCCCGAA
TTTGCTCCTCCATCCACGGGGACTGAGAGCCATTACTATTGCTGTATTTGGTAA
GCAAAATACGTACATCAGGCTCGAACCCTTTAAGATCAACGTTCTTGAGCAGAT
CACGAAGCATATCGAAAAACTGCAGTGCGGAGGTGTAGTCAAACAACTCAGCAG
GCGTGGGAACAATCAGCACATCAGCAGCACATACGACATTAATCGTGCCGATAC
CCAGGTTAGGCGCGCTGTCAATAACTATGACATCATAGTCATGAGCAACAGTTT
CAATGGCCAGTCGGAGCATCAGGTGTGGATCGGTGGGCAGTTTACCTTCATCAA
ATTTGCCCATTAACTCAGTTTCAATACGGTGCAGAGCCAGACAGGAAGGAATAA
TGTCAAGCCCCGGCCAGCAAGTGGGCTTTATTGCATAAGTGACATCGTCCTTTT
CCCCAAGATAGAAAGGCAGGAGAGTGTCTTCTGCATGAATATGAAGATCTGGTA
CCCATCCGTGATACATTGAGGCTGTTCCCTGGGGGTCGTTACCTTCCACGAGCA
AAACACGTAGCCCCTTCAGAGCCAGATCCTGAGCAAGATGAACAGAAACTGAG
GTTTTGTAAACGCCACCTTTATGGGCAGCAACCCCGATCACCGGTGGAAATACG
TCTTCAGCACGTCGCAATCGCGTACCAAACACATCACGCATATGATTAATTTGTT
CAATTGTATAACCAACACGTTGCTCAACCCGTCCTCGAATTTCCATATCCGGGTG
CGGTAGTCGCCCTGCTTTCTCGGCATCTCTGATAGCCTGAGAAGAAACCCCAAC
TAAATCCGCTGCTTCACCTATTCTCCAGCGCCGGGTTATTTTCCTCGCTTCCGG
GCTGTCATCATTAAACTGTGCAATGGCGATAGCCTTCGTCATTTCATGACCAGC
GTTTATGCACTGGTTAAGTGTTTCCATGAGTTTCATTCTGAACATCCTTTAATCAT
TGCTTTGCGTTTTTTTATTAAATCTTGCAATTTACTGCAAAGCAACAACAAAATCG
CAAAGTCATCAAAAAACCGCAAAGTTGTTTAAAATAAGAGCAACACTACAAAAGG
AGATAAGAAGAGCACATACCTCAGTCACTTATTATCACTAGCGCTCGCCGCAGC
CGTGTAACCGAGCATAGCGAGCGAACTGGCGAGGAAGCAAAGAAGAACTGTTC
TGTCAGATAGCTCTTACGCTCAGCGCAAGAAGAAATATCCACCGTGGGAAAAAC
TCCAGGTAGAGGTACACACGCGGATAGCCAATTCAGAGTAATAAACTGTGATAA
TCAACCCTCATCAATGATGACGAACTAACCCCCGATATCAGGTCACATGACGAA
GGGAAAGAGAAGGAAATCAACTGTGACAAACTGCCCTCAAATTTGGCTTCCTTA
AAAATTACAGTTCAAAAAGTATGAGAAAATCCATGCAGGCTGAAGGAAACAGCA
AAACTGTGACAAATTACCCTCAGTAGGTCAGAACAAATGTGACGAACCACCCTC
AAATCTGTGACAGATAACCCTCAGACTATCCTGTCGTCATGGAAGTGATATCGC
GGAAGGAAAATACGATATGAGTCGTCTGGCGGCCTTTCTTTTTCTCAATGTATGA
GAGGCGCATTGGAGTTCTGCTGTTGATCTCATTAACACAGACCTGCAGGAAGCG
GCGGCGGAAGTCAGGCATACGCTGGTAACTTTGAGGCAGCTGGTAACGCTCTA
TGATCCAGTCGATTTTCAGAGAGACGATGCCTGAGCCATCCGGCTTACGATACT
GACACAGGGATTCGTATAAACGCATGGCATACGGATTGGTGATTTCTTTTGTTTC
ACTAAGCCGAAACTGCGTAAACCGGTTCTGTAACCCGATAAAGAAGGGAATGAG
ATATGGGTTGATATGTACACTGTAAAGCCCTCTGGATGGACTGTGCGCACGTTT
GATAAACCAAGGAAAAGATTCATAGCCTTTTTCATCGCCGGCATCCTCTTCAGG

GCGATAAAAAACCACTTCCTTCCCCGCGAAACTCTTCAATGCCTGCCGTATATC
CTTACTGGCTTCCGCAGAGGTCAATCCGAATATTTCAGCATATTTAGCAACATGG
ATCTCGCAGATACCGTCATGTTCCTGTAGGGTGCCATCAGATTTTCTGATCTGGT
CAACGAACAGATACAGCATACGTTTTTGATCCCGGGAGAGACTATATGCCGCCT
CAGTGAGGTCGTTTGACTGGACGATTCGCGGGCTATTTTTACGTTTCTTGTGATT
GATAACCGCTGTTTCCGCCATGACAGATCCATGTGAAGTGTGACAAGTTTTTAG
ATTGTCACACTAAATAAAAAAGAGTCAATAAGCAGGGATAACTTTGTGAAAAAAC
AGCTTCTTCTGAGGGCAATTTGTCACAGGGTTAAGGGCAATTTGTCACAGACAG
GACTGTCATTTGAGGGTGATTTGTCACACTGAAAGGGCAATTTGTCACAACACC
TTCTCTAGAACCAGCATGGATAAAGGCCTACAAGGCGCTCTAAAAAAGAAGATC
TAAAAACTATAAAAAAAATAATTATAAAAATATCCCCGTGGATAAGTGGATAACC
CCAAGGGAAGTTTTTTCAGGCATCGTGTGTAAGCAGAATATATAAGTGCTGTTCC
CTGGTGCTTCCTCGCTCACTCGAGGGCTTCGCCCTGTCGCTCAACTGCGGCGA
GCACTACTGGCTGTAAAAGGACAGACCACATCATGGTTCTGTGTTCATTAGGTT
GTTCTGTCCATTGCTGACATAATCCGCTCCACTTCAACGTAACACCGCACGAAG
ATTTCTATTGTTCCTGAAGGCATATTCAAATCGTTTTCGTTACCGCTTGCAGGCA
TCATGACAGAACACTACTTCCTATAAACGCTACACAGGCTCCTGAGATTAATAAT
GCGGATCTCTACGATAATGGGAGATTTTCCCGACTGTTTCGTTCGCTTCTCAGT
GGATAACAGCCAGCTTCTCTGTTTAACAGACAAAAACAGCATATCCACTCAGTTC
CACATTTCCATATAAAGGCCAAGGCATTTATTCTCAGGATAATTGTTTCAGCATC
GCAACCGCATCAGACTCCGGCATCGCAAACTGCACCCGGTGCCGGGCAGCCAC
ATCCAGCGCAAAAACCTTCGTGTAGACTTCCGTTGAACTGATGGACTTATGTCC
CATCAGGCTTTGCAGAACTTTCAGCGGTATACCGGCATACAGCATGTGCATCGC
ATAGGAATGGCGGAACGTATGTGGTGTGACCGGAACAGAGAACGTCACACCGT
CAGCAGCAGCGGCGGCAACCGCCTCCCCAATCCAGGTCCTGACCGTTCTGTCC
GTCACTTCCCAGATCCGCGC

pRC2312

GGTCGGGCTGAACGGGGGGTTCGTGCACACAGCCCAGCTTGGAGCGAACGAC
CTACACCGAACTGAGATACCTACAGCGTGAGCTATGAGAAAGCGCCACGCTTCC
CGAAGGGAGAAAGGCGGACAGGTATCCGGTAAGCGGCAGGGTCGGAACAGGA
GAGCGCACGAGGGAGCTTCCAGGGGGAAACGCCTGGTATCTTTATAGTCCTGT
CGGGTTTCGCCACCTCTGACTTGAGCGTCGATTTTTGTGATGCTCGTCAGGGGG
GCGGAGCCTATGGAAAAACGCCAGCAACGCGGCCTTTTTACGGTTCCTGGCCT
TTTGCTGGCCTTTTGCTCACATGTTCTTTCCTGCGTTATCCCCTGATTCTGTGGA
TAACCGTATTACCGCCTTTGAGTGAGCTGATACCGCTCGCCGCAGCCGAACGAC
CGAGCGCAGCGAGTCAGTGAGCGAGGAAGCGGAAGAGCGCCCAATACGCAAA
CCGCCTCTCCCCGCGCGTTGGCCGATTCATTAATGCAGCTGGCACGACAGGTT
TCCCGACTGGAAAGCGGGCAGTGAGCGCAACGCAATTAATGTGAGTTAGCTCA
CTCATTAGGCACCCCAGGCTTTACACTTTATGCTTCCGGCTCGTATGTTGTGTG
GAATTGTGAGCGGATAACAATTTCACACAGGAAACAGCTATGACCATGATTACG
CCAAGCGCGCAATTAACCCTCACTAAAGGGAACAAAAGCTGGAGCTCCACCGC
GGTGGCGGCCGCTCTAGAACTAGTGGATCCCCCGGGCTGCAGGAATTCGATAT

CAAGCTTATCGATACCGTCGACCTCGAGGGGGGGCCCGGTACCCAATTCGCCC
TATAGTGAGTCGTATTACGCGCGCTCACTGGCCGTCGTTTTACAACGTCGTGAC
TGGGAAAACCCTGGCGTTACCCAACTTAATCGCCTTGCAGCACATCCCCCTTTC
GCCAGCTGGCGTAATAGCGAAGAGGCCCGCACCGATCGCCCTTCCCAACAGTT
GCGCAGCCTGAATGGCGAATGGGACGCGCCCTGTAGCGGCGCATTAAGCGCG
GCGGGTGTGGTGGTTACGCGCAGCGTGACCGCTACACTTGCCAGCGCCCTAGC
GCCCGCTCCTTTCGCTTTCTTCCCTTCCTTTCTCGCCACGTTCGCCGGCTTTCC
CCGTCAAGCTCTAAATCGGGGGCTCCCTTTAGGGTTCCGATTTAGTGCTTTACG
GCACCTCGACCCCAAAAAACTTGATTAGGGTGATGGTTCACGTAGTGCAATTTA
GGTGGCACTTTTCGGGGAAATGTGCGCGGAACCCCTATTTGTTTATTTTTCTAAA
TACATTCAAATATGTATCCGCTCATGAGACAATAACCCTGATAAATGCTTCAATA
ATATTGAAAAAGGAAGAGTATGAGTATTCAACATTTCCGTGTCGCCCTTATTCCC
TTTTTTGCGGCATTTTGCCTTCCTGTTTTTGCTCACCCAGAAACGCTGGTGAAAG
TAAAAGATGCTGAAGATCAGTTGGGTGCACGAGTGGGTTACATCGAACTGGATC
TCAACAGCGGTAAGATCCTTGAGAGTTTTCGCCCCGAAGAACGTTTTCCAATGA
TGAGCACTTTTAAAGTTCTGCTATGTGGCGCGGTATTATCCCGTATTGACGCCG
GGCAAGAGCAACTCGGTCGCCGCATACACTATTCTCAGAATGACTTGGTTGAGT
ACTCACCAGTCACAGAAAAGCATCTTACGGATGGCATGACAGTAAGAGAATTAT
GCAGTGCTGCCATAACCATGAGTGATAACACTGCGGCCAACTTACTTCTGACAA
CGATCGGAGGACCGAAGGAGCTAACCGCTTTTTTGCACAACATGGGGGATCAT
GTAACTCGCCTTGATCGTTGGGAACCGGAGCTGAATGAAGCCATACCAAACGAC
GAGCGTGACACCACGATGCCTGTAGCAATGGCAACAACGTTGCGCAAACTATTA
ACTGGCGAACTACTTACTCTAGCTTCCCGGCAACAATTAATAGACTGGATGGAG
GCGGATAAAGTTGCAGGACCACTTCTGCGCTCGGCCCTTCCGGCTGGCTGGTT
TATTGCTGATAAATCTGGAGCCGGTGAGCGTGGGTCTCGCGGTATCATTGCAGC
ACTGGGGCCAGATGGTAAGCCCTCCCGTATCGTAGTTATCTACACGACGGGGA
GTCAGGCAACTATGGATGAACGAAATAGACAGATCGCTGAGATAGGTGCCTCAC
TGATTAAGCATTGGTAACTGTCAGACCAAGTTTACTCATATATACTTTAGATTGAT
TTAAAACTTCATTTTTAATTTAAAAGGATCTAGGTGAAGATCCTTTTTGATAATCT
CATGACCAAAATCCCTTAACGTGAGTTTTCGTTCCACTGAGCGTCAGACCCCGT
AGAAAAGATCAAAGGATCTTCTTGAGATCCTTTTTTTCTGCGCGTAATCTGCTGC
TTGCAAACAAAAAAACCACCGCTACCAGCGGTGGTTTGTTTGCCGGATCAAGAG
CTACCAACTCTTTTTCCGAAGGTAACTGGCTTCAGCAGAGCGCAGATACCAAAT
ACTGTCCTTCTAGTGTAGCCGTAGTTAGGCCACCACTTCAAGAACTCTGTAGCA
CCGCCTACATACCTCGCTCTGCTAATCCTGTTACCAGTGGCTGCTGCCAGTGGC
GATAAGTCGTGTCTTACCGGGTTGGACTCAAGACGATAGTTACCGGATAAGGCG
CAGCGGTCGGGCTGAACGGGGGGTTCGTGCACACAGCCCAGCTTGGAGCGAA
CGACCTACACCGAACTGAGATACCTACAGCGTGAGCTATGAGAAAGCGCCACG
CTTCCCGAAGGGAGAAAGGCGGACAGGTATCCGGTAAGCGGCAGGGTCGGAA
CAGGAGAGCGCACGAGGGAGCTTCCAGGGGGAAACGCCTGGTATCTTTATAGT
CCTGTCGGGTTTCGCCACCTCTGACTTGAGCGTCGATTTTTGTGATGCTCGTCA
GGGGGGCGGAGCCTATGGAAAAACGCCAGCAACGCGGCCTTTTTACGGTTCCT
GGCCTTTTGCTTATAAGGGATTTTGCCGATTTCGGCCTATTGGTTAAAAAATGAG
CTGATTTAACAAAAATTTAACGCGAATTTTAACAAAATATTAACGCTTACAATTTA

GGTGGCACTTTTCGGGGAAATGTGCGCGGAACCCCTATTTGTTTATTTTTCTAAA
TACATTCAAATATGTATCCGCTCATGAGACAATAACCCTGATAAATGCTTCAATA
ATATTGAAAAAGGAAGAGTATGAGTATTCAACATTTCCGTGTCGCCCTTATTCCC
TTTTTTGCGGCATTTTGCCTTCCTGTTTTTGCTCACCCAGAAACGCTGGTGAAAG
TAAAAGATGCTGAAGATCAGTTGGGTGCACGAGTGGGTTACATCGAACTGGATC
TCAACAGCGGTAAGATCCTTGAGAGTTTTCGCCCCGAAGAACGTTTTCCAATGA
TGAGCACTTTTAAAGTTCTGCTATGTGGCGCGGTATTATCCCGTATTGACGCCG
GGCAAGAGCAACTCGGTCGCCGCATACACTATTCTCAGAATGACTTGGTTGAGT
ACTCACCAGTCACAGAAAAGCATCTTACGGATGGCATGACAGTAAGAGAATTAT
GCAGTGCTGCCATAACCATGAGTGATAACACTGCGGCCAACTTACTTCTGACAA
CGATCGGAGGACCGAAGGAGCTAACCGCTTTTTTGCACAACATGGGGGATCAT
GTAACTCGCCTTGATCGTTGGGAACCGGAGCTGAATGAAGCCATACCAAACGAC
GAGCGTGACACCACGATGCCTGTAGCAATGGCAACAACGTTGCGCAAACTATTA
ACTGGCGAACTACTTACTCTAGCTTCCCGGCAACAATTAATAGACTGGATGGAG
GCGGATAAAGTTGCAGGACCACTTCTGCGCTCGGCCCTTCCGGCTGGCTGGTT
TATTGCTGATAAATCTGGAGCCGGTGAGCGTGGGTCTCGCGGTATCATTGCAGC
ACTGGGGCCAGATGGTAAGCCCTCCCGTATCGTAGTTATCTACACGACGGGGA
GTCAGGCAACTATGGATGAACGAAATAGACAGATCGCTGAGATAGGTGCCTCAC
TGATTAAGCATTGGTAACTGTCAGACCAAGTTTACTCATATATACTTTAGATTGAT
TTAAAACTTCATTTTTAATTTAAAAGGATCTAGGTGAAGATCCTTTTTGATAATCT
CATGACCAAAATCCCTTAACGTGAGTTTTCGTTCCACTGAGCGTCAGACCCCGT
AGAAAAGATCAAAGGATCTTCTTGAGATCCTTTTTTTCTGCGCGTAATCTGCTGC
TTGCAAACAAAAAAACCACCGCTACCAGCGGTGGTTTGTTTGCCGGATCAAGAG
CTACCAACTCTTTTTCCGAAGGTAACTGGCTTCAGCAGAGCGCAGATACCAAAT
ACTGTCCTTCTAGTGTAGCCGTAGTTAGGCCACCACTTCAAGAACTCTGTAGCA
CCGCCTACATACCTCGCTCTGCTAATCCTGTTACCAGTGGCTGCTGCCAGTGGC
GATAAGTCGTGTCTTACCGGGTTGGACTCAAGACGATAGTTACCGGATAAGGCG
CAGC

pRC2313

GGTCGGGCTGAACGGGGGGTTCGTGCACACAGCCCAGCTTGGAGCGAACGAC
CTACACCGAACTGAGATACCTACAGCGTGAGCTATGAGAAAGCGCCACGCTTCC
CGAAGGGAGAAAGGCGGACAGGTATCCGGTAAGCGGCAGGGTCGGAACAGGA
GAGCGCACGAGGGAGCTTCCAGGGGGAAACGCCTGGTATCTTTATAGTCCTGT
CGGGTTTCGCCACCTCTGACTTGAGCGTCGATTTTTGTGATGCTCGTCAGGGGG
GCGGAGCCTATGGAAAAACGCCAGCAACGCGGCCTTTTTACGGTTCCTGGCCT
TTTGCTGGCCTTTTGCTCACATGTGGCCTACGTTTTAATGCCAGCAAAAATGGTG
AATTACCTGGATAATCAACTCACGGGTGGGTTTAATAAACCGCGTCTCCAGATAT
TCATCAGGCTGATGAGCTTGATTGATGGAACCCGGACCGAGCACCAGCGTAGG
GCATAACGTTTGAATAAACGGCGCTTCGGTACAGTAATTCACCACTTCGGTTTTT
GCTCCGAGCAATTTCTCAACCACTTCAACCAGTTGATGATTCGGTGGGCATTCA
TAGCCAGGGATCGGCGGATGCAGCTCGTCGACCGTCAGACGACCCGGCCAGC
GTTCGCTCACCGGAGCCAATGCATCGTTGAGCAAACCATTAAGTTCATTGAGTG

TCATGCCAGGCAGCGGACGAATATCCATATGCAACTCACAGCAAGCGCAAATAC
GGTTAGAAGCGTCGCCACCGTGAATATGCCCGAGGTTGAGCGTAGGGTATGGC
ACGGTAAACGCTTCGTAGTGATAACGTTCTTTCAGGTTATCGCGCAATTGCAAAA
TATGCCCGATGGCGTCGTGCATTAGTTCGATAGCGTTAACTCCGCGTGCTGGAT
CGCTGGAGTGCCCCGACTGGCCCTGAATACGGATGGCGTTAGAGATATGACCT
TTATGTGCGCGTACCGGTTGTAGTGACGTCGGTTCGCCAATGATGGCGCAATCC
GGGCGCAGGGCGGTAGTTTCGGCAAAATAACGCGCTCCGGCCATACTGGTTTC
TTCATCAGCAGTCGCCAGAATGTAGAGCGGTTTTTTCAGTTTCGTGACGTCGAC
ATCGCGTAGCGCATCAAGGATAAACGCAAAAAAGCCTTTCATGTCGGCGGTGCC
TAAGCCGTAAAGCTTGCCGTCATGCTCCGTCAGTGTAAACGGATCGCGCGTCCA
GCGACCGTCATCAAATGGCACCGTATCGGTATGCCCCGCCAGCAACAAGCCGC
CAGCCCCCTGTCCGATACTTGCCAGCATATTGAATTTGTTGCGAGTTCCTGGAA
CAGGCTGCACTTCCACATTGAAGCCCAAATCTTTAAACCAGTCCGCCAGCAGAG
TAATTAAATCTGCATTGCTTTGATCGAGTGCCTCTTCCGTGGCGCTTATTGAAGG
TGTGGCAATCAGAGCGCGGTAAATCTCGATAAATGGCGGTAATTTGTTTTTCATT
GTTGACACACCTCTGGTCATGATAGTATCAATATTCATGCAGTATTTATGAATAA
AAATACACTAACGTTGAGCGTAATAAAACCCACCAGCCGTAAGGTGAATGTTTTA
CGTTTAACCTGGCAACCAGACATAAGAAGGTGAATAGCCCCGATGTTGAATACG
CTGATTGTGGGTGCCAGCGGCTACGCTGGCGCAGAGCTAGTGACCTATGTAAA
TCGCCATCCGCATATGAACATAACCGCTTTGACTGTTTCAGCGCAAAGCAATGA
TGCGGGAAAGTTAATCTCCGATTTGCATCCGCAGCTAAAAGGCATCGTTGATCT
GCCGTTGCAGCCGATGTCGGATATCAGCGAGTTTAGCCCAGGGGTGGACGTAG
TGTTTCTCGCCACCGCCCATGAAGTTAGCCACGATTTAGCGCCGCAGTTTCTTG
AAGCGGGCTGCGTGGTGTTCGACCTTTCCGGCGCGTTTCGTGTTAACGACGCC
ACCTTCTATGAAAAATATTACGGCTTTACCCATCAATACCCGGAACTGTTGGAAC
AGGCAGCCTACGGTCTGGCGGAGTGGTGCGGTAATAAATTAAAAGAAGCGAAT
TTGATTGCGGTGCCGGGCTGTTATCCGACGGCGGCACAGCTGGCGCTGAAACC
GTTGATTGATGCCGATCTTCTTGACCTCAATCAGTGGCCGGTGATCAACGCCAC
CAGCGGCGTGAGCGGTGCAGGGCGTAAAGCGGCCATTTCAAACAGCTTTTGTG
AAGTTAGCCTGCAACCGTATGGCGTCTTTACTCATCGCCATCAACCAGAGATCG
CCACACACCTCGGTGCTGACGTTATCTTCACCCCACATCTGGGCAATTTCCCGC
GCGGCATTCTCGAAACCATTACCTGCCGCCTGAAATCGGGTGTGACCCAGGCG
CAAGTCGCGCAAGTGTTACAGCAGGCGTATGCCCATAAACCGCTGGTGCGGCT
GTATGACAAAGGCGTTCCGGCGCTGAAAAATGTCGTTGGGCTGCCATTTTGCGA
TATCGGGTTTGCCGTTCAGGGCGAGCATCTGATTATTGTGGCGACCGAAGACAA
CTTACTGAAAGGCGCGGCGGCACAAGCGGTACAGTGCGCCAATATTCGTTTCG
GCTATGCGGAAACGCAGTCTCTTATTTAAGGGTGCAATGATGAATCCATTAATTA
TCAAACTGGGCGGCGTACTGCTGGATAGTGAAGAGGCGCTGGAACGTCTGTTT
AGCGCACTGGTGAATTATCGTGAGTCACATCAGCGTCCGCTGGTGATTGTGCAC
GGCGGCGGTTGCGTGGTGGATGAGCTGATGAAAGGGCTGAATCTGCCGGTGAA
AAAGAAAAATGGCCTGCGGGTGACGCCTGCGGATCAGATAGACATTATCACCG
GAGCACTGGCGGGAACGGCAAATAAAACCCTGTTGGCATGGGCGAAGAAACAT
CAGATTGCGGCCGTAGGTTTGTTTCTCGGTGACGGCGACAGCGTCAAAGTGAC
CCAGCTTGATGAAGAGTTAGGCCATGTTGGACTGGCGCAGCCAGGTTCGCCTA

AGCTTATCAACTCCTTGCTGGAGAACGGTTATCTGCCGGTGGTCAGCTCCATTG
GCGTAACAGACGAAGGGCAACTGATGAACGTCAATGCCGACCAGGCGGCAACG
GCGCTGGCGGCAACGCTGGGCGCGGATCTGATTTTGCTCTCCGACGTCAGCGG
CATTCTCGACGGCAAAGGGCAACGCATTGCCGAAATGACCGCCGCGAAAGCAG
AACAACTGATTGAGCAGGGCATTATTACTGACGGCATGATAGTGAAAGTGAACG
CGGCGCTGGATGCGGCCCGCACGCTGGGCCGTCCGGTAGATATCGCCTCCTG
GCGTCATGCGGAGCAGCTTCCGGCACTGTTTAACGGTATGCCGATGGGTACGC
GGATTTTAGCTTAAGTTTTGTTGGCCGGAGGCGCAGCTTTCCGGCATTGAATTT
CAAAATAAGGAAACAGAGTTATGGCACTTTGGGGCGGGCGTTTTACCCAGGCA
GCAGATCAACGGTTCAAACAATTCAACGACTCACTGCGCTTTGATTACCGTCTG
GCGGAGCAGGATATTGTTGGCTCTGTGGCCTGGTCCAAAGCCCTGGTCACGGT
AGGCGTGTTAACCGCAGAAGAGCAGGCGCAACTGGAAGAGGCGCTGAACGTGT
TGCTGGAAGATGTTCGCGCCAGGCCACAACAAATCCTTGAAAGCGACGCCGAA
GATATCCATAGCTGGGTGGAAGGCAAACTGATCGACAAAGTGGGCCAGTTAGG
CAAAAAGCTGCATACCGGGCGTAGCCGTAATGATCAGGTAGCGACTGACCTGA
AACTGTGGTGCAAAGATACCGTTAGCGAGTTACTGACGGCTAACCGGCAGCTG
CAATCGGCGCTGGTGGAAACCGCACAAAACAATCAGGACGCGGTAATGCCAGG
TTACACTCACCTGCAACGCGCCCAGCCGGTGACGTTCGCGCACTGGTGCCTGG
CCTATGTTGAGATGCTGGCGCGTGATGAAAGCCGTTTGCAGGATGCGCTTAAGC
GTCTGGATGTCAGCCCGCTAGGCTGTGGCGCGCTGGCGGGAACGGCCTATGAA
ATCGACCGTGAACAGTTAGCAGGCTGGCTGGGCTTTGCTTCGGCGACCCGTAA
CAGTCTCGACAGCGTTTCTGACCGTGACCATGTGTTGGAACTGCTTTCTGCTGC
CGCTATCGGCATGGTGCATCTGTCGCGTTTTGCTGAAGATCTGATTTTCTTTAAC
ACCGGCGAAGCGGGGTTTGTGGAGCTTTCTGACCGCGTGACTTCCGGTTCATC
ATTAATGCCGCAGAAGAAAAACCCGGATGCGCTGGAGCTGATTCGCGGTAAAT
GCGGCCGGGTGCAGGGGGCGTTAACCGGCATGATGATGACGCTGAAAGGTTT
GCCGCTGGCTTACAACAAAGATATGCAGGAAGACAAAGAAGGTCTGTTCGACG
CGCTCGATACCTGGCTGGACTGCCTGCATATGGCGGCGCTGGTGCTGGACGGC
ATTCAGGTGAAACGTCCACGTTGCCAGGAAGCGGCTCAGCAGGGTTACGCCAA
CGCCACCGAACTGGCGGATTATCTGGTGGCGAAAGGCGTACCGTTCCGCGAGG
CGCACCATATTGTTGGTGAAGCGGTGGTGGAAGCCATTCGTCAGGGCAAACCG
CTGGAAGATCTGCCGCTCAGTGAGTTGCAGAAATTCAGTCAGGTGATTGACGAA
GATGTCTATCCGATTCTGTCGCTGCAATCGTGCCTCGACAAGACATGTTCTTTCC
TGCGTTATCCCCTGATTCTGTGGATAACCGTATTACCGCCTTTGAGTGAGCTGAT
ACCGCTCGCCGCAGCCGAACGACCGAGCGCAGCGAGTCAGTGAGCGAGGAAG
CGGAAGAGCGCCCAATACGCAAACCGCCTCTCCCCGCGCGTTGGCCGATTCAT
TAATGCAGCTGGCACGACAGGTTTCCCGACTGGAAAGCGGGCAGTGAGCGCAA
CGCAATTAATGTGAGTTAGCTCACTCATTAGGCACCCCAGGCTTTACACTTTATG
CTTCCGGCTCGTATGTTGTGTGGAATTGTGAGCGGATAACAATTTCACACAGGA
AACAGCTATGACCATGATTACGCCAAGCGCGCAATTAACCCTCACTAAAGGGAA
CAAAAGCTGGAGCTCCACCGCGGTGGCGGCCGCTCTAGAACTAGTGGATCCCC
CGGGCTGCAGGAATTCGATATCAAGCTTATCGATACCGTCGACCTCGAGGGGG
GGCCCGGTACCCAATTCGCCCTATAGTGAGTCGTATTACGCGCGCTCACTGGC
CGTCGTTTTACAACGTCGTGACTGGGAAAACCCTGGCGTTACCCAACTTAATCG

CCTTGCAGCACATCCCCCTTTCGCCAGCTGGCGTAATAGCGAAGAGGCCCGCA
CCGATCGCCCTTCCCAACAGTTGCGCAGCCTGAATGGCGAATGGGACGCGCCC
TGTAGCGGCGCATTAAGCGCGGCGGGTGTGGTGGTTACGCGCAGCGTGACCG
CTACACTTGCCAGCGCCCTAGCGCCCGCTCCTTTCGCTTTCTTCCCTTCCTTTCT
CGCCACGTTCGCCGGCTTTCCCCGTCAAGCTCTAAATCGGGGGCTCCCTTTAG
GGTTCCGATTTAGTGCTTTACGGCACCTCGACCCCAAAAAACTTGATTAGGGTG
ATGGTTCACGTAGTGCAATTTAGGTGGCACTTTTCGGGGAAATGTGCGCGGAAC
CCCTATTTGTTTATTTTTCTAAATACATTCAAATATGTATCCGCTCATGAGACAAT
AACCCTGATAAATGCTTCAATAATATTGAAAAAGGAAGAGTATGAGTATTCAACA
TTTCCGTGTCGCCCTTATTCCCTTTTTTGCGGCATTTTGCCTTCCTGTTTTTGCTC
ACCCAGAAACGCTGGTGAAAGTAAAAGATGCTGAAGATCAGTTGGGTGCACGA
GTGGGTTACATCGAACTGGATCTCAACAGCGGTAAGATCCTTGAGAGTTTTCGC
CCCGAAGAACGTTTTCCAATGATGAGCACTTTTAAAGTTCTGCTATGTGGCGCG
GTATTATCCCGTATTGACGCCGGGCAAGAGCAACTCGGTCGCCGCATACACTAT
TCTCAGAATGACTTGGTTGAGTACTCACCAGTCACAGAAAAGCATCTTACGGAT
GGCATGACAGTAAGAGAATTATGCAGTGCTGCCATAACCATGAGTGATAACACT
GCGGCCAACTTACTTCTGACAACGATCGGAGGACCGAAGGAGCTAACCGCTTTT
TTGCACAACATGGGGGATCATGTAACTCGCCTTGATCGTTGGGAACCGGAGCTG
AATGAAGCCATACCAAACGACGAGCGTGACACCACGATGCCTGTAGCAATGGC
AACAACGTTGCGCAAACTATTAACTGGCGAACTACTTACTCTAGCTTCCCGGCA
ACAATTAATAGACTGGATGGAGGCGGATAAAGTTGCAGGACCACTTCTGCGCTC
GGCCCTTCCGGCTGGCTGGTTTATTGCTGATAAATCTGGAGCCGGTGAGCGTG
GGTCTCGCGGTATCATTGCAGCACTGGGGCCAGATGGTAAGCCCTCCCGTATC
GTAGTTATCTACACGACGGGGAGTCAGGCAACTATGGATGAACGAAATAGACAG
ATCGCTGAGATAGGTGCCTCACTGATTAAGCATTGGTAACTGTCAGACCAAGTT
TACTCATATATACTTTAGATTGATTTAAAACTTCATTTTTAATTTAAAAGGATCTAG
GTGAAGATCCTTTTTGATAATCTCATGACCAAAATCCCTTAACGTGAGTTTTCGT
TCCACTGAGCGTCAGACCCCGTAGAAAAGATCAAAGGATCTTCTTGAGATCCTT
TTTTTCTGCGCGTAATCTGCTGCTTGCAAACAAAAAAACCACCGCTACCAGCGG
TGGTTTGTTTGCCGGATCAAGAGCTACCAACTCTTTTTCCGAAGGTAACTGGCTT
CAGCAGAGCGCAGATACCAAATACTGTCCTTCTAGTGTAGCCGTAGTTAGGCCA
CCACTTCAAGAACTCTGTAGCACCGCCTACATACCTCGCTCTGCTAATCCTGTTA
CCAGTGGCTGCTGCCAGTGGCGATAAGTCGTGTCTTACCGGGTTGGACTCAAG
ACGATAGTTACCGGATAAGGCGCAGCGGTCGGGCTGAACGGGGGGTTCGTGC
ACACAGCCCAGCTTGGAGCGAACGACCTACACCGAACTGAGATACCTACAGCG
TGAGCTATGAGAAAGCGCCACGCTTCCCGAAGGGAGAAAGGCGGACAGGTATC
CGGTAAGCGGCAGGGTCGGAACAGGAGAGCGCACGAGGGAGCTTCCAGGGGG
AAACGCCTGGTATCTTTATAGTCCTGTCGGGTTTCGCCACCTCTGACTTGAGCG
TCGATTTTTGTGATGCTCGTCAGGGGGGCGGAGCCTATGGAAAAACGCCAGCA
ACGCGGCCTTTTTACGGTTCCTGGCCTTTTGCTTATAAGGGATTTTGCCGATTTC
GGCCTATTGGTTAAAAAATGAGCTGATTTAACAAAAATTTAACGCGAATTTTAAC
AAAATATTAACGCTTACAATTTAGGTGGCACTTTTCGGGGAAATGTGCGCGGAA
CCCCTATTTGTTTATTTTTCTAAATACATTCAAATATGTATCCGCTCATGAGACAA
TAACCCTGATAAATGCTTCAATAATATTGAAAAAGGAAGAGTATGAGTATTCAAC

ATTTCCGTGTCGCCCTTATTCCCTTTTTTGCGGCATTTTGCCTTCCTGTTTTTGCT
CACCCAGAAACGCTGGTGAAAGTAAAAGATGCTGAAGATCAGTTGGGTGCACG
AGTGGGTTACATCGAACTGGATCTCAACAGCGGTAAGATCCTTGAGAGTTTTCG
CCCCGAAGAACGTTTTCCAATGATGAGCACTTTTAAAGTTCTGCTATGTGGCGC
GGTATTATCCCGTATTGACGCCGGGCAAGAGCAACTCGGTCGCCGCATACACTA
TTCTCAGAATGACTTGGTTGAGTACTCACCAGTCACAGAAAAGCATCTTACGGAT
GGCATGACAGTAAGAGAATTATGCAGTGCTGCCATAACCATGAGTGATAACACT
GCGGCCAACTTACTTCTGACAACGATCGGAGGACCGAAGGAGCTAACCGCTTTT
TTGCACAACATGGGGGATCATGTAACTCGCCTTGATCGTTGGGAACCGGAGCTG
AATGAAGCCATACCAAACGACGAGCGTGACACCACGATGCCTGTAGCAATGGC
AACAACGTTGCGCAAACTATTAACTGGCGAACTACTTACTCTAGCTTCCCGGCA
ACAATTAATAGACTGGATGGAGGCGGATAAAGTTGCAGGACCACTTCTGCGCTC
GGCCCTTCCGGCTGGCTGGTTTATTGCTGATAAATCTGGAGCCGGTGAGCGTG
GGTCTCGCGGTATCATTGCAGCACTGGGGCCAGATGGTAAGCCCTCCCGTATC
GTAGTTATCTACACGACGGGGAGTCAGGCAACTATGGATGAACGAAATAGACAG
ATCGCTGAGATAGGTGCCTCACTGATTAAGCATTGGTAACTGTCAGACCAAGTT
TACTCATATATACTTTAGATTGATTTAAAACTTCATTTTTAATTTAAAAGGATCTAG
GTGAAGATCCTTTTTGATAATCTCATGACCAAAATCCCTTAACGTGAGTTTTCGT
TCCACTGAGCGTCAGACCCCGTAGAAAAGATCAAAGGATCTTCTTGAGATCCTT
TTTTTCTGCGCGTAATCTGCTGCTTGCAAACAAAAAAACCACCGCTACCAGCGG
TGGTTTGTTTGCCGGATCAAGAGCTACCAACTCTTTTTCCGAAGGTAACTGGCTT
CAGCAGAGCGCAGATACCAAATACTGTCCTTCTAGTGTAGCCGTAGTTAGGCCA
CCACTTCAAGAACTCTGTAGCACCGCCTACATACCTCGCTCTGCTAATCCTGTTA
CCAGTGGCTGCTGCCAGTGGCGATAAGTCGTGTCTTACCGGGTTGGACTCAAG
ACGATAGTTACCGGATAAGGCGCAGC

Spacer 1a	AAAC AGGCACCCCAGGCTTTACACTTTATGCTTCG
Spacer 1b	AAAACGAAGCATAAAGTGTAAAGCCTGGGGTGCCT
Spacer 2a	AAAC TACGAGCCGGAAGCATAAAGTGTAAAGCCTG
Spacer 2b	AAAACAGGCTTTACACTTTATGCTTCCGGCTCGTA
Spacer 3a	AAAC TCCGGCTCGTATGTTGTGTGAAATTGTGAGG
Spacer 3b	AAAACCTCACAATTTCACACAACATACGAGCCGGA
Spacer 4a	AAAC TCCGCTCACAATTTCACACAACATACGAGCG
Spacer 4b	AAAACGCTCGTATGTTGTGTGAAATTGTGAGCGGA
Spacer 5a	AAAC TGAAATTGTGAGCGGATAACAATTTCACACG
Spacer 5b	AAAACGTGTGAAATTGTTATCCGCTCACAATTTCA
Spacer 6a	AAAC AAACAGCTATGACCATGATTACGGATTCACG
Spacer 6b	AAAACGTGAATCCGTAATCATGGTCATAGCTGTTT
Spacer 7a	AAAC GGTTTTCCCAGTCACGACGTTGTAAAACGAG
Spacer 7b	AAAACTCGTTTTACAACGTCGTGACTGGGAAAACC
Spacer 8a	AAAC TGAATGGCGAATGGCGCTTTGCCTGGTTTCG
Spacer 8b	AAAACGAAACCAGGCAAAGCGCCATTCGCCATTCA
Spacer 9a	AAAC ACCCGTCGGATTCTCCGTGGGAACAAACGGG
Spacer 9b	AAAACCCGTTTGTTCCCACGGAGAATCCGACGGGT
Spacer 10a	AAAC TCACATTTAATGTTGATGAAAGCTGGCTACG
Spacer 10b	AAAACGTAGCCAGCTTTCATCAACATTAAATGTGA

Supplemental Table 2 CRISPR spacer sequences
Sequences of the oligonucleotides that were annealed together to make the oligoduplexes encoding the spacer sequences encoding the ten candidate gRNAs used in Figure 1.

RNC target top	CGGATTCACTGGCCGTCGTTTTACAACGTCGTGACTGGGAAAACCCTGGC	Substrate used in EMSAs to demonstrate reconstituted sgRNA-dCas9-transposase binding to target DNA. Anneals with RNC target bottom. sgRNA hybridizes with underlined sequence.
RNC target bottom	[Cy5]-GCCAGGGTTTTCCCAGTCACGACGTTGTAAAACGACGGCCAGTGAATCCG	Substrate used in EMSAs to demonstrate reconstituted sgRNA-dCas9-transposase binding to target DNA. Anneals with RNA target top
Hsmar1 substrate top	ACTAGTGGATCCCCCGGGCTGCAGGAATTCTATTAGGTTGGTGCAAAAGTAATTGCGGTTTTGGATCCCTGT-[Cy5]	Substrate used in EMSAs to demonstrate transposase binding to inverted terminal repeat (ITR). Anneals with Hsmar1 substrate bottom. ITR is underlined.
Hsmar1 substrate bottom	ACAGGGATCCAAAACCGCAATTACTTTTGCACCAACCTAATAGAATTCCTGCAGCCCGGGGGATCCACTAGT-[Cy5]	Substrate used in EMSAs to demonstrate transposase binding to ITR. Anneals with Hsmar1 substrate top. ITR is underlined.
sgRNA	GUCACGACGUUGUAAAACGA*guuuuagagcua****GAAA****UAGCAAGUUAAAAUAAGGCUAGUCCGUUAUCAACUUGAAAAAGUGGCACCGAGUCGGUGCUUUU*	sgRNA used for reconstitution with purified dCas9-transposase. Green sequence is the spacer (crRNA targeting sequence), Italicized sequence is the scaffold composed of: i) lowercase = crRNA repeat, ii) bold = tetraloop and iii) blue = tracrRNA sequence.

Supplemental Table 3 Oligonucleotide sequences
